# Computer Simulations Support a Morphological Contribution to BDNF Enhancement of Action Potential Generation

**DOI:** 10.3389/fncel.2016.00209

**Published:** 2016-09-14

**Authors:** Domenico F. Galati, Brian G. Hiester, Kevin R. Jones

**Affiliations:** Department of Molecular, Cellular and Developmental Biology, University of Colorado BoulderBoulder, CO, USA

**Keywords:** BDNF, excitatory, inhibitory, plasticity, computational model

## Abstract

Brain-derived neurotrophic factor (BDNF) regulates both action potential (AP) generation and neuron morphology. However, whether BDNF-induced changes in neuron morphology directly impact AP generation is unclear. We quantified BDNF’s effect on cultured cortical neuron morphological parameters and found that BDNF stimulates dendrite growth and addition of dendrites while increasing both excitatory and inhibitory presynaptic inputs in a spatially restricted manner. To gain insight into how these combined changes in neuron structure and synaptic input impact AP generation, we used the morphological parameters we gathered to generate computational models. Simulations suggest that BDNF-induced neuron morphologies generate more APs under a wide variety of conditions. Synapse and dendrite addition have the greatest impact on AP generation. However, subtle alterations in excitatory/inhibitory synapse ratio and strength have a significant impact on AP generation when synaptic activity is low. Consistent with these simulations, BDNF rapidly enhances spontaneous activity in cortical cultures. We propose that BDNF promotes neuron morphologies that are intrinsically more efficient at translating barrages of synaptic activity into APs, which is a previously unexplored aspect of BDNF’s function.

## Introduction

Cerebral cortical pyramidal neurons receive thousands of synaptic inputs, whose summation determines pyramidal neuron AP generation. The summation of excitatory and inhibitory inputs is influenced, in part, by their relative proximity to one another, their absolute proximity to the site of action potential generation and the structure of the dendritic arbor and soma where the inputs converge (Koch et al., 1983; Bush and Sejnowski, 1994; Magee and Johnston, 1995; Mainen and Sejnowski, 1996; Komendantov and Ascoli, 2009; Ferrante et al., 2013). More generally, the spatial arrangement of synapses, dendrites, and the soma can all impact the rate at which neurons generate APs. Therefore, to understand how stimuli influence AP generation by neurons, one must consider whether changes in morphology induced by those stimuli create a morphology that is more or less efficient at generating APs.

Among stimuli known to alter neuron morphology, secreted proteins like brain-derived neurotrophic factor (BDNF) have attracted considerable interest. BDNF regulates the formation, stability and structure of both synapses and dendrites (Cohen-Cory et al., 2010; Park and Poo, 2013; Edelmann et al., 2014; Zagrebelsky and Korte, 2014). Loss of BDNF in neocortical pyramidal neurons decreases dendritic spine density, reduces inhibitory synapse density, and decreases dendritic complexity (Gorski et al., 2003; Kohara et al., 2007; Hong et al., 2008; English et al., 2012). Elevated BDNF signaling increases dendritic spine and inhibitory synapse density and leads to an enlarged and more complex dendritic arbor (McAllister et al., 1995; Huang et al., 1999; Horch et al., 1999; Tyler and Pozzo-Miller, 2001). In addition, BDNF mediates the redistribution of inhibitory synapses onto the somatic, but not dendritic, compartment of individual pyramidal neurons (Bloodgood et al., 2013), and is necessary for the development and maintenance of normal dendritic spine density in individual pyramidal neurons (English et al., 2012). Thus, BDNF causes localized changes in synapse distribution at the level of individual neurons in concert with its effects on dendritic morphology.

In addition to affecting neuron morphology, BDNF alters multiple facets of neuron physiology and synaptic transmission (Gottmann et al., 2009; Bloodgood et al., 2013). BDNF application enhances the frequency and amplitude of excitatory postsynaptic potentials within minutes (Lessmann et al., 1994; Levine et al., 1995), and acute BDNF stimulation rapidly increases the efficacy of synaptic transmission in the central nervous system and at the neuromuscular junction (Lohof et al., 1993; Kang and Schuman, 1995). BDNF also influences synaptic transmission over much longer time scales. BDNF knockout mice exhibit deficits in long-term potentiation (LTP) that can be reversed by exogenous BDNF application or viral-mediated gene transfer (Korte et al., 1995, 1996; Patterson et al., 1996). In cultured neurons, BDNF application leads to addition of both excitatory and inhibitory synapses (Vicario-Abejón et al., 1998), while potentiating both excitatory and inhibitory postsynaptic potentials and causing increased spontaneous generation of APs (Bolton et al., 2000). While changes in intrinsic excitability may contribute to BDNF’s effects on AP generation (Desai et al., 1999), BDNF has also been observed to decrease or have no impact on intrinsic excitability (Bolton et al., 2000; Graves et al., 2016). In summary, the net effects of BDNF on excitability in cortical circuits are complex, including modulation of both excitatory and inhibitory neuron synapse number and strength. In addition to these aspects of neuronal physiology, it is important to examine whether BDNF’s substantial effects on neuron morphology have a positive, negative, or neutral impact on AP generation. Combining biological image analysis with computational modeling of AP generation can provide insight into how neuron morphology impacts neuron physiology (Parekh and Ascoli, 2013).

Here, we use image analysis techniques to quantify how acutely elevated BDNF signaling impacts the morphology of individual cortical neurons. We then use these morphological parameters to generate neuron models for computational simulations of AP generation. These simulations provide insight into how BDNF-induced morphological changes impact AP generation. In particular, the results indicate that the combined growth and spatial reorganization of excitatory and inhibitory synapses onto dendrites is an important mechanism by which secreted factors like BDNF influence neural activity.

## Results

### BDNF Induces Synapse Formation on New Dendrites

**FIGURE 1:**
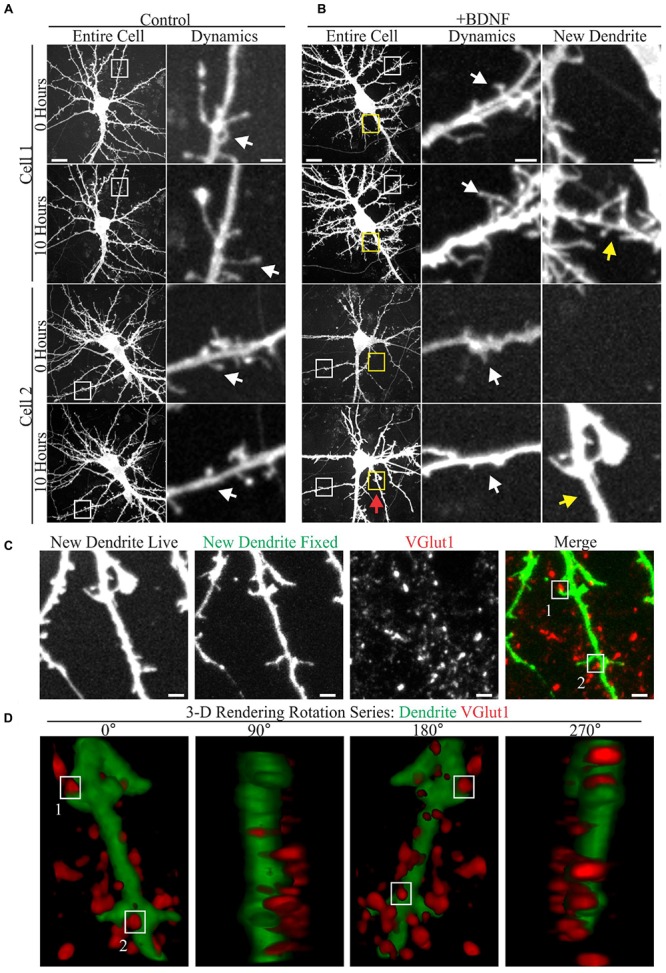
**Brain-derived neurotrophic factor (BDNF) rapidly causes the addition of dendrites and excitatory synapses onto these nascent dendrites. (A,B)** Maximum intensity projections (MIP) of 12 DIV GFP-transfected neurons treated with vehicle **(A)** or 5 ng/ml BDNF for 10 h **(B)** obtained as part of a time-lapse series. The entire cell is shown in the left most columns. Digitally magnified regions (boxes) are shown to the right, documenting the presence of filopodia (white boxes and arrows) and BDNF-induced dendrites (yellow boxes and arrows). Scale bars, 10 and 2 μm. **(C)** MIP of a BDNF induced dendrite that was imaged live and again after fixation and staining with anti-VGlut1 to mark excitatory presynaptic terminals. Nascent terminals on the BDNF-induced dendrite are highlighted by white boxes. The location of the BDNF-induced dendrite is marked by a red arrow in Cell 2 in **(B)**. Scale bar, 2 μm. **(D)** 3-D renderings of the BDNF-induced dendrite shown in **(C)** as a rotation series. White boxes highlight the same two synapses shown in **(C)**.

A few studies have documented the effect of elevated BDNF on living cortical neuron dendrites using time-lapse microscopy (Horch et al., 1999; Horch and Katz, 2002; Tanaka et al., 2008). However, relatively little is known about how low doses of added BDNF influence dendrite structure and new synapse formation in concert. Therefore, we used time-lapse imaging to visualize the response of GFP-transfected cortical neuron dendrites to BDNF (5 ng/ml) and subsequently identified markers for excitatory synapses in the same neurons. Although both vehicle and BDNF-stimulated neurons displayed dynamic dendritic filopodia (**Figures 1A,B**), BDNF stimulated *de novo* dendrite branch formation, rarely observed in control neurons (**Figure 1B**). Most BDNF-treated neurons (90%; 26/29) added at least one dendrite branch during the 10 h observation period, compared to a minority (18%; 5/28) of the vehicle treated neurons. Many nascent BDNF-induced dendrites were decorated with small filopodia having the thin neck and bulbous head characteristic of dendritic spines (**Figure 1B**; arrows), suggesting that excitatory synaptogenesis occurred on the new dendrites. Supporting this possibility, retrospective live-cell microscopy documented the presence of the presynaptic excitatory synapse marker VGlut1 (Rotterman et al., 2014) in close proximity to nascent dendritic spines on BDNF-induced dendrites (**Figure 1C**). Three-dimensional rendering of BDNF-induced dendrites further documented the proximity between VGlut1 and nascent spines (**Figure 1D**), supporting the presence of new excitatory synapses. These results indicate that BDNF can induce synapse formation on new dendrites while it stimulates dendrite growth.

### Altered Synapse Distribution during BDNF-Induced Dendrite Growth

**FIGURE 2:**
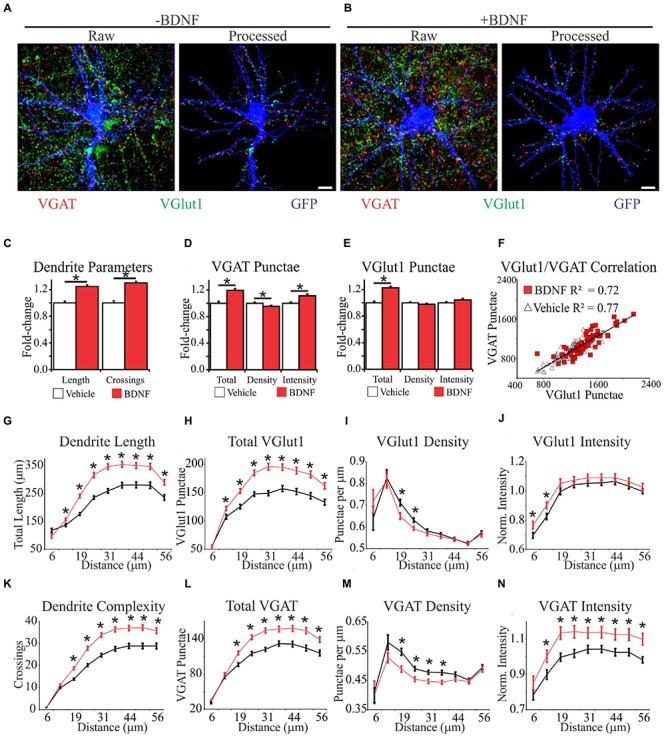
**Brain-derived neurotrophic factor alters the spatial distribution of dendrites and excitatory and inhibitory synapses. (A,B)** Representative MIPs of cortical neurons treated with vehicle **(A)** or BDNF **(B)** for 12 h. In **(A)** and **(B)**, the left images are unprocessed raw data. The right images are segmented data processed with our image analysis routine. Scale bar, 10 μm. **(C–E)** The average morphometric parameters for vehicle-treated (white bars) and BDNF-treated (red bars) neurons. Data are displayed normalized to those of vehicle-treated neurons. Error bars represent SEM; *N* = 63 neurons for both groups. **(F)** Scatter plot showing the correlation between the total number of excitatory and inhibitory synapses on individual vehicle (white triangles) and BDNF-treated (red squares) neurons. **(G–N)** The average morphometric parameters for vehicle-treated (black lines) and BDNF-treated (red lines) neurons binned according to distance from the cell soma as indicated on the *X*-axis. The data for VGlut1 intensity **(J)** and VGAT intensity **(N)** were normalized to vehicle treated values and represented as fold change. Error bars represent SEM; *N* = 63 neurons for both groups ^∗^*p* < 0.05 assessed with unpaired Student’s *T*-test.

To comprehensively determine how synapse abundance and distribution are affected during BDNF-induced dendrite growth and synaptogenesis, we combined immunocytochemistry with an image analysis-assisted approach to dendritic arbor reconstruction. Specifically, we stained for the glutamatergic presynaptic terminal marker VGlut1 and the GABAergic presynaptic terminal marker VGAT (Chaudhry et al., 1998) simultaneously on GFP-labeled fixed cortical neurons. Electrophysiology and functional imaging has been used to ascertain that small clusters of these markers (punctae) reflect active excitatory and inhibitory synapses, so these punctae are hereafter referred to as synapses (Liu, 2004; Jong et al., 2012). To aid our quantification, we developed an automated image analysis routine similar to SynD (Schmitz et al., 2011). Our analysis routine uses an unbiased approach to define dendrite boundaries and soma centers, quantifies these features, and then maps synapse location and intensity in relation to these features (**Figures 2A,B**; **Supplementary Figure S1**). Thus, the output of this analysis routine provides a means to quantify neuron morphology, including the size of the dendritic arbor and distribution of synapses on that arbor, under different conditions.

We first analyzed BDNF effects on the morphology of individual neurons after 12 h of treatment, irrespective of the relative location of the morphological features. Consistent with previous reports (McAllister et al., 1995; Dijkhuizen and Ghosh, 2005), we found that BDNF increased total dendrite length by 24% and dendrite complexity by 30% (**Figure 2C**). BDNF also increased the total number of excitatory and inhibitory synapses (**Figures 2D,E**). However, when synapse abundance was normalized to dendrite length, excitatory synapse density was unaffected, and inhibitory synapse density was modestly decreased (**Figures 2D,E**). The amplitudes of evoked excitatory postsynaptic currents (EPSC) and inhibitory postsynaptic currents (IPSC) are correlated with VGlut1 and VGAT intensity, respectively (Wilson et al., 2005; Tabuchi et al., 2007), allowing puncta intensity to be used as an estimate of presynapse strength (Jong et al., 2012). BDNF significantly increased VGAT punctae intensity (**Figure 2D**), but it did not appreciably impact VGlut1 intensity (**Figure 2E**). The number of excitatory presynaptic punctae typically scales linearly with the number of inhibitory presynaptic punctae on individual neurons in culture (Liu, 2004), consistent with maintaining a balance between excitatory and inhibitory synaptic input. To determine whether this balance is maintained during BDNF stimulation, we plotted the total number of excitatory versus inhibitory synaptic punctae per neuron. Vehicle and BDNF-stimulated neurons both displayed linear excitatory/inhibitory relationships with similar slopes (**Figure 2F**), suggesting that excitatory/inhibitory balance is maintained after BDNF-treatment. We conclude that BDNF stimulation leads to the balanced addition of both excitatory and inhibitory synapses. However, concomitant expansion of the dendritic arbor blunts BDNF’s effect on overall synapse density.

The efficacy of an excitatory synapse refers to the ability of that synapse to drive neuronal membrane potential toward the threshold for AP generation (Williams, 2002). Amongst other factors, synapse efficacy is influenced by the proximity of the synapse to neighboring synapses and the relative distance of the synapse from the site of AP generation (Koch et al., 1983; Qian and Sejnowski, 1990; Bush and Sejnowski, 1994; London et al., 2002; Spruston, 2008; Komendantov and Ascoli, 2009). Therefore, we determined how BDNF stimulation impacts synapse distribution and dendrite morphology in 6 μm annuli centered on the cell soma. BDNF increased dendrite length (**Figure 2G**) and dendrite complexity (**Figure 2K**) in the majority of the dendritic arbor. Similarly, BDNF stimulated the addition of both excitatory and inhibitory synapses in all but the most proximal dendrites (**Figures 2H,L**). However, when synapse abundance was normalized to dendrite length, BDNF decreased excitatory synapse density from 19 to 25 μm from the soma, and even more substantially reduced inhibitory synapse density from 19 to 38 μm (**Figures 2I,M**). BDNF preferentially increased VGlut1 puncta intensity close to the cell soma (<12.5 μm) (**Figure 2J**), and more broadly increased VGAT intensity from 12.5 to 56 μm (**Figure 2N**). Thus, BDNF acutely stimulates the addition and strengthening of excitatory and inhibitory synapses in a spatially restricted manner, while locally decreasing synapse density due to dendrite addition and elongation.

### BDNF-Induced Morphological Alterations Correlate with Increased Network Activity

**FIGURE 3:**
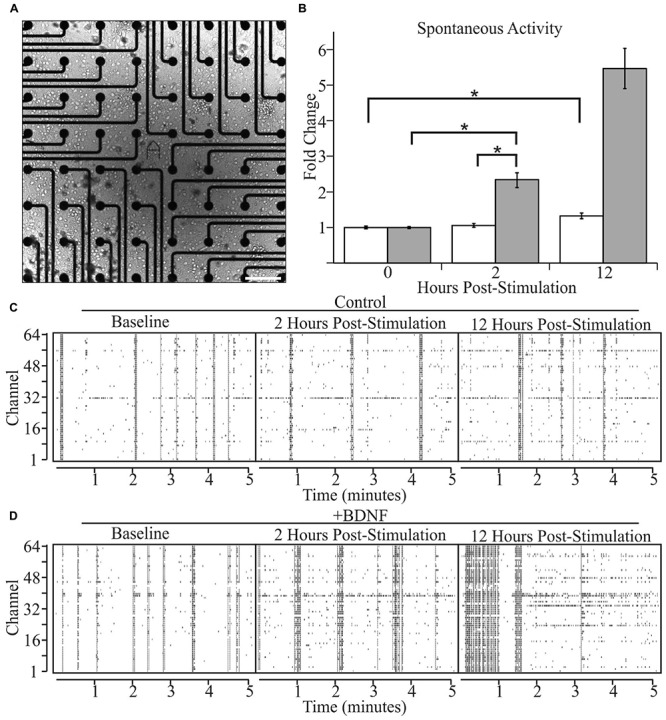
**Brain-derived neurotrophic factor increases spontaneous activity in cultured neurons as determined by multi-electrode arrays. (A)** A bright-field image of a representative cortical culture at 12 DIV on a multi-electrode array. Scale bar equals 200 μm. **(B)** Array-wide spike densities at the indicated time are displayed as fold change relative to the pre-treatment baseline period (0 H) before the addition of vehicle (white bars) or 5 ng/ml BDNF (gray bars) for 12 h. *N* = 36 (12 recordings per time point from three pairs of independent sister cultures). All recording were made during a 2 h window ending at the indicated time. Error bars represent SEM. ^∗^*p* < 0.05 assessed with an unpaired Student’s *T*-test comparing the means of vehicle and BDNF treated neurons. **(C,D)** Raster plots from vehicle-treated **(C)** and BDNF-treated **(D)** sister cultures showing 5 min of representative electrode activity binned into 1 s intervals. Black tick marks indicate action potential activity at a given electrode. The vertical axis denotes the MEA channel from which the recordings were made. The left plots are from the pre-treatment baseline. The middle plots are from 2 h post-treatment. The right plots are from 12 h post-treatment.

We next asked whether altered levels of neuronal activity accompany BDNF-induced changes in neuron morphology. We recorded the spontaneous activity of cultures grown on two-dimensional multi-electrode arrays [**Figure 3A** (phase contrast)] for 2 h, then added BDNF or vehicle and monitored activity 2 and 12 h later. The latter time point was used in the morphological analyses described above. As previously reported (Wagenaar et al., 2006), individual cultures exhibited a range of activity patterns that were dominated by synchronous population-wide bursts of activity (**Figures 3C,D**). Control cultures displayed a non-significant 5% increase in activity after 2 h and a significant, but relatively modest, 32% increase after 12 h, presumably related to continuing maturation of the cultures (**Figure 3B**). In contrast, BDNF stimulation led to large increases in activity (102% at 2 h and 534% by 12 h; **Figure 3B**). These population-wide extracellular recordings are in agreement with single cell patch-clamp studies of cultured neurons, which demonstrated that BDNF increases the rate of AP generation (Bolton et al., 2000). Importantly, our results indicate that the morphological changes induced by BDNF are accompanied by a large increase in the level of spontaneous activity.

### Morphologically Constrained BDNF-Stimulated Models Are More Efficient at Generating APs

Theoretical and experimental evidence indicates that neuron morphology impacts AP generation (Bush and Sejnowski, 1994; Mainen and Sejnowski, 1996; Katz et al., 2009; Komendantov and Ascoli, 2009; Amatrudo et al., 2012; Behabadi et al., 2012; Tejada et al., 2012; Ferrante et al., 2013; Lehnert et al., 2014). Since changes in intrinsic excitability alone are unlikely to explain BDNF’s effects on AP generation (Bolton et al., 2000; Graves et al., 2016), we wanted to determine whether BDNF’s combined effects on neuron morphology could influence the rate of AP generation. To evaluate this possibility, we simulated APs in computational model neurons whose construction was guided by the morphology and excitatory/inhibitory synapse distribution determined from cultured neurons as described above, both with and without BDNF stimulation.

We generated models using the 63 independent vehicle-stimulated (-BDNF) and 63 independent BDNF-stimulated (+BDNF) neurons that were quantified in our morphological analyses described in **Figure 2**. These models contain nine morphological regions that recapitulate the 6.25 μm annuli used in the morphological analyses (a graphical summary of how the models were generated is provided in **Supplementary Figure S2**). The following assumptions were made within each region in order to construct the models from the data: (1) the number of 6.25 μm long dendrites equals the number of annulus crossings determined via Sholl analysis, (2) dendrites are cylinders with a uniform diameter, (3) synapses are uniformly distributed on each dendrite segment within a region and the total number of synapses is the sum of VGlut1 and VGAT punctae, (4) the ratio of excitatory to inhibitory synapses is the ratio of VGlut1 to VGAT punctae, and (5) peak postsynaptic conductance reflects VGlut1 and VGAT punctae intensity. For each model neuron, the decay constant for excitatory and inhibitory postsynaptic potentials, the non-synaptic conductance (i.e., dendrite excitability), the size of the apical dendrite (to account for dendritic material not captured in our image analysis), and the axon was not constrained by our own empirical data. Therefore, the values for these “unconstrained parameters” (i.e., not constrained in the models using the morphological data we gathered as described above) were identical for each series of simulations. Thus, the simulations assume that BDNF does not alter the intrinsic excitability of the neuronal membrane and that BDNF only influences the amount of dendritic material and the number and peak conductance of ionotropic excitatory and inhibitory synapses.

**FIGURE 4:**
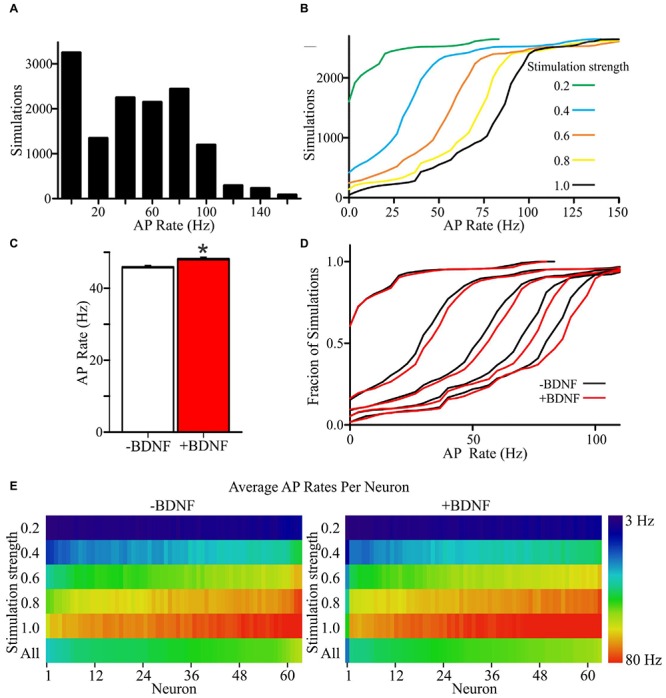
**+Brain-derived neurotrophic factor model neurons exhibit an overall increase in APs relative to -BDNF model neurons. (A)** A histogram shows the distribution of AP rates for all simulations. **(B)** Cumulative frequency plots for all simulations at each stimulation strength, with a stimulation strength of 1.0 representing the activation of all synaptic inputs. **(C)** The group average for the +BDNF simulations (red bar) is significantly elevated relative to -BDNF simulations (white bar). Error bars represent SEM; *N* = 6615 simulations per group; unpaired Students *t*-test; ^∗^*p* < 0.05. **(D)** Relative cumulative frequency plots demonstrate the increased firing in the +BDNF models (red lines) relative to the -BDNF models (black lines) at most stimulation strengths (0.2 to 1.0, increasing from left to right as in **B**). **(E)** Heat maps showing the average AP rate for every model neuron at all stimulation strengths (*n* = 63 each, -BDNF and +BDNF). For the purposes of ordering the model neurons in the heat map, the neurons were ranked according to their average firing rate across all simulations (“All”; bottom row). The rate of AP generation is color coded according to the scale at the right.

To determine the propensity of model neurons to generate APs, different percentages of a model neuron’s synapses were randomly activated. Since the values chosen for unconstrained parameters will impact the rate of AP generation, simulations were performed using a range of values for each unconstrained parameter (Supplementary Tables S1 and **S2**). Ultimately, this approach led to each individual neuron model being simulated across 105 unique combinations of parameter values for a total of 6615 simulations per group (**Figures 4A,B**). The models fired APs at rates from <1 Hz to approximately 100 Hz (**Supplementary Figures S3A,B**), which is within the range of firing rates documented for individual cortical neurons as measured experimentally and modeled in other simulations (Hromádka et al., 2008; O’Connor et al., 2010; Roxin et al., 2011). For both the -BDNF and +BDNF models, the rate of AP generation was elevated by increasing the stimulation strength, reducing the size of the apical dendrite, decreasing the inhibitory postsynaptic potential decay constant, increasing the excitatory postsynaptic potential decay constant, decreasing the time interval within which synapses were activated, and increasing the excitability of the dendritic membrane (**Supplementary Figures S3C–G**). Overall, as unconstrained parameters were modulated, the rate with which -BDNF and +BDNF models generated APs tended to vary in the same direction and with similar magnitudes. Thus, BDNF’s gross effects on neuron morphology do not substantially alter the input–output relationship of model neurons.

**FIGURE 5:**
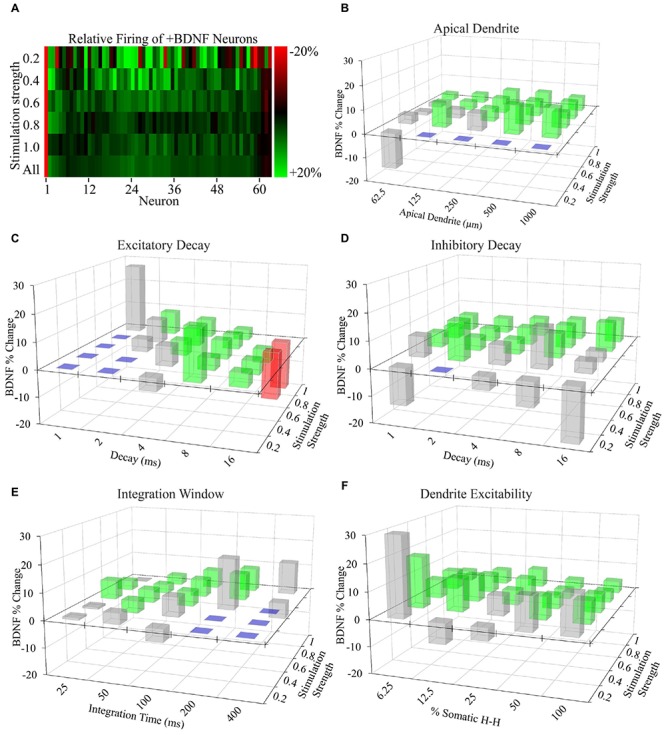
**Increased AP generation by +BDNF model neurons is robust with respect to non-empirically based parameter values. (A)** Heat map showing the average difference between the ranked -BDNF and +BDNF model neurons as displayed in **Figure 4E**. The differences are represented as the % difference between the +BDNF model neuron and its corresponding -BDNF model neuron counterpart from the rank order of **Figure 4E**. **(B–F)** Testing the effects of “unconstrained parameter” value ranges from the literature on the behavior of model neurons. Three-D bar graphs showing the percentage change between the average rate of AP generation for the entire group of +BDNF and -BDNF model neurons for each non-empirically based parameter value tested. Green bars indicate parameter values where the average +BDNF models produced significantly more APs than the average -BDNF models (*p* < 0.05). Red bars indicate parameter values where the +BDNF models produced significantly fewer APs than the average -BDNF models (*p* < 0.05). Gray bars indicate parameter values for which there was no significant difference in AP generation between the average ±BDNF models (*p* > 0.05). Purple squares indicate that less than half of the neurons produced a single AP at this parameter value, and thus comparisons were not made. Statistical significance was assessed with unpaired *t*-tests comparing the average AP rate for the -BDNF models and the +BDNF models. *N* = 63 model neurons per comparison.

To determine whether more subtle differences exist between +BDNF and -BDNF model neurons, we compared their average AP frequency across all simulations. The group average for all +BDNF simulations was modestly, but significantly, elevated compared to the -BDNF simulations (**Figure 4C**), and this enhancement was evident at all but the lowest stimulation intensities (**Figures 4D,E**). Increased AP generation in the +BDNF group could stem from exaggerated firing at a few unconstrained parameter values, or from elevated firing under many different conditions. However, the +BDNF group of neuron models produced significantly more APs in 60 of the 105 combinations of unconstrained parameters (**Figures 5A–F**), with the increases ranging from 3 to 19%. Of the remaining 45 combinations, there were 43 combinations that did not produce significant differences between the groups and only two instances where the +BDNF group of neuron models produced significantly fewer APs. Collectively, these simulations argue that elevated BDNF signaling leads to dendrite architecture and accompanying synapse distribution that generates more APs in response to stochastic activation of synaptic inputs.

### BDNF-Induced Expansion of the Dendritic Arbor Limits the Functional Impact of Synapse Addition

From the simulations described thus far it was unclear which of the morphological parameters that change in response to BDNF addition cause the increase in AP generation. Thus, we sought to test individually which morphological parameter(s) are responsible for the ±BDNF differences in AP generation. First, we determined an average value for each morphological parameter (i.e., number of dendrites, total number of synapses, ratio of excitatory to inhibitory synapses, etc) from the -BDNF group of neurons analyzed. We then substituted these average -BDNF parameter values one at a time into every model neuron in the +BDNF group and ran simulations with these “hybrid” model neurons. We reasoned that if substitution of a -BDNF average parameter value into the +BDNF group of models decreases their rate of AP generation, the +BDNF values for that parameter must contribute to increased AP generation. For these simulations, we set the unconstrained parameter values (i.e., decay constants, dendrite excitability, apical dendrite size and the time interval for integrating synaptic inputs) to their middle values.

**FIGURE 6:**
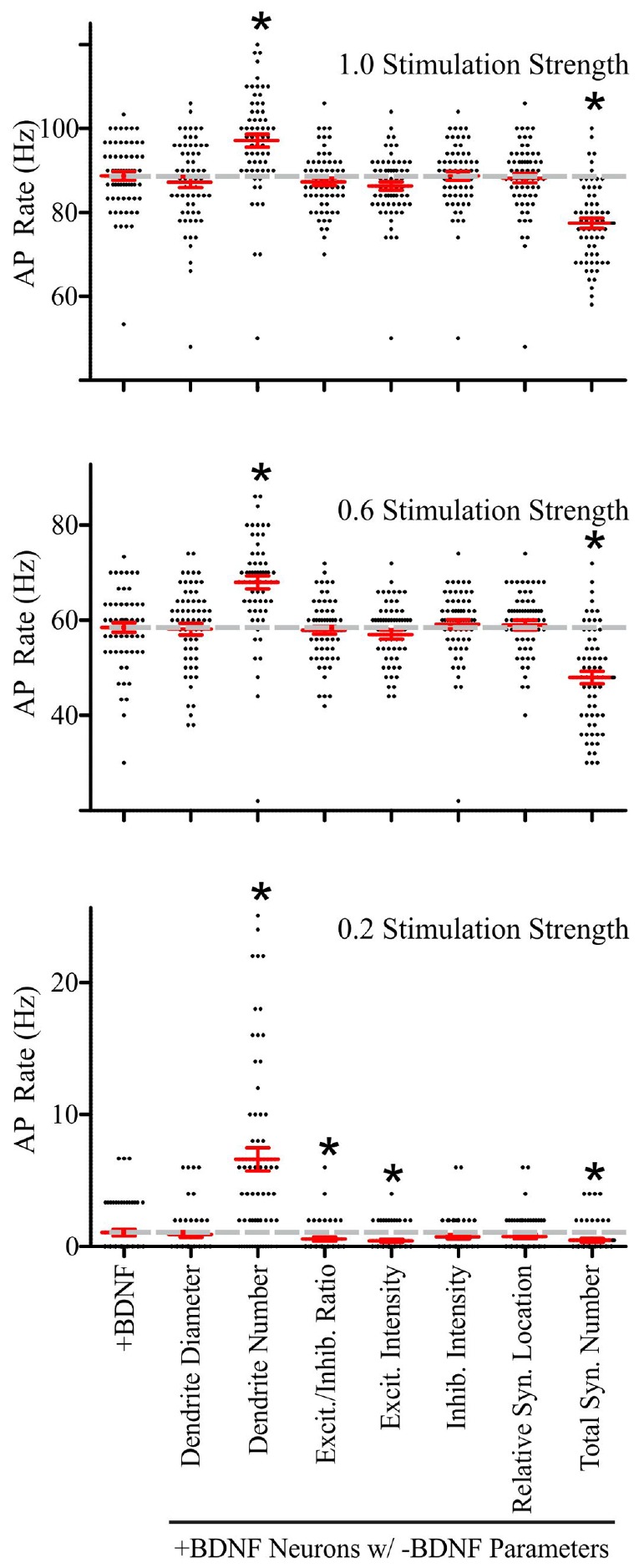
**Brain-derived neurotrophic factor-induced dendrite and synapse addition have the greatest impact on the rate of simulated AP generation in +BDNF model neurons.** Scatter plots showing the distribution (black dots), mean (red horizontal line) and SEM (red “T” lines) for individual +BDNF model neurons (1.0, 0.6, and 0.2 stimulation strengths; top to bottom) where the morphological parameters indicated on the *x*-axis from the -BDNF model neurons were averaged and substituted into the +BDNF model neurons. The AP rate for +BDNF model neurons that were not subjected to substitution is shown in the left-most column, and the average AP rate for these neurons is also shown as a dashed gray line to facilitate comparison. Substituting the number of dendrites from the -BDNF model neurons significantly elevates the rate of AP generation in +BDNF model neurons across all stimulation strengths (third column from left). Substituting the total number of synapses from the -BDNF model neurons significantly decreases the rate of AP generation in +BDNF model neurons across all stimulation strengths (right-most column). Substituting the ratio of excitatory to inhibitory synapses (fourth column from left) and the excitatory synapse intensity (fifth column from left), which is a surrogate for synapse strength, significantly decreases the rate of AP generation in +BDNF model neurons only at the lowest stimulation strength. For stimulation strengths of 1.0 and 0.6, statistical significance was assessed with an unpaired Student’s *t*-test compared to the unsubstituted +BDNF model neurons; *p* < 0.05. For the stimulation strength of 0.2, the data were not normally distributed so statistical significance was assessed with a Wilcoxon ranked-sign test; ^∗^*p* < 0.05. *N* = 63 model neurons per comparison.

Performing these parameter substitutions did not alter the general input–output relationship of our simulations; increasing the stimulation strength elevated the rate of AP generation in a manner that was similar to the unsubstituted +BDNF models (**Supplementary Figure S4**). Substituting most of the -BDNF average parameter values did not significantly impact the rate of AP generation (**Supplementary Figure S4**). However, there were two notable exceptions. First, the -BDNF group had significantly fewer dendrites, and substituting this parameter value into the +BDNF models dramatically increased AP generation at all stimulation strengths (**Figure 6**). This substitution is analogous to BDNF-induced synapse addition and BDNF-induced synapse strength changes occurring without concomitant dendrite addition/elongation. Second, the -BDNF group had significantly fewer synapses, and substituting this parameter value dramatically decreased AP generation (**Figure 6**). This substitution would be analogous to BDNF-induced dendrite addition/elongation occurring without concomitant synapse addition. Thus, these modeling results argue that BDNF-induced synapse addition has a considerable positive impact on AP generation, while the concomitant BDNF-stimulated dendrite formation/elongation mitigates this effect.

Although these substitutions indicated that synapse and dendrite addition cause the largest effects on AP generation at all stimulation strengths, the other morphological parameters did influence AP generation at the lowest stimulation strength. Specifically, increased excitatory synapse conductance, based on VGlut1 intensity, and the subtle increase in the ratio of excitatory to inhibitory synapses in the +BDNF neurons result in simulations that generate more APs. These parameters had a particularly strong influence at the lowest stimulation strength when 20% of the synaptic inputs were active (**Figure 6**). Collectively, these results indicate that although BDNF-induced addition of inhibitory synapses and new dendrites, as well as growth of existing dendrites all tend to reduce the likelihood of AP firing, the concomitant addition and strengthening of excitatory synapses combined with the resulting new synapse distribution lead to a net increase in the likelihood of AP generation, causing the increased firing of APs observed after BDNF addition.

## Discussion

Many studies have quantified the extent to which elevated BDNF leads to dendrite and synapse formation. However, to our knowledge, our study is the first attempt to simultaneously quantify synapse addition along with the location of the added dendrites and synapses across the entire dendritic arbor. These combined observations have led to two notable findings. First, we found that acute BDNF stimulation leads to synapse addition without increasing synapse density on a per neuron basis because synapse addition occurs concomitantly with expansion of the dendritic arbor. Second, modeling neuron behavior using these anatomical parameters suggests that the BDNF-remodeled neurons are overall more likely to fire action potentials with a given level of presynaptic input activity. Below, we discuss these findings in the context of the rich literature surrounding BDNF’s effects on neuron morphology and the emerging appreciation for using computational tools to understand how neuron morphology impacts neuron physiology.

### Integrating BDNF’s Many Effects on Cortical Neuron Morphology

The relationship between BDNF-induced excitatory synapse formation and dendrite formation has been evaluated using time lapse microscopy in a number of different systems, leading to differing conclusions. In *Xenopus*, BDNF infusion increased synapse formation in the optic tectum by enhancing retinal ganglion cell axonal arborization but not tectal neuron dendrite arborization (Sanchez et al., 2006; Cohen-Cory et al., 2010). In visual cortex slice cultures, BDNF over-expression increased the number of dendrites, but it reduced dendritic spine density (Horch et al., 1999; Horch and Katz, 2002). However, dendritic spine analysis was restricted to pre-existing dendrites, and, consequently, the emergence of new spines on new dendrites was not documented. Therefore, our live-cell observations represent new evidence that BDNF induces the formation of dendritic spine synapses on nascent dendritic branches.

In addition to its importance in shaping dendritic trees, dendritic spines, and the excitatory synapses that form on those spines, the role of BDNF in modifying inhibitory synapses has been of great interest. In hippocampal neurons, BDNF application decreased the density of GABA_A_ receptor clusters and VGAT presynaptic terminals per unit length of dendrite segment (Elmariah et al., 2004; Singh et al., 2006), which led to the conclusion that BDNF promotes inhibitory synapse disassembly. However, BDNF application is also reported to increase the number of hippocampal dendrites in culture (Dijkhuizen and Ghosh, 2005; Singh et al., 2006), which complicates conclusions drawn about effects on net synapse formation using puncta per dendrite length measurements. By summing the number of VGAT punctae across entire neurons, we found that BDNF application increased inhibitory synapse formation but slightly decreased inhibitory synapse density because dendrite elongation was more rapid than synapse formation. Therefore, rather than promoting inhibitory synapse disassembly, we suggest that these previously reported results are consistent with ours, and that BDNF-induced inhibitory synapse formation is robust but lags behind dendrite growth and new dendrite formation. Accordingly, when quantified using a microscopic field of view approach to analyze effects on inhibitory synapse markers (Huang et al., 1999; Marty et al., 2000), BDNF increased the density of GAD65 punctae per unit area. Inhibitory synapses limit calcium (Ca^2+^) eﬄux from dendritic spines into the dendritic shaft in a spatially restricted manner (Miles et al., 1996; Chiu et al., 2013). Since dendritic Ca^2+^ participates in activity-dependent dendrite stabilization (Lohmann et al., 2002), temporarily reduced inhibitory synapse density may facilitate the activity-dependent stabilization of new dendrites by allowing greater Ca^2+^ entry induced by excitatory synaptic transmission.

Inhibitory synapses in distinct domains of the dendritic arbor have different functions. For example, dendritic inhibition appears to be most effective at controlling the number of spikes within a pyramidal cell burst (Lovett-Barron et al., 2012; Royer et al., 2012), while perisomatic inhibition regulates the timing of spikes during pyramidal cell bursting (Losonczy et al., 2010; Lovett-Barron and Losonczy, 2014). Recently, it has been shown that BDNF is a critical mediator of location dependent inhibition (Bloodgood et al., 2013). Specifically, activity-dependent expression of BDNF was shown to be necessary for the formation of inhibitory synapses onto the perisomatic region of excitatory neurons (Bloodgood et al., 2013). Similar to these findings, we observed a net increase in the number of inhibitory synapses in the most proximal regions of the dendritic arbor after acute stimulation with BDNF. However, when normalized to total dendrite length, we did not detect an overall increase in inhibitory synapse density. Since loss-of-function studies of the role of endogenous BDNF indicate important local actions for BDNF related in part to the subcellular site of BDNF synthesis and release (Kohara et al., 2007; Abidin et al., 2008; English et al., 2012; Liao et al., 2012; Orefice et al., 2013; Edelmann et al., 2015), extracellular manipulation of BDNF and alteration of its expression within neurons themselves may lead to distinct outcomes.

In our current study, BDNF levels were elevated using bath application, which differs from its presumed normal mode of action in the cortex through localized secretion by neurons, although the range of BDNF signals that may be provided by other cell types, such as microglia (Parkhurst et al., 2013), are not well understood. However, our observations regarding BDNF’s effects on dendrite formation and excitatory and inhibitory synapses are consistent with aspects of the phenotype that result from neuronal BDNF overexpression *in vivo* and in culture (Huang et al., 1999; Horch et al., 1999; Horch and Katz, 2002; Hiester et al., 2013), as well as other reports of the effects of bath application of BDNF (Vicario-Abejón et al., 1998; Alsina et al., 2001; Sanchez et al., 2006; McAllister, 2007; Cohen-Cory et al., 2010; Ivenshitz and Segal, 2010). Moreover, the effects of bath applied BDNF are generally opposite those observed after cortical BDNF-deletion, which reduces inhibitory synapse density and causes dendritic arbors to shrink (Gorski et al., 2003; Kohara et al., 2007; Hong et al., 2008; English et al., 2012; Bloodgood et al., 2013). However, our current study is at odds with the effects that BDNF deletion has on dendritic spine density. *In vivo*, loss of BDNF decreases cortical dendritic spine density (Gorski et al., 2003; English et al., 2012), whereas our analyses of VGlut1 punctae do not reveal increased excitatory synapse density with added BDNF. This discrepancy may indicate that BDNF-supported excitatory synapse formation requires precise patterns of neuronal activity, much like BDNF-dependent modulation of LTP and dendritic spine size (Tanaka et al., 2008; Edelmann et al., 2015), and that bath applied BDNF has limited effects on synapse density due to its lack of temporal or spatial coordination with presynaptic and postsynaptic neural activity. Regardless, as the ability to manipulate BDNF signaling at individual synapses evolves, it will be important to consider how synapse specific release alters the total number of synapses and their location within the dendritic arbor. Moreover, since BDNF-based therapies may rely on non-synapse specific BDNF signaling augmentation (Nagahara et al., 2009; Nagahara and Tuszynski, 2011), it is important to gain a broad understanding of how elevated BDNF generally impacts multiple facets of neuron physiology.

### Using Computational Approaches to Probe BDNF-Induced, Location-Dependent Synapse Addition

The location of presynaptic inputs onto postsynaptic neurons can have a profound effect on synaptic integration and ultimately AP generation (Magee, 2000; London and Häusser, 2005; Spruston, 2008). In our study, we used semi-automated image analysis to provide a detailed morphological analysis of the location-dependent addition of excitatory and inhibitory synapses in response to BDNF. Furthermore, we used morphologically constrained computer simulations to understand how the spatially restricted addition of synapses coalesces to impact action potential generation.

Manual analyses of synaptic punctae in immunofluorescent images are time consuming and subject to human bias. Accordingly, a number of image analysis procedures have been developed to facilitate the rapid and unbiased quantification of neuron morphology (Schmitz et al., 2011; Soto et al., 2011; Billeci et al., 2013; Danielson and Lee, 2014). Here we have extended these previously developed approaches in three important ways. First, we used an intensity-independent method for segmenting synaptic punctae that relies on Laplacian convolution to define punctae edges, which obviates the need for manual threshold selection. Second, we circumvented the need for manual neurite tracing by using intensity-independent Laplacian convolution to define all objects that are continuous with the neuronal soma. It should be noted that the latter technique is sensitive to contamination by neighboring somas, dendrites, and axons, limiting the use of this method to well-separated neurons. Finally, we used a computationally efficient Euclidean distance transformation to track the position of synaptic punctae relative to the neuronal soma. Thus, although there are limitations to the use of our combined approach, it represents an unbiased and relatively efficient means of quantifying neuronal morphology, including synapse distribution.

Computational studies have interrogated how dendrite morphology impacts synaptic integration by using cable models to simulate action potentials (Koch et al., 1983; Bush and Sejnowski, 1994; Komendantov and Ascoli, 2009; Behabadi et al., 2012; Jadi et al., 2012; Ferrante et al., 2013). In general, these results have provided strong evidence that the spatial distribution of excitatory and inhibitory synapses influences the summation of postsynaptic potentials within individual dendrites. In these studies the ratio of excitatory to inhibitory synapses was typically held constant or was systematically varied based upon information from the literature. Our simulations differ from this previous work in that they incorporate excitatory and inhibitory synapse ratios gathered directly from neurons that either did or did not undergo stimulus-induced morphological changes. However, the computer simulations in the present study simplified BDNF’s effects on dendrite geometry using a Sholl analysis based approach, which ignores dendrite parameters such as branch angle and tapering. Going forward it will be important to incorporate these more nuanced effects on dendrite geometry, which can have a substantial impact on AP generation.

Brain-derived neurotrophic factor application enhances the rate of action potential generation in cultured neurons (Levine et al., 1995; Vicario-Abejón et al., 1998; Bolton et al., 2000). BDNF increases mEPSP amplitude and frequency (Lessmann et al., 1994; Levine et al., 1995; Carmignoto et al., 1997), the number of docked presynaptic vesicles at dendritic spine synapses as well as the number of dendritic spine synapses (Tyler and Pozzo-Miller, 2001), and the expression and synaptic localization of proteins that positively regulate excitatory synapse strength [for review see (Carvalho et al., 2008; Gottmann et al., 2009)], suggesting that BDNF-induced increased activity could be due to enhancement of excitatory synapse number and function. However, BDNF-induced enlargement of the dendritic arbor and BDNF-induced inhibitory synapse formation are predicted to antagonize BDNF’s effect on excitatory synapses with regard to action potential generation. Our computer simulations suggest that the net morphological effect arising from BDNF stimulation tips the balance in favor of excitation. Moreover, although the overall effect was modest when a large fraction of synapses were stimulated, the effects were more pronounced when a small fraction of synapses were active. This suggests that BDNF-induced morphological alterations play a more significant role in shaping action potential generation when overall network activity is low.

## Conclusion

In this study we have used computational methods to provide a detailed analysis of how BDNF addition alters neuron morphology and to probe how BDNF’s effects on neuron morphology impact neuron function. The continued development of automated methods to quantify neuronal morphology and synapse distribution, combined with the use of these data to develop neuron models, will provide important insights into how genes modulate the activity of neural circuitry. Further, we have shown that this approach can lead to insights that cannot be gained from analysis of synapse density alone.

## Materials and Methods

### Primary Neuron Cultures

The cerebral cortex was isolated from postnatal day 0 (P0) Sprague-Dawley rats using a procedure similar to previously published methods (Huettner and Baughman, 1986; Hiester et al., 2013). Briefly, cortices were pooled, chopped using a sterile scalpel and digested for 45 min in 200 Units of Papain (Worthington, Lakewood, NJ, USA) in Hank’s balanced salt solution containing glucose, calcium and magnesium. Tissue chunks were washed three times with DMEM containing 10% FBS, 100 U/mL penicillin and 100 U/ml streptomycin (Invitrogen, Carlsbad, CA, USA) and dissociated using fire polished pipets. Cell viability was assessed using Trypan Blue exclusion, and 215,000 viable cells were plated onto acid-washed, Poly-D-Lysine-coated (1 mg/ml in an aqueous solution containing 50 mM boric acid and 25 mM sodium tetraborate, pH 8.5) 12 mm glass coverslips in 24 well plates with a final media volume of 500 μl. Twenty-four hours after plating (day *in vitro*, DIV, 1), DMEM media was replaced with 500 μl complete Neurobasal-A culture media [supplemented with 1X B-27, 1X Glutamax, 100 U/ml penicillin and 100 U/ml streptomycin (Invitrogen, Carlsbad, CA, USA)]. Seventy-two hours after plating (DIV 3), glial proliferation was inhibited with 1 mM AraC (Sigma, St. Louis, MO, USA). While monitoring evaporation during preliminary experiments, we observed approximately 5% evaporation for every 7 days of culture. Therefore, every 3–4 days 12.5 μl of 18 mOhm Milli-Q water was added to the cultures. Cultures were maintained at 36.5°C, 5% CO_2_ and 75% humidity. The preparation and culture of primary neurons was carried out in accordance with the recommendations of the University of Colorado-Boulder Institutional Animal Care and Use Committee.

### DNA Transfection and BDNF Stimulation

Plasmid DNA transfections were performed using Lipofectamine 2000 (Invitrogen, Carlsbad, CA, USA) according to the manufacturer’s instructions. Briefly, 0.700 μg of pEGFP-N1 plasmid DNA in 100 μl of supplement free Neurobasal A was mixed with 2.1 μl of Lipofectamine (3:1 Lipofectamine to DNA ratio) in 100 μl of supplement-free Neurobasal-A and incubated for 30 min at room temperature. During the 30 min incubation, 250 μl of conditioned Neurobasal-A was removed from each well and pooled with 250 μl of fresh complete Neurobasal A (for a 1:1 ratio of conditioned to fresh media). After the 30 min incubation, 100 μl of the DNA/Lipofectamine mixture was added to each well and incubated for 2–4 h at which point the transfection media was rapidly removed and replaced with 1:1 conditioned/fresh media. Transfections were performed 12–24 h before the start of BDNF or vehicle additions (11–12 DIV). Lyophilized, BSA-containing BDNF was purchased from R and D systems (Catalog No. 248-BD-005; Minneapolis, MN, USA), diluted to 50 μg/ml in PBS and stored in 10 μl aliquots at -80°C. At the time of BDNF treatment, single aliquots were thawed and diluted into 50 μg/ml bovine serum albumin in PBS (BSA; Sigma, St. Louis, MO, USA) to create a working stock. The working stock was pipetted into cultures to achieve a final concentration of 5 ng/ml and cultures were treated for: 10 h during live cell microscopy analyses (**Figure 1**), 12 h during fixed cell morphological analyses (**Figure 2**), and 12–14 h during MEA recording (**Figure 3**). Control cultures were treated with an equal volume of 50 μg/ml BSA.

### Live Cell Confocal Microscopy

Neurons were cultured and transfected using the procedures described above but 1 × 10^6^ viable cells were plated onto 35 mm glass bottom dishes (P35G-1.5-10; MatTek, Ashland, MA, USA). At 12–14 DIV, dishes were placed into a pre-equilibrated, environmentally controlled stage (5% CO_2_, 50% humidity, 36.5°; Pathology Devices, Westminster, MD, USA) that was attached to a Nikon Eclipse TE2000-U spinning disk confocal microscope (Nikon, Melville, NY, USA). Images were collected with a Cascade II 16bit EM-CCD camera (Photometrics, Tucson, AZ, USA) 40× 0.75 NA objective. 5–10 GFP-positive neurons were identified per dish and their locations were programmed into an automated stage controller run by MetaMorph software (Molecular Devices, Sunnyvale, CA, USA). Images were acquired every 2 h for a total of 10 h as a *Z*-stack with *Z* spacing of 1 μm and 50–150 ms exposures per frame. The first image was acquired when the neurons were identified, then vehicle or BDNF was added immediately after the final neuron was identified, with 10–20 min lapsing between the identification of the first neuron and the application of vehicle or BDNF. Digital images were acquired as 16 bit TIFF files.

### Immunocytochemistry

Cultures were fixed for 15–20 min with 4% paraformaldehyde (Sigma, St. Louis, MO, USA) dissolved in PBS and subsequently permeabilized with 0.1% Triton-X-100 (Sigma, St. Louis, MO, USA) for 10 min. Following permeabilization, cultures were blocked in PBS containing 10% normal goat serum (Invitrogen, Carlsbad, CA, USA) and 0.2% Tween-20 for 1 h, then incubated with primary antibodies diluted in PBS containing 1% normal goat serum and 0.2% Tween-20 (guinea pig anti-VGlut1,Synaptic Systems, Goettingen, Germany;1:3000; rabbit anti-VGAT, Synaptic Systems, Goettingen, Germany, 1:2000; chicken anti-GFP, Abcam, Cambridge, MA, USA 1:2000) for 3 h at room temperature. Secondary antibodies were diluted 1:2000 in PBS containing 1% normal goat serum and 0.2% Tween-20 (goat anti-Guinea Pig-Alexa-647; goat anti-Rabbit-Alexa-555; goat anti-Chicken-Alexa-488; all from Invitrogen, Carlsbad, CA, USA) for 1 h. After each incubation, cultures were rinsed three times with PBS containing 0.2% Tween-20. Coverslips were mounted with Fluoromount G (SouthernBiotech, Birmingham, AL, USA) and slides stored in the dark at 4°C until imaging.

### Fixed-Cell Confocal Microscopy

Cells were imaged using a Leica spinning disk confocal microscope (Leica Microsystems, Buffalo Grove, IL, USA) equipped with a 63X 1.4 N.A. objective and a Hamamatsu ImagEM-CCD camera (Hamamatsu Corp, Bridgewater, NJ, USA; pixel size: 0.25 μm). Alexa-488 was excited with a 488 nm laser, Alexa 555 was excited with a 568 nm laser and Alexa-647 was excited with a 647 nm laser. Image acquisition was controlled with MetaMorph software (Molecular Devices, Sunnyvale, CA, USA) and acquisition parameters were optimized in preliminary experiments to produce limited pixel saturation (<0.5%) and to ensure that channels were free from cross-talk and bleed-through. Acquisition settings were kept constant throughout the imaging sessions. To limit artifacts caused by time-dependent fluctuations in laser intensity, each imaging session was equally divided between coverslips for each experimental condition. Digital images were acquired as 16-bit TIFF files.

### Digital Image Processing and Synapse Quantification

Image processing was performed using a series of custom macros written in the ImageJ macro scripting language and implemented using FIJI (Schindelin et al., 2012). The workflow consists of three sub-streams: (1) Segment the GFP-labeled neuron and create a Euclidean distance map of the neuron, (2) segment synaptic punctae (3) perform object based colocalization between the segmented GFP signal (neuron) and the segmented punctae (synapse) signals. Each sub-stream is further described below. The full code is presented as a Supplemental File (**Supplemental Data Sheet 1**).

#### Segment the GFP-Labeled Neuron and Create a Euclidean Distance Map

The largest continuous cluster of GFP voxels were identified and converted to a 3D skeleton using the following approach. GFP stacks were convolved with a Laplacian of Gaussian [LOG; radius: 1 voxel; (Erik Meijering, 2012)]. The resulting 32-bit transform was duplicated and converted into a maximum Z projection and the mode and standard deviation pixel intensities for the projection were calculated. The 32-bit LOG stack was then converted into a binary 8-bit image by setting the threshold to the mode plus 1/10th of the standard deviation. Continuous clusters of voxels in the 8-bit image were then segmented and the largest cluster (corresponding to the soma and all continuous dendrites) was retained, while all other clusters (corresponding to GFP signal not continuous with the most massive object) were discarded. At this point, images were manually cleared of spurious dendrite and axons from neighboring transfected neurons if any were present. For the purpose of dendrite length determination, a skeleton of the largest structure was created using the 3D skeletonization plugin (Lee et al., 1994). Sholl analysis was performed on the skeleton using the Sholl analysis plugin^1^ with a Sholl radius of 6.25 μm and every skeletonized dendrite that intersected with a Sholl radius was recorded as a dendritic crossing.

The center of the soma was defined and a Euclidean distance map was created. Contrast within the original 16-bit image stack was saturated at 0.4% and converted to an 8-bit image. The saturated 8-bit image stack was then convolved with a heavy LOG (XY radius: 10 pixels, Z radius 1 voxel), to smooth away all but the largest features, and converted to a maximum Z projection. The projection was thresholded using the Renyi entropy function (Sahoo et al., 1997) to create a binary 8-bit image that contained the largest features. The largest continuous cluster of voxels (the somato-dendritic region) was retained and the center of mass for this object was determined and used as the reference point for a Euclidean distance transform (Borgefors, 1996).

#### Segment Synaptic Punctae on GFP-Transfected Neurons

Puncta stacks were convolved with an LOG (radius: 1 voxel) to sharpen puncta boundaries. The resulting 32-bit transforms were convolved with a local maximum filter (Ollion et al., 2013) [xy radius: 2 pixels; z radius: 1 voxel isotropic with respect to absolute size (500 nm × 500 nm)] and a new binary 8-bit image with a single white pixel at each local maxima was created. Local maxima were then used as “seeds” and the LOG image was used as “spots” for seed based region growing (SRG; Ollion et al., 2013). SRG was constrained with a watershed algorithm to prevent the merging of spots in close spatial proximity. The output of SRG was a collection of 3D masks corresponding to each individual synaptic puncta in the entire image. To separate punctae on untransfected neurons from punctae on transfected neurons, object-based colocalization was performed. Synaptic puncta were considered to be synapsing on the GFP labeled neuron if they contained at least one voxel overlap with the segmented GFP signal. Each feature (GFP neuron, VGlut1 synapses and VGAT synapses) was then redirected to the Euclidean distance transform, such that the average intensity of the feature represents the Euclidean distance of the feature from the center of the cell soma. In addition, intensity measurements for synaptic punctae were made by redirecting the VGlut1 and VGAT features to their original, unprocessed 16-bit images. Fluorescence intensity measurements were normalized to the average intensity for control neurons for each imaging session.

For analysis, each neuron was binned into 6.25 μm diameter annuli beginning at the center of the cell soma and the total dendrite length and number of dendrite crossings (from the skeletonized GFP image), the density of synaptic punctae per dendrite length, the total number of synaptic punctae and synaptic punctae intensity were calculated for each neuron. Data were presented as the average value within each annulus.

### Computer Simulations

Mathematical models were implemented with the NEURON simulation environment (version 7.3) on an Intel Core i7 workstation. Multi-compartment models were constructed for 126 individual neurons (63 -BDNF models and 63 +BDNF models; **Supplementary Figure S2**). The full NEURON simulation code is presented as a Supplemental File (**Supplemental Data Sheet 2**). The topology of each model was constrained by morphological data using the following scheme:

$$\begin{array}{c} \begin{array}{c} Dendrite\,\;Cros\,\sin gs=Compartments\\ Compartment_{Length}=6.25\,\;\mu m\\ Compartment_{Diameter}=\sqrt[{2}]{\left(\frac{V}{\pi \,\;6.25}\right)} \end{array} \end{array}$$

where *V* is the total volume of all GFP voxels in that annulus. The first compartment, corresponding to the first 6.25 μm annulus was designated the somatic compartment; the remaining compartments were designated dendritic compartments. In addition, each model received an identical axon (*Length* = 100 μm; *Diameter* = 1 μm; Innocenti et al., 2013) and an identical apical dendrite with a length of 250 μm which is based on cultured rodent cortical neurons (Hiester et al., 2013). Due to limitations in our microscopy setup, we were unable to image the entire apical dendrite. Therefore, we included the size of the apical dendrite as an unconstrained parameter in our simulations. The number of segments for each compartment was computed using the d-lambda rule (Hines and Carnevale, 1997), with d-lambda equal to 0.1. All model neurons were constrained with the biophysical parameters listed in **Supplementary Table S1**. The conductance for each synapse was chosen from the actual distribution of normalized fluorescent intensities for each population of cells, where 1 normalized arbitrary fluorescence unit (AFU) is equal to 1 nS of conductance. Synaptic conductance was capped at 0.25 and 4 nS as ∼99% of the normalized AFU values fell within this range, and this range is within the range of conductances reported in Katz et al. (2009).

At the start of each simulation, active synapses were randomly chosen to fire within a given time period (i.e., temporal integration window). For example, consider a neuron with 1500 synapses. If the stimulation intensity was set to 20% and the temporal integration window was set to 100 ms, then 300 of the neuron’s synapses were activated in a random order during a 100 ms interval. The 300 active synapses were chosen according to their actual morphological synapse distribution. For example, if 10% of neuron A’s synapses were located in the annulus 56 μm from the soma and 60% of the synapses were VGlut1 positive; on average, 30 of the 300 active synapses would be randomly distributed along the compartment 56 μm from the soma, and 18 would have an excitatory profile. Finally, the peak conductance of each synapse was chosen according to normalized fluorescence intensity. For example, if 20% of the VGlut1 punctae 56 μm from soma had a normalized intensity of 1.5 AFU, then 4 of the 18 synapses would have a peak conductance of 1.5 nS. To monitor action potentials, we recorded the number of times that axonal voltage increased above -35 mV during the temporal integration window. To generate action potential firing landscapes (**Figure 4**), we averaged the action potential firing rate for all model neurons (63 -BDNF models or 63 +BDNF models) during a given simulation and plotted the mean firing rate. To compare -BDNF models to +BDNF models (**Figure 5**) we calculated the percentage difference between the average firing rate during a given simulation.

### Multi-Electrode Array Analysis

Cultures were plated on multi-electrode arrays (MEA; Axion Biosystems, Atlanta, GA, USA; Product: M64-GL1-30Pt200) containing 64 evenly spaced platinum electrodes (electrode diameter = 30 μm; center to center spacing = 200 μm) in an 8 by 8 grid. They were allowed to develop until 12–14 days *in vitro* before recording commenced. Three experiments were performed, and each experiment consisted of two cultures prepared from the same pool of cortices (i.e., “sister cultures”), where one culture was stimulated with vehicle and the other culture was stimulated with BDNF. The recording procedure was as follows: Activity was recorded for a 2 h pre-drug baseline (-2 to 0 h) using an MEA recording unit (Muse, Axion Biosystems, Atlanta, GA, USA). After the baseline, cultures were removed from the recording unit, stimulated with vehicle or BDNF, and then activity was recorded for another 2 h (0–2 h), returned the incubator, and then recorded again at 12–14 h. Extreme care was taken when removing dishes from the recording unit and when placing cultures into the recording unit as per Wagenaar et al. (2006). Activity at each electrode was monitored with a sampling frequency of 12.5 kHz and spikes were detected in real time using the Ada Band spike detections system (Axion Biosystems, Atlanta, GA, USA) set to a threshold of five standard deviations. Spikes were summed into 1 s bins and split into individual chunks of 5 min. The post-drug spike rates for an individual culture were normalized to its own pre-drug spike rate, to minimize the impact of variable firing patterns when comparing vehicle to BDNF stimulated cultures. At the end of each run tetrodotoxin (2 μm; Cambridge, MA, USA) was added to each recording dish to ensure that activity was of biological origin.

### Data and Statistical Analysis

All data were analyzed using a combination of Graphpad Prism, Origin Pro and Microsoft Excel. In the case of normally distributed data, statistical comparisons were performed with an unpaired Student’s *T*-test with significance set at *p* < 0.05. In the case of non-normally distributed data, statistical comparisons were performed with a Wilcoxon signed-rank test with significance set at *p* < 0.05. Statistical tests were performed using Graphpad Prism.

## Author Contributions

DG, BH, and KJ conceived the work, interpreted the data and drafted the manuscript. DG acquired and analyzed the data.

## Conflict of Interest Statement

The authors declare that the research was conducted in the absence of any commercial or financial relationships that could be construed as a potential conflict of interest.

## Footnotes

1 http://labs.biology.ucsd.edu/ghosh/software/index.html

## Supplementary material

FIGURE S1 **An overview of the image analysis routine used to analyze and segment images. (A:** Top) The routine used to segment the GFP labeled neuron from background signal. The raw data is convolved with a Laplacian of Gaussian (LOG) kernel (left). The LOG convolution is thresholded (middle) and the thresholded data is treated with a connectedness filter to remove all but the largest connected object. (**A:** Bottom) The procedure used to find the center of the cell soma. The raw data is convolved with a high-radius Gaussian filter (left). A Renyi entropy threshold is applied to the Gaussian image to identify the soma (middle). The centroid of the soma is calculated and used as the center (right). **(B,C)** The procedure used to segment synaptic puncta. The raw data is convolved with a LOG (left). The LOG is thresholded (middle) and each individual feature is assigned a unique identifier (right). The color code in right hand images utilizes a 3-3-2 RGB lookup table where each individual feature is filled with a different color.

FIGURE S2 **Creating numerical representations of individual neurons to use in computer simulations that reflect the distribution of dendrites and synapses calculated with the image analysis routine. (A–E)** Heat maps projected onto a representative neuron depicting the approach used to constrain computational models of AP generation with morphological parameters. The concentric dashed lines mark individual 6.25 μm annuli. The lookup tables (LUTs) below each image correspond to both the neuron image and the heat maps below. **(A)** Each model neuron contains 6.25 μm dendrite segments equal in number to the number of dendrite crossings determined via Sholl analysis. The first domain for each neuron is the somatic compartment. **(B)** The diameter of each dendrite section was determined by calculating the total GFP volume within each Sholl radius, dividing this value by the number dendrite sections for that Sholl radius and solving for the diameter of the resulting cylinder. The LUT is truncated at 5 μm to preserve subtle differences in dendrite diameter throughout the arbor. **(C)** The total number of excitatory and inhibitory synapses is summed across the entire arbor. **(D)** Individual active synapses are assigned a location based on the relative distribution of synapses throughout the dendritic arbor. This strategy was necessary in order to randomize the process of stimulating synapses when the strength of stimulation was less than 100%. The LUT represents the probability of an active synapse being placed at a specific distance from the soma. **(E)** Individual synapses were assigned an excitatory (0 mV) or inhibitory (-70 mV) reversal potential according to the ratio of VGlut1 to VGAT punctae within each Sholl radius. The conductance of each synapse was randomly assigned using the normalized frequency distribution of synaptic puncta intensities which is similar to the procedure used by Katz et al. (2009). **(F,G)** Heat maps representing the combination of sections, section diameters, synapses, synapse locations, and synapse types across the first 56 μm for each of the 126 individual neurons that were analyzed in this study. The text files used to generate these heat maps were directly imported into NEURON during simulations.

FIGURE S3 **Computational neuron model construction based on morphometric data and model action potential generation rates with a broad range of input parameter values. (A,B)** Representative voltage traces for individual models representing a -BDNF neuron **(A)** and a +BDNF neuron **(B)**. The voltage traces are representative plots from the cable models generated when each of the parameter values were set to their mid-value. The percentage on the left side of each plot refers to the percentage of synapses that were randomly activated during the simulation. **(C–G)** Contour plots showing the average firing rate for the 63 -BDNF model neurons and 63 +BDNF model neurons as the indicated parameter was varied over a range of values.

FIGURE S4 **Substituting the average morphological parameter values from the -BDNF model neurons into the +BDNF model neurons does not dramatically alter the input–output relationship of the simulations. (A–H)** The average AP rates and standard deviations for the +BDNF model neurons after substituting each of the -BDNF morphological parameter values across all stimulation strengths. The rates for unsubstituted +BDNF model neurons are shown in **(A)** and are reproduced in red for each substitution for comparison **(B–H)**.

TABLE S1 **A list of the parameter values used for aspects of the computer simulations that were not based on empirical morphological data**.

TABLE S2 **The range of parameter values used to evaluate the robustness of the computer simulations**.

DATA SHEET 1 **The FIJI/ImageJ macro code used to perform automated image analysis of dendrites and synapses**.

DATA SHEET 2 **The NEURON code used to generate the computer simulations**.
