# Supplementary figures and images for: Computer Simulations Support a Morphological Contribution to BDNF Enhancement of Action Potential Generation
Source: Front Cell Neurosci. 2016 Sep 14;10:209. doi: 10.3389/fncel.2016.00209 (PMC5021759; doi:10.3389/fncel.2016.00209)

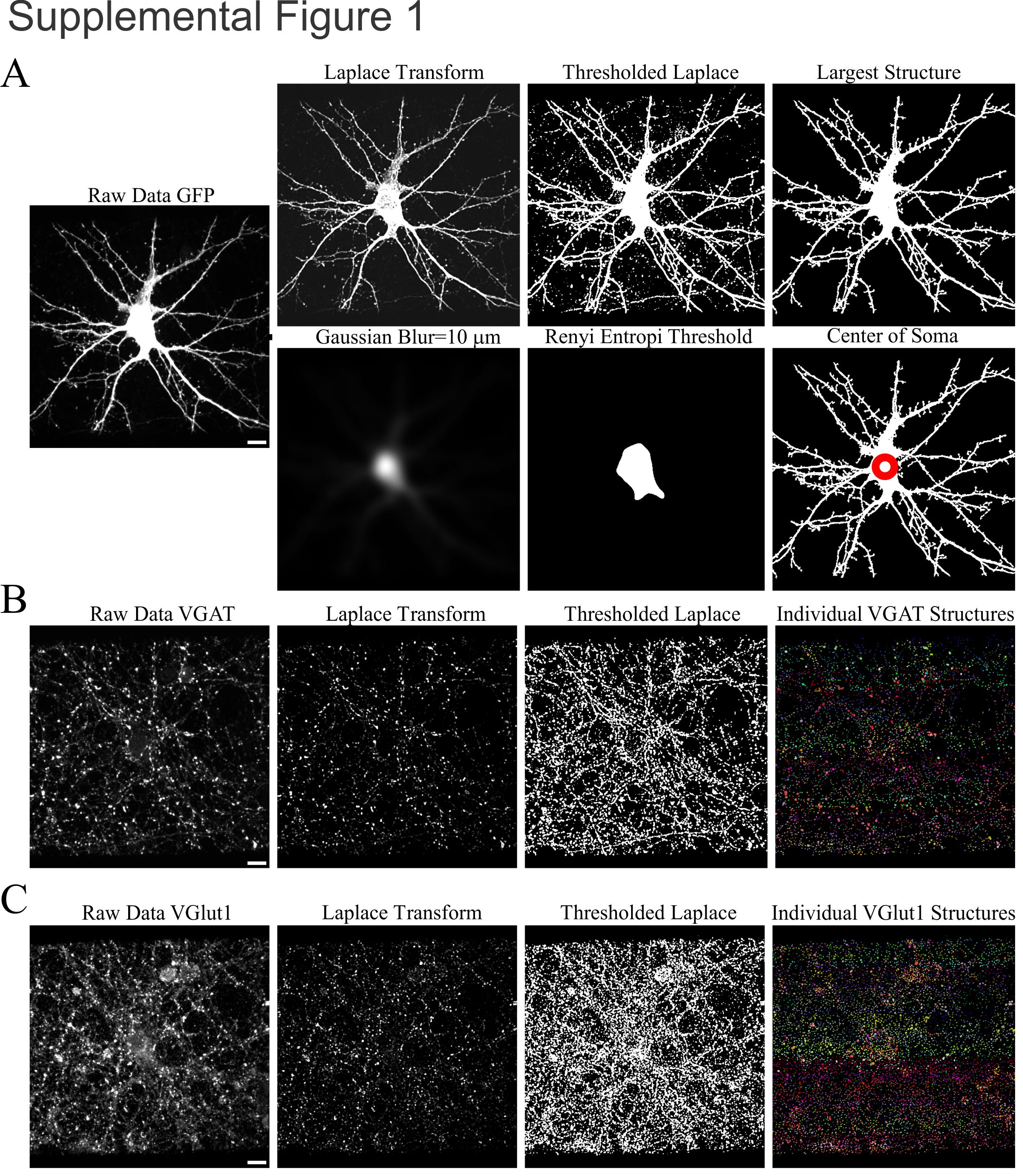

Supplement: FIGURE S1 — An overview of the image analysis routine used to analyze and segment images. (A: Top) The routine used to segment the GFP labeled neuron from background signal. The raw data is convolved with a Laplacian of Gaussian (LOG) kernel (left). The LOG convolution is thresholded (middle) and the thresholded data is treated with a connectedness filter to remove all but the largest connected object. (A: Bottom) The procedure used to find the center of the cell soma. The raw data is convolved with a high-radius Gaussian filter (left). A Renyi entropy threshold is applied to the Gaussian image to identify the soma (middle). The centroid of the soma is calculated and used as the center (right). (B,C) The procedure used to segment synaptic puncta. The raw data is convolved with a LOG (left). The LOG is thresholded (middle) and each individual feature is assigned a unique identifier (right). The color code in right hand images utilizes a 3-3-2 RGB lookup table where each individual feature is filled with a different color. [file Image_1.JPEG]

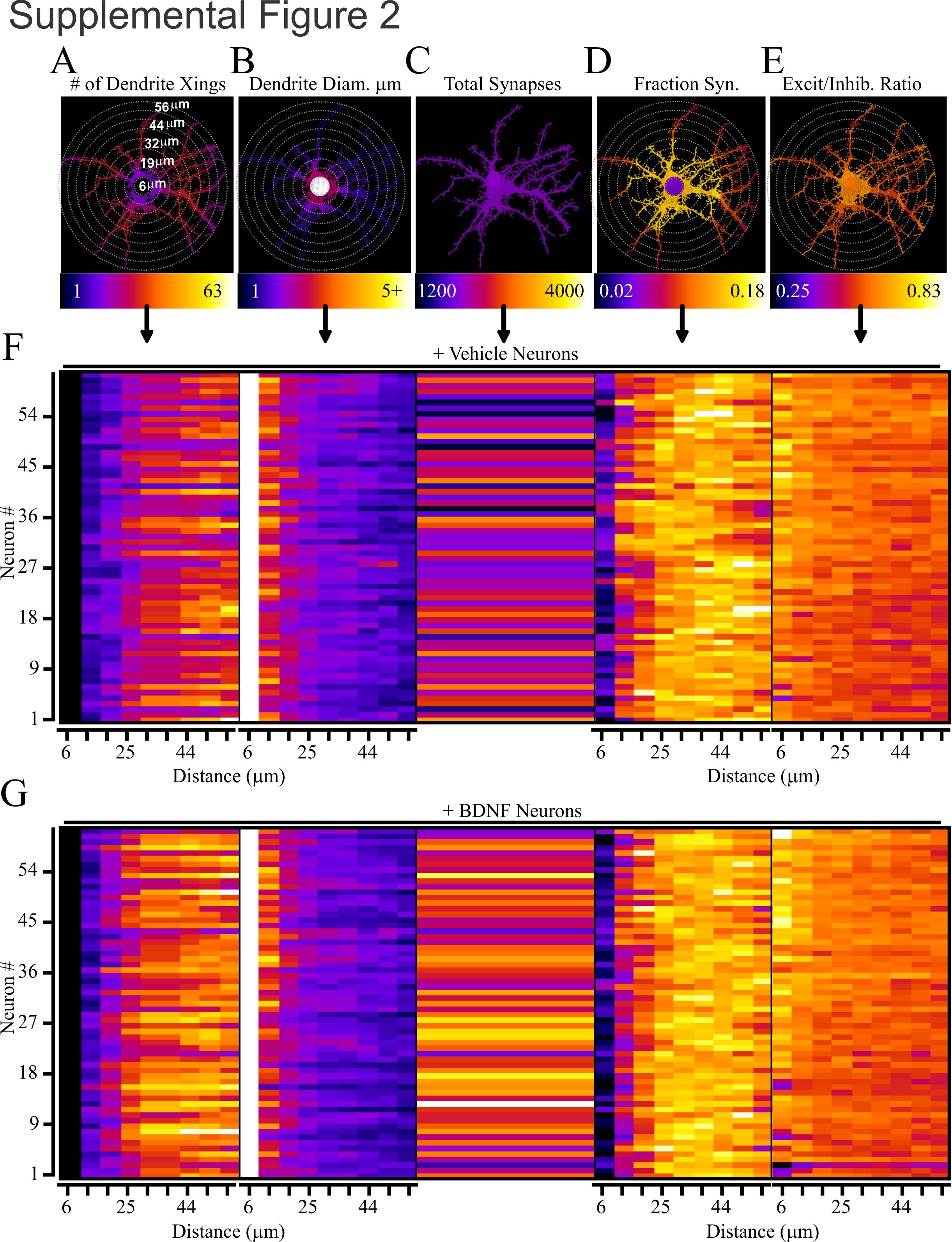

Supplement: FIGURE S2 — Creating numerical representations of individual neurons to use in computer simulations that reflect the distribution of dendrites and synapses calculated with the image analysis routine. (A–E) Heat maps projected onto a representative neuron depicting the approach used to constrain computational models of AP generation with morphological parameters. The concentric dashed lines mark individual 6.25 μm annuli. The lookup tables (LUTs) below each image correspond to both the neuron image and the heat maps below. (A) Each model neuron contains 6.25 μm dendrite segments equal in number to the number of dendrite crossings determined via Sholl analysis. The first domain for each neuron is the somatic compartment. (B) The diameter of each dendrite section was determined by calculating the total GFP volume within each Sholl radius, dividing this value by the number dendrite sections for that Sholl radius and solving for the diameter of the resulting cylinder. The LUT is truncated at 5 μm to preserve subtle differences in dendrite diameter throughout the arbor. (C) The total number of excitatory and inhibitory synapses is summed across the entire arbor. (D) Individual active synapses are assigned a location based on the relative distribution of synapses throughout the dendritic arbor. This strategy was necessary in order to randomize the process of stimulating synapses when the strength of stimulation was less than 100%. The LUT represents the probability of an active synapse being placed at a specific distance from the soma. (E) Individual synapses were assigned an excitatory (0 mV) or inhibitory (-70 mV) reversal potential according to the ratio of VGlut1 to VGAT punctae within each Sholl radius. The conductance of each synapse was randomly assigned using the normalized frequency distribution of synaptic puncta intensities which is similar to the procedure used by Katz et al. (2009). (F,G) Heat maps representing the combination of sections, section diameters, synapses, syn [file Image_2.JPEG]

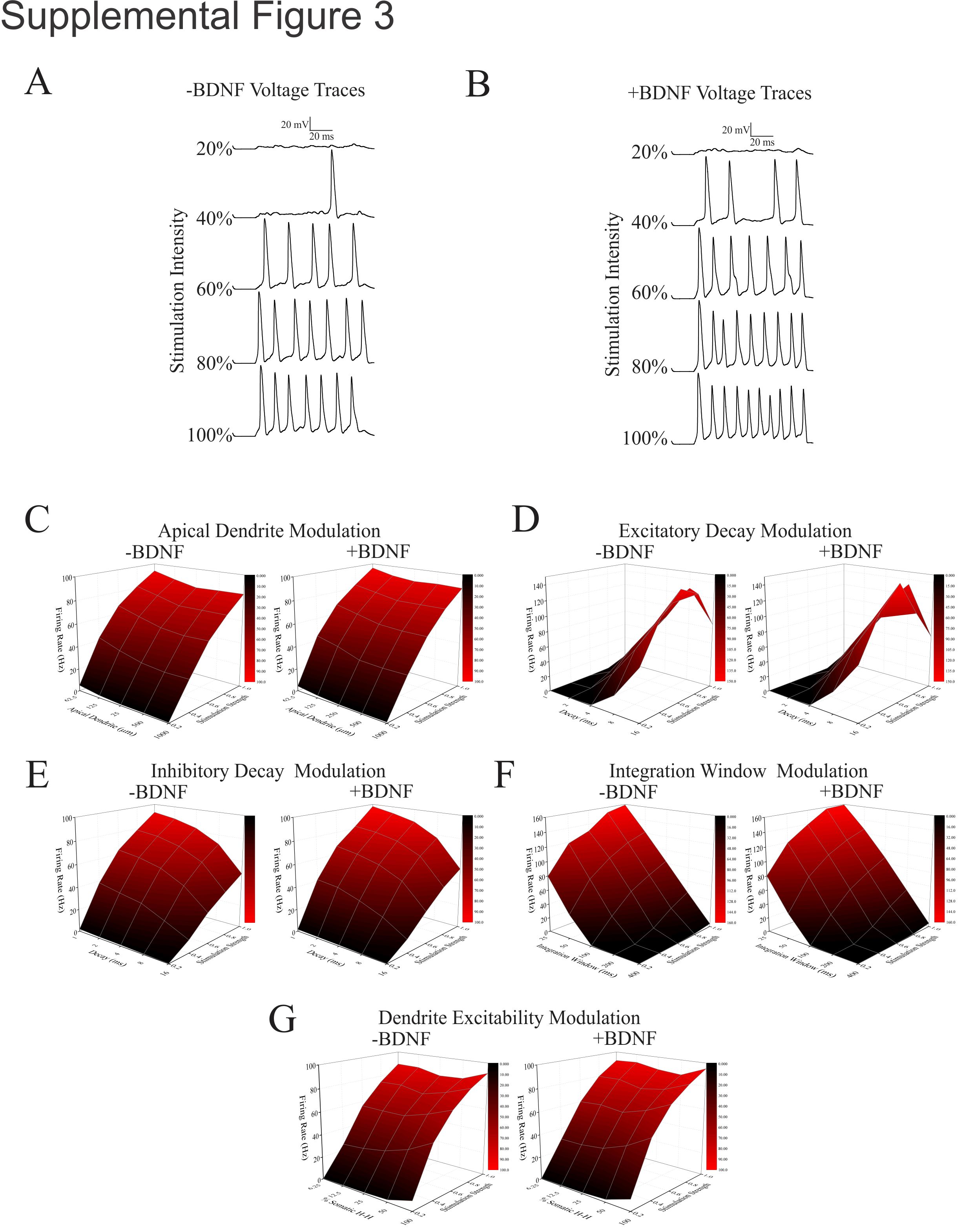

Supplement: FIGURE S3 — Computational neuron model construction based on morphometric data and model action potential generation rates with a broad range of input parameter values. (A,B) Representative voltage traces for individual models representing a -BDNF neuron (A) and a +BDNF neuron (B). The voltage traces are representative plots from the cable models generated when each of the parameter values were set to their mid-value. The percentage on the left side of each plot refers to the percentage of synapses that were randomly activated during the simulation. (C–G) Contour plots showing the average firing rate for the 63 -BDNF model neurons and 63 +BDNF model neurons as the indicated parameter was varied over a range of values. [file Image_3.JPEG]

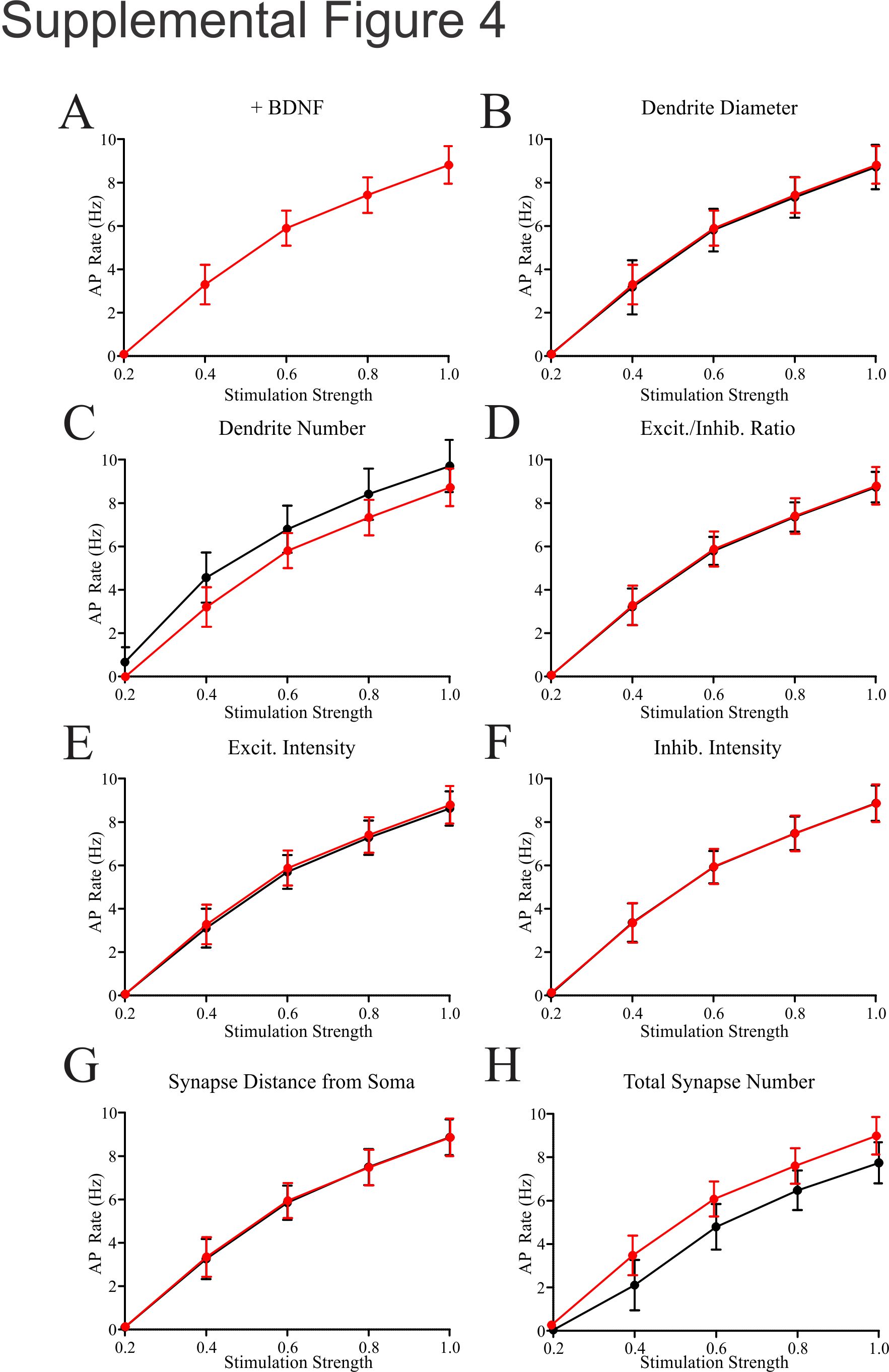

Supplement: FIGURE S4 — Substituting the average morphological parameter values from the -BDNF model neurons into the +BDNF model neurons does not dramatically alter the input–output relationship of the simulations. (A–H) The average AP rates and standard deviations for the +BDNF model neurons after substituting each of the -BDNF morphological parameter values across all stimulation strengths. The rates for unsubstituted +BDNF model neurons are shown in (A) and are reproduced in red for each substitution for comparison (B–H). [file Image_4.JPEG]
