# Supplementary material for: Computer Simulations Support a Morphological Contribution to BDNF Enhancement of Action Potential Generation
Source: Front Cell Neurosci. 2016 Sep 14;10:209. doi: 10.3389/fncel.2016.00209 (PMC5021759; doi:10.3389/fncel.2016.00209)
Supplement: TABLE S1 — A list of the parameter values used for aspects of the computer simulations that were not based on empirical morphological data. [file Table_1.PDF]

Supplemental Table 1

Location	Parameter	Value	Published Value	Reference
All Compartments:	R_i	105 Ω cm	150, 105, 80 Ω cm	1-3
	C_m	1 μF/cm^2	0.75, 1, 0.75 μF/cm^2	1-3
	g_{leak}	0.001	negligible effect, not reported , 0.033	1-3
	E_{Na}	50 mV	50, 60, 50 mV	1-3
	E_K	-87 mV	-90, -87, -90 mV	1-3
	E_{leak}	-70 mV	-70, -70, -70 mV	1-3
Axon Hodgkin-Huxley:	g_{Na}	360 mS/cm^2	300, 1000, 300 mS/cm^2	1-3
	g_K	10.8 mS/cm^2	20, 10, 20 mS/cm^2	1-3
Soma Hodgkin-Huxley:	g_{Na}	120 mS/cm^2	0.2, 100, 0.2 mS/cm^2	1-3
	g_K	3.6 mS/cm^2	2, 1, 2 mS/cm^2	1-3
Dendrites Hodgkin-Huxley:	g_{Na}	0.75-120 mS/cm^2	0.2, 10, 0.2 mS/cm^2	1-3
	g_K	0.225-3.6 mS/cm^2	0.001, 0.3, 0 mS/cm^2	1-3
Excitatory Synapse:	$t_{rise,fall}$	0.2, 1-16 ms	0, 0.5; 0.2, 5; 0.2,1.7; 0.05, 0.5 AMPA or 2.1, 18.8 NMDA ms	2,4-6
	$E_{excit.\ syn}$	0 mV	not reported, 0, 0, 0 mV	2,4-6
	g_{peak}	0.25-4 nS	1.5, 0.3-0.99, not reported, 1.5 AMPA or 3 NMDA nS	2,4-6
Inhibitory Synapse:	$t_{rise,fall}$	0.2, 1-16 ms	not modeled; not modeled; 0.75, 10; 0.5, 2 or 0.5, 100 ms	2,4-6
	$E_{inhib.\ syn}$	-70 mV	not modeled, not modeled, -75, -60 to -80 mV	2,4-6
	g_{peak}	0.25-4 nS	not modeled, not modeled, not reported, not reported, 0.5 nS	2,4-7

1. Mainen and Sejnowski, 1996
2. Nevian et al,., 2007
3. van Elburg et al., 2010
4. Katz et al., 2009
5. Komendantov et al., 2009
6. Jadi et al., 2012
7. Bush and Sejnowski, 1994
