# Supplementary material for: Computer Simulations Support a Morphological Contribution to BDNF Enhancement of Action Potential Generation
Source: Front Cell Neurosci. 2016 Sep 14;10:209. doi: 10.3389/fncel.2016.00209 (PMC5021759; doi:10.3389/fncel.2016.00209)
Supplement: TABLE S2 — The range of parameter values used to evaluate the robustness of the computer simulations. [file Table_2.PDF]

Supplemental Table 2

**Varied Parameter**	**Mid-Value**	**Range of Values**
Apical Dendrite Length:	250 μm	62.5 μm, 125 μm, 250 μm, 500 μm, 1000 μm
Excitatory Synapse Decay:	1-16 ms	1 ms, 2 ms, 4 ms, 8 ms, 16 ms
Inhibitory Synapse Decay:	1-16 ms	1 ms, 2 ms, 4 ms, 8 ms, 16 ms
Integration Window:	100 ms	25 ms, 50 ms, 100 ms, 200 ms, 400 ms
Dendrites Hodgkin-Huxley:	30 mS/cm^2	7.5,15,30,60,120
	0.9 mS/cm^2	0.225,0.45,0.9,1.8,3.6
Stimulation Strength:	0.6	0.2,0.4,0.6,0.8,1.0
