# Supplementary material for: Computer Simulations Support a Morphological Contribution to BDNF Enhancement of Action Potential Generation
Source: Front Cell Neurosci. 2016 Sep 14;10:209. doi: 10.3389/fncel.2016.00209 (PMC5021759; doi:10.3389/fncel.2016.00209)
Supplement: DATA SHEET 1 — The FIJI/ImageJ macro code used to perform automated image analysis of dendrites and synapses. [file Data_Sheet_1.PDF]

```
/*
 * This code assumes that you have a folder of images stacks grouped in three, where image 1 is
 a cell fill and images 2 and 3 are punctae that can be mapped to the cell fill. This code
 requires FIJI and the plugins FeatureJ by Eric Meijering and the 3D Suite by Thomas Boudier.
 In order for the code to run, this file ("Synapse and DendriteAnalysis.ijm") must be saved in
 your FIJI "macros" folder. In addition the files "Segment_Strucures.ijm",
 "Distance_Transform_Soma.ijm", "Analyze_Skeleton.ijm", "Distance_Analysis.ijm", and
 "Threshold.ijm" must be in the FIJI "macros" folder as well.
 *
 *
 * In general, aspects of this code were optimzied for our microscope setup (ie., file naming,
 pixel size, etc). However, the code can be easily modified to match any file format. So if you
 are interested in using the code, please contact me (Nick Galati) at nick.galati80@gmail.com
 or go through GitHub at https://github.com/nickgalati80. I'd be glad to help get the code
 running on any image format and to help you sort through the data.
 *
 * Thanks!
 */



/*
 *  Synapse_and_Dendrite_Analysis.ijm
 *
 *  Save the following code as "Synapse_and_Dendrite_Analysis.ijm" to your macros folder.
 *  Open this code in the Script editor and press run to start the macro.
 */
setBatchMode(true);
setForegroundColor(255,255,255);
run("Set Measurements...", "area mean standard modal min centroid center perimeter bounding fit
shape feret's integrated median skewness kurtosis area_fraction stack display redirect=None
decimal=3");
file=File.openDialog("Choose a Neuron File");
open(file);
rawFile=File.getName(file);
rawFile2=replace(rawFile, "_w1488", "_");
rawFile3=replace(rawFile2, ".TIF", "");
file2=replace(file, "_w1488", "_w2568");
file3=replace(file, "_w1488", "_w3647");
directory=File.getParent(file)+"\\"+rawFile3;
print(directory);
open(file2);
open(file3);

selectImage(1);
rename("Neuron");
selectImage(2);
rename("VGAT");
selectImage(3);
rename("VGlut1");

//Cell Fill Segmentation
selectImage("Neuron");
runMacro("Segment_Structures.ijm", "Neuron"+"\t"+"Neuron");
//Puncta 1 Segmentation
selectImage("VGAT");
runMacro("Segment_Structures.ijm", "Puncta"+"\t"+"VGAT");
//Puncta 2 Segmentation
selectImage("VGlut1");
runMacro("Segment_Structures.ijm", "Puncta"+"\t"+"VGlut1");
//
selectImage("Segmented_Soma");
runMacro("Distance_Transform_Soma.ijm");

selectImage("Segmented_Neuron");
runMacro("Analyze_Skeleton.ijm");

runMacro("Distance_Analysis.ijm",directory);
setBatchMode("exit and display");
```

```
/*
 *  Segment_Structures.ijm
 *
 *  Save the following code as "Segment_Structures.ijm" to your macros folder
 */
argument=split(getArgument,"\t");
sliceStart=1;
sliceEnd=nSlices;
sliceRange=toString(sliceStart)+"-"+toString(sliceEnd);
run("FeatureJ Laplacian", "compute smoothing=1.0");
rename("Mean_Lap");
selectImage("Mean_Lap");
run("Invert", "stack");
run("Z Project...", "start=1 stop=&sliceEnd projection=[Max Intensity]");
getStatistics(area,mean,min,max,std,histo);
run("Measure");
close();
mode=getResult("Mode");
selectWindow("Results");
run("Close");
selectImage("Mean_Lap");
if(argument[0]=="Puncta"){
    setMinAndMax(mean, mean+(5*std));
}
if(argument[0]=="Neuron"){
    setMinAndMax(mode+(0.1*std), mode+(0.1*std));
}
run("8-bit");
if(argument[0]=="Neuron"){
    setMinAndMax(0,0);
    run("Apply LUT", "stack");
    run("Find Connected Regions", "allow_diagonal display_one_image display_results
    regions_for_values_over=100 minimum_number_of_points=1 stop_after=-1");
    rename("Segmented_Neuron");
    selectImage("Mean_Lap");
    close();
    sizeArray=newArray(nResults);
    for(a=0; a<nResults; a++){
        sizeArray[a]=getResult("Points In Region", a);
    }
    Array.getStatistics(sizeArray, min,neuronmax,mean,std);
    for(a=0; a<nResults; a++){
        value=sizeArray[a];
        if(value==neuronmax){
            thresholdMin=a+1;
            thresholdMax=a+1;
        }
    }
    selectImage("Segmented_Neuron");
    for(a=0; a<nSlices; a++){
        setSlice(a+1);
        setThreshold(thresholdMin, thresholdMax);
        run("Create Selection");
        if(selectionType>=0){
            run("Fill", "slice");
            run("Clear Outside", "slice");
        }
        if(selectionType==-1){
            run("Select All");
            run("Clear", "slice");
        }
        run("Select None");
        resetThreshold();
    }
    selectWindow("Results");
    run("Close");
```

```
    runMacro("Threshold.ijm");
    run("Grays");
}
///
if(argument[0]=="Neuron"){
    selectImage("Neuron");
    run("Duplicate...", "title=Soma duplicate range=&sliceRange");
    run("Enhance Contrast...", "saturated=0.4 use");
    run("8-bit");
    run("Gaussian Blur...", "sigma=10 stack");
    run("Z Project...", "start=1 stop=&sliceEnd projection=[Sum Slices]");
    run("8-bit");
    setAutoThreshold("RenyiEntropy dark");
    getThreshold(minSoma,maxSoma);
    close();
    selectImage("Soma");
    setThreshold(minSoma,maxSoma);
    for(a=0; a<nSlices; a++){
        selectImage("Soma");
        setSlice(a+1);
        run("Create Selection");
        if(selectionType>=0){
            run("Enlarge...", "enlarge=-2.5");
            run("Fill", "slice");
            run("Clear Outside", "slice");
        }
        if(selectionType==-1){
            run("Select All");
            run("Clear", "slice");
        }
        run("Select None");
    }
    run("Find Connected Regions", "allow_diagonal display_one_image display_results
    regions_for_values_over=100 minimum_number_of_points=1 stop_after=-1");
    rename("Segmented_Soma");
    selectImage("Soma");
    close();
    sizeArray=newArray(nResults);
    for(a=0; a<nResults; a++){
        sizeArray[a]=getResult("Points In Region", a);
    }
    Array.getStatistics(sizeArray, min,neuronmax,mean,std);
    for(a=0; a<nResults; a++){
        value=sizeArray[a];
        if(value==neuronmax){
            thresholdMin=a+1;
            thresholdMax=a+1;
        }
    }
    selectImage("Segmented_Soma");
    for(a=0; a<nSlices; a++){
        setSlice(a+1);
        setThreshold(thresholdMin, thresholdMax);
        run("Create Selection");
        if(selectionType>=0){
            run("Fill", "slice");
            run("Clear Outside", "slice");
        }
        if(selectionType==-1){
            run("Select All");
            run("Clear", "slice");
        }
        run("Select None");
        resetThreshold();
    }
    selectWindow("Results");
    run("Close");
    runMacro("Threshold.ijm");
    run("Grays");
```

```
    run("8-bit");

}
if(argument[0]=="Puncta"){
    run("3D Fast Filters","filter=MaximumLocal radius_x_pix=2.0 radius_y_pix=2.0 
    radius_z_pix=1.0 Nb_cpus=4");
    runMacro("Threshold.ijm");
    run("3D Spot Segmentation", "seeds_threshold=0 local_background=1 radius_0=3 radius_1=3 
    radius_2=4 weigth=0.50 radius_max=10 sd_value=.95 local_threshold=[Constant] 
    seg_spot=Classical watershed volume_min=1 volume_max=5 seeds=3D_MaximumLocal spots=Mean_Lap 
    radius_for_seeds=2 output=[Label Image]");
    runMacro("Threshold.ijm");
    rename("Segmented_"+argument[1]);
    selectImage("3D_MaximumLocal");
    close();
    selectImage("Mean_Lap");
    close();
}



/*
 *  Distance_Transform_Soma.ijm
 *
 *  Save the following code as "Distance_Transform_Soma.ijm" to your macros folder
 */
run("3D Manager");
Ext.Manager3D_AddImage();
Ext.Manager3D_MassCenter3D(0, cmx,cmy,cmz);
Ext.Manager3D_Reset();
run("Replace value", "pattern=0 replacement=1");
setSlice(cmz);
makePoint(cmx,cmy);
setForegroundColor(0,0,0);
run("Draw", "slice");
setForegroundColor(255,255,255);
run("Exact Euclidean Distance Transform (3D)");
rename("Distance_Map");
for(a=0; a<nImages; a++){
    selectImage(a+1);
    run("Specify...", "width=450 height=450 x=&cmx y=&cmy slice=&cmz oval centered");
    run("Clear Outside", "stack");
    run("Select None");
}
selectImage("Segmented_Soma");
run("Replace value", "pattern=1 replacement=0");





/*
 *  Analyze_Skeleton.ijm
 *
 *  Save the following code as "Analyze_Skeleton.ijm" to your macros folder
 */
selectImage("Segmented_Neuron");
sliceStart=1;
sliceEnd=nSlices;
sliceRange=toString(sliceStart)+"-"+toString(sliceEnd);
run("Duplicate...", "title=DistanceSkeleton duplicate range=&sliceRange");
distanceSkeleton=getImageID();
run("Duplicate...", "title=Skeleton duplicate range=&sliceRange");
skeleton=getImageID();
selectWindow("Skeleton");
run("Skeletonize (2D/3D)");
selectWindow("Distance");
run("Replace value", "pattern=0 replacement=1");
run("Replace value", "pattern=255 replacement=3");
selectWindow("Skeleton");
```

```
run("Replace value", "pattern=255 replacement=3");
imageCalculator("Subtract create stack", "Distance","Skeleton");
rename("DistanceMap");
run("3D Distance Map", "threshold=0");
rename("Diameter");
for(a=0; a<nSlices; a++){
    selectImage("Segmented_Neuron");
    setSlice(a+1);
    setThreshold(1,255);
    run("Create Selection");
    if(selectionType>=0){
        roiManager("Add");
        selectImage("Diameter");
        setSlice(a+1);
        roiManager("Select",0);
        run("Clear Outside", "slice");
        roiManager("Select", 0);
        roiManager("Delete");

    }
    if(selectionType==-1){
        selectImage("Diameter");
        setSlice(a+1);
        run("Select All");
        run("Clear", "slice");

    }
    run("Select None");
}
selectImage("Segmented_Neuron");
run("Select None");
resetThreshold();
selectImage("Distance");
close();
selectImage("DistanceMap");
close();
selectImage("Skeleton");
rename("Segmented_Skeleton");
runMacro("Threshold.ijm");
selectImage("Diameter");
run("Gaussian Blur...", "sigma=10 stack");





/*
 *  Distance_Analysis.ijm
 *
 *  Save the following code as "Distance_Analysis.ijm" to your macros folder
 */

argument=getArgument();
run("3D Manager");
nameArray=newArray("Neuron", "Soma", "Skeleton", "VGAT_TFX", "VGAT_NonTFX", "VGlut1_TFX",
"VGlut1_NonTFX");
Ext.Manager3D_MonoSelect();
//
selectImage("Segmented_Neuron");
Ext.Manager3D_AddImage();
Ext.Manager3D_Select(0);
Ext.Manager3D_Rename("Neuron Object");
Ext.Manager3D_Save(argument+"Neuron"+".zip");
Ext.Manager3D_Measure3D(0, "Vol", totalNeuronVolume);
Ext.Manager3D_Reset();
//
selectImage("Segmented_Skeleton");
Ext.Manager3D_AddImage();
Ext.Manager3D_Select(0);
```

```
Ext.Manager3D_Rename("Skeleton Object");
Ext.Manager3D_Save(argument+"Skeleton"+".zip");
Ext.Manager3D_Measure3D(0, "Vol", totalNeuronLength);
selectImage("Diameter");
Ext.Manager3D_Quantif3D(0, "Mean", averageNeuronDiameter);
Ext.Manager3D_Reset();

//
selectImage("Segmented_VGAT");
Ext.Manager3D_AddImage();
Ext.Manager3D_Select(0);
Ext.Manager3D_Rename("VGAT Object");
Ext.Manager3D_Save(argument+"VGAT_Total"+".zip");
Ext.Manager3D_Reset();
//
selectImage("Segmented_VGlut1");
Ext.Manager3D_AddImage();
Ext.Manager3D_Select(0);
Ext.Manager3D_Rename("VGlut1 Object");
Ext.Manager3D_Save(argument+"VGlut1_Total"+".zip");
Ext.Manager3D_Reset();
//////////////////////////////////////////////////////////////////////////////////////////////
selectImage("Segmented_VGAT");
Ext.Manager3D_Segment(1,255);
close;
Ext.Manager3D_AddImage();
close;
Ext.Manager3D_Count(objects);
Ext.Manager3D_MultiSelect();
selectImage("Segmented_Neuron");
for(a=0; a<objects; a++){
    Ext.Manager3D_Quantif3D(a, "Max", value);
    if(value>0){
        Ext.Manager3D_Select(a);
    }
}
Ext.Manager3D_Save(argument+"VGAT_TFX"+".zip");
newImage("VGAT_TFX", "8-bit black", 512, 512, 13);
Ext.Manager3D_FillStack(255,255,255);
Ext.Manager3D_Delete();
Ext.Manager3D_MultiSelect();
Ext.Manager3D_SelectAll();
Ext.Manager3D_Save(argument+"VGAT_NonTFX"+".zip");
newImage("VGAT_NonTFX", "8-bit black", 512, 512, 13);
Ext.Manager3D_FillStack(255,255,255);
Ext.Manager3D_DeselectAll();
Ext.Manager3D_Reset();
/////////////////////////////////////////////////////////////////////////////////////////////////
/////////////
selectImage("Segmented_VGlut1");
Ext.Manager3D_Segment(1,255);
close;
Ext.Manager3D_AddImage();
close;
Ext.Manager3D_Count(objects);
Ext.Manager3D_MultiSelect();
selectImage("Segmented_Neuron");
for(a=0; a<objects; a++){
    Ext.Manager3D_Quantif3D(a, "Max", value);
    if(value>0){
        Ext.Manager3D_Select(a);
    }
}
Ext.Manager3D_Save(argument+"VGlut1_TFX"+".zip");
newImage("VGlut1_TFX", "8-bit black", 512, 512, 13);
Ext.Manager3D_FillStack(255,255,255);
Ext.Manager3D_Delete();
Ext.Manager3D_MultiSelect();
Ext.Manager3D_SelectAll();
```

```
Ext.Manager3D_Save(argument+"VGlut1_NonTFX"+".zip");
newImage("VGlut1_NonTFX", "8-bit black", 512, 512, 13);
Ext.Manager3D_FillStack(255,255,255);
Ext.Manager3D_DeselectAll();
Ext.Manager3D_Reset();
//////////////////////////////////////////////////////////////

/////////////////////////////////////////////////////////////////////////////////////////////
newImage("Cross_Synapses", "8-bit black", 512, 512, 13);
titleArray=newArray("VGAT", "VGAT","VGlut1","VGlut1");
secondTitleArray=newArray("VGlut1_TFX", "VGlut1_NonTFX", "VGAT_TFX", "VGAT_NonTFX");
binArray=newArray("binOne","binTwo","binThree","binFour","binFive","binSix","binSeven","binEight"
,"binNine");
selectionArray=newArray(36);
totalObjectsArray=newArray(4);
counter=0;
for(a=0; a<4; a++){
    zipFile=argument+nameArray[a+3]+".zip";
    Ext.Manager3D_Load(zipFile);
    Ext.Manager3D_Count(objects);
    totalObjectsArray[a]=objects;
    selectImage(secondTitleArray[a]);
    Ext.Manager3D_AddImage();
    masterArray=newArray(objects*8);
    bin1Array=newArray();
    bin2Array=newArray();
    bin3Array=newArray();
    bin4Array=newArray();
    bin5Array=newArray();
    bin6Array=newArray();
    bin7Array=newArray();
    bin8Array=newArray();
    bin9Array=newArray();
    newImage(nameArray[a+3]+"_Total", "32-bit black",8,objects,1);
    for(b=0; b<objects; b++){
        tempArray=newArray(8);
        selectImage(8);
        Ext.Manager3D_Quantif3D(b, "Mean", somadistance);
        if(somadistance<=25){bin=1;}
        if(somadistance>25 && somadistance<=50){bin=2;}
        if(somadistance>50 && somadistance<=75){bin=3;}
        if(somadistance>75 && somadistance<=100){bin=4;}
        if(somadistance>100 && somadistance<=125){bin=5;}
        if(somadistance>125 && somadistance<=150){bin=6;}
        if(somadistance>150 && somadistance<=175){bin=7;}
        if(somadistance>175 && somadistance<=200){bin=8;}
        if(somadistance>200){bin=9;}
        selectImage(titleArray[a]);
        Ext.Manager3D_Quantif3D(b, "Max", samemax);
        Ext.Manager3D_Closest(b, "cc", sameclosest);
        Ext.Manager3D_Dist2(b,sameclosest, "c1b2", closest);
        selectImage(10);
        Ext.Manager3D_Quantif3D(b, "Max", dendritediameter);
        Ext.Manager3D_Dist2(b, objects, "c1b2", otherdistance);
        if(otherdistance<1){
            selectImage("Cross_Synapses");
            Ext.Manager3D_Select(b);
            fillColor=(a+10)*10;
            Ext.Manager3D_FillStack(fillColor,fillColor,fillColor);
            cross=1;
            notcross=0;
        }
        if(otherdistance>=1){
            cross=0;
            notcross=1;
        }
        index=b*8;

        masterArray[index+0]=bin;masterArray[index+1]=somadistance;masterArray[index+2]=samemax;m
```

```
        asterArray[index+3]=closest;masterArray[index+4]=dendritediameter;masterArray[index+5]=ot
        herdistance;masterArray[index+6]=cross;masterArray[index+7]=notcross;
        selectImage(nameArray[a+3]+"_Total");

        setPixel(0,b,bin);setPixel(1,b,somadistance);setPixel(2,b,samemax);setPixel(3,b,closest);
        setPixel(4,b,dendritediameter);setPixel(5,b,otherdistance);setPixel(6,b,cross);setPixel(7
        ,b,notcross);
    }
    for(b=0; b<objects; b++){
        showStatus("HI"+a);
        showProgress(b/objects);
        index=b*8;
        bin=masterArray[index];
        slice=Array.slice(masterArray,index, index+8);
        if(bin==1){bin1Array=Array.concat(bin1Array, slice);}
        if(bin==2){bin2Array=Array.concat(bin2Array, slice);}
        if(bin==3){bin3Array=Array.concat(bin3Array, slice);}
        if(bin==4){bin4Array=Array.concat(bin4Array, slice);}
        if(bin==5){bin5Array=Array.concat(bin5Array, slice);}
        if(bin==6){bin6Array=Array.concat(bin6Array, slice);}
        if(bin==7){bin7Array=Array.concat(bin7Array, slice);}
        if(bin==8){bin8Array=Array.concat(bin8Array, slice);}
        if(bin==9){bin9Array=Array.concat(bin9Array, slice);}
    }
    length1=bin1Array.length/8;
    length2=bin2Array.length/8;
    length3=bin3Array.length/8;
    length4=bin4Array.length/8;
    length5=bin5Array.length/8;
    length6=bin6Array.length/8;
    length7=bin7Array.length/8;
    length8=bin8Array.length/8;
    length9=bin9Array.length/8;
    heightArray=newArray(9);

    heightArray[0]=length1;heightArray[1]=length2;heightArray[2]=length3;heightArray[3]=length4;h
    eightArray[4]=length5;heightArray[5]=length6;heightArray[6]=length7;heightArray[7]=length8;he
    ightArray[8]=length9;
    if(length1==0){
        heightArray[0]=1;
        bin1Array=newArray(8);

        bin1Array[0]=1;bin1Array[1]=0;bin1Array[2]=0;bin1Array[3]=0;bin1Array[4]=0;bin1Array[5]=0
        ;bin1Array[6]=0;bin1Array[7]=0;
    }

    for(b=0; b<9; b++){
        height=heightArray[b];

        binArray=newArray("binOne","binTwo","binThree","binFour","binFive","binSix","binSeven","b
        inEight","binNine");
        newImage(nameArray[a+3]+"_"+binArray[b], "32-bit black",8,height,1);
    }
    for(b=0; b<9; b++){
        selectImage(nameArray[a+3]+"_"+binArray[b]);

        if(b==0)array=bin1Array;if(b==1)array=bin2Array;if(b==2)array=bin3Array;if(b==3)array=bin
        4Array;if(b==4)array=bin5Array;if(b==5)array=bin6Array;if(b==6)array=bin7Array;if(b==7)ar
        ray=bin8Array;if(b==8)array=bin9Array;
        selectionArray[counter]=array.length/8;
        counter++;
        for(c=0; c<array.length/8; c++){
            for(d=0; d<8; d++){
                index=c*8;
                setPixel(d,c,array[index+d]);
            }
        }
    }
Ext.Manager3D_Reset();
```

```
}
run("Images to Stack", "method=[Copy (top-left)] name=Binned_Results title=bin use");
run("Images to Stack", "method=[Copy (top-left)] name=Total_Results title=_Total use");
/////////////////////////////////////////
selectImage("Binned_Results");
averageBinnedResults=newArray(288);
counter=0;
counterTwo=0;
for(b=0; b<36; b++){
    slice=b+1;
    setSlice(slice);
    height=selectionArray[counter];
    counter++;
    for(c=0; c<8; c++){
        run("Specify...", "width=1 height=&height x=&c y=0 slice=&slice");
        getStatistics(area,mean,min,max,std,histo);
        if(c<=5){value=mean;}
        else{value=round(mean*area);}
        averageBinnedResults[counterTwo]=value;
        run("Select None");
        counterTwo++;

    }
}
selectImage("Total_Results");
averageTotalResults=newArray(32);
counter=0;
for(b=0; b<4; b++){
    slice=b+1;
    setSlice(slice);
    height=totalObjectsArray[b];
    for(c=0; c<8; c++){
        run("Specify...", "width=1 height=&height x=&c y=0 slice=&slice");
        getStatistics(area,mean,min,max,std,histo);
        if(c<=5){value=mean;}
        else{value=round(mean*area);}
        averageTotalResults[counter]=value;
        run("Select None");
        counter++;
    }
}
////
selectImage("Cross_Synapses");
Ext.Manager3D_AddImage();
Ext.Manager3D_Select(0);
Ext.Manager3D_Save(argument+"CrossSyn_VGAT_TFX.zip");
Ext.Manager3D_Select(1);
Ext.Manager3D_Save(argument+"CrossSyn_VGAT_NonTFX.zip");
Ext.Manager3D_Select(2);
Ext.Manager3D_Save(argument+"CrossSyn_VGlut1_TFX.zip");
Ext.Manager3D_Select(3);
Ext.Manager3D_Save(argument+"CrossSyn_VGlut1_NonTFX.zip");
Ext.Manager3D_Reset();
//
selectImage("Distance_Map");
run("Z Project...", "start=1 stop=13 projection=[Min Intensity]");
rename("CenterPoint");
setThreshold(0, 0);
run("Create Selection");
roiManager("Add");
roiManager("Split");
selectImage("CenterPoint");
close();
selectImage("Segmented_Skeleton");
run("Z Project...", "start=1 stop=13 projection=[Max Intensity]");
rename("Sholl");
roiManager("Select", 1);
run("Measure");
xCenter=getResult("BX", 0);
```

```
yCenter=getResult("BY", 0);
selectWindow("Results");
run("Close");
selectImage("Sholl");
makePoint(xCenter,yCenter);
run("ShollAnalysis ", "starting=1 ending=250 radius=25 radius=5 span=Median");
selectWindow("Sholl Analysis for Sholl");
Plot.getValues(distance, crossings);
Array.getStatistics(crossings,min,max,mean,std);
totalCrossings=floor(mean*crossings.length);
selectWindow("Sholl");
close();
selectWindow("Sholl Analysis for Sholl");
close();
roiManager("Deselect");
roiManager("Delete");
//
selectImage("Segmented_Neuron");
run("Replace value", "pattern=255 replacement=1");
selectImage("Segmented_Skeleton");
run("Replace value", "pattern=255 replacement=1");
imageCalculator("Multiply create 32-bit stack", "Segmented_Neuron","Distance_Map");
rename("Distance_Volume");
imageCalculator("Multiply create 32-bit stack", "Segmented_Skeleton","Distance_Map");
rename("Distance_Skeleton");
//
binnedVolumeArray=newArray(9);
for(a=0; a<9; a++){
    selectImage("Distance_Volume");
    Ext.Manager3D_Count(startObjects);
    lowThreshold=a*25+1;
    highThreshold=lowThreshold+25;
    Ext.Manager3D_Segment(lowThreshold, highThreshold);
    close();
    Ext.Manager3D_AddImage();
    close();
    Ext.Manager3D_Count(endObjects);
    if((endObjects-startObjects)>1){
        Ext.Manager3D_MultiSelect();
        Ext.Manager3D_SelectFor(startObjects, endObjects, 1);
        Ext.Manager3D_Merge();
        Ext.Manager3D_Count(measureObject);
        Ext.Manager3D_Measure3D(measureObject-1, "Vol", volume);
        Ext.Manager3D_DeselectAll();
    }
    Ext.Manager3D_Count(measureObject);
    Ext.Manager3D_Measure3D(measureObject-1, "Vol", volume);
    binnedVolumeArray[a]=volume;
}
binnedLengthArray=newArray(9);
binnedDiameterArray=newArray(9);
for(a=0; a<9; a++){
    selectImage("Distance_Skeleton");
    Ext.Manager3D_Count(startObjects);
    lowThreshold=a*25+1;
    highThreshold=lowThreshold+25;
    Ext.Manager3D_Segment(lowThreshold, highThreshold);
    close();
    Ext.Manager3D_AddImage();
    close();
    Ext.Manager3D_Count(endObjects);
    if((endObjects-startObjects)>1){
        Ext.Manager3D_MultiSelect();
        Ext.Manager3D_SelectFor(startObjects, endObjects, 1);
        Ext.Manager3D_Merge();
        Ext.Manager3D_DeselectAll();
    }
    Ext.Manager3D_Count(measureObject);
    Ext.Manager3D_Measure3D(measureObject-1, "Vol", volume);
```

```
    selectImage("Diameter");
    Ext.Manager3D_Quantif3D(measureObject-1, "Mean", diameter);
    binnedLengthArray[a]=volume;
    binnedDiameterArray[a]=diameter;
}
Ext.Manager3D_Save(argument+"BinnedMorhpology"+".zip");
Ext.Manager3D_DeselectAll();
Ext.Manager3D_MultiSelect();
Ext.Manager3D_SelectFor(9,17,1);
Ext.Manager3D_Merge();
selectImage("Diameter");
Ext.Manager3D_Quantif3D(9,"Mean",totalAverageDiameter);
Ext.Manager3D_Reset();
///
counter=0;
counterTwo=0;
for(a=0; a<4; a++){
    thirdTitleArray=newArray("VGAT_TFX", "VGAT_NonTFX", "VGlut1_TFX", "VGlut1_NonTFX");
    for(b=0; b<9; b++){
        setResult("Label", b, "Bin-"+averageBinnedResults[counter]);
        setResult(thirdTitleArray[a]+"_"+"Bin", b, averageBinnedResults[counter]);
        setResult(thirdTitleArray[a]+"_"+"Distance From Soma", b,
        averageBinnedResults[counter+1]);
        setResult(thirdTitleArray[a]+"_"+"Intensity", b, averageBinnedResults[counter+2]);
        setResult(thirdTitleArray[a]+"_"+"Closest Same", b, averageBinnedResults[counter+3]);
        setResult(thirdTitleArray[a]+"_"+"Dend. Diameter @ Synapse", b,
        averageBinnedResults[counter+4]);
        setResult(thirdTitleArray[a]+"_"+"Closest Other", b, averageBinnedResults[counter+5]);
        setResult(thirdTitleArray[a]+"_"+"Total Cross", b, averageBinnedResults[counter+6]);
        setResult(thirdTitleArray[a]+"_"+"Total Non Cross", b, averageBinnedResults[counter+7]);
        setResult(thirdTitleArray[a]+"_"+"Total", b,
        averageBinnedResults[counter+6]+averageBinnedResults[counter+7]);
        setResult(thirdTitleArray[a]+"_"+"Volume", b, binnedVolumeArray[b]);
        setResult(thirdTitleArray[a]+"_"+"Length", b, binnedLengthArray[b]);
        setResult(thirdTitleArray[a]+"_"+"Dendrite Diameter", b, binnedDiameterArray[b]);
        setResult(thirdTitleArray[a]+"_"+"Crossings", b, crossings[b]);
        setResult(thirdTitleArray[a]+"_"+"Cross Syn./Length", b,
        averageBinnedResults[counter+6]/binnedLengthArray[b]);
        setResult(thirdTitleArray[a]+"_"+"Cross Syn./Volume", b,
        averageBinnedResults[counter+6]/binnedVolumeArray[b]);
        setResult(thirdTitleArray[a]+"_"+"Non Cross Syn./Length", b,
        averageBinnedResults[counter+7]/binnedLengthArray[b]);
        setResult(thirdTitleArray[a]+"_"+"Non Cross Syn./Volume", b,
        averageBinnedResults[counter+7]/binnedVolumeArray[b]);
        setResult(thirdTitleArray[a]+"_"+"Total Syn./Length", b,
        (averageBinnedResults[counter+6]+averageBinnedResults[counter+7])/binnedLengthArray[b]);
        setResult(thirdTitleArray[a]+"_"+"Total Syn./Volume", b,
        (averageBinnedResults[counter+6]+averageBinnedResults[counter+7])/binnedVolumeArray[b]);

        counter=counter+8;

    }
    setResult("Label", 9, "Unbinned Average");
    setResult(thirdTitleArray[a]+"_"+"Distance From Soma", 9, averageTotalResults[counterTwo+1]);
    setResult(thirdTitleArray[a]+"_"+"Intensity", 9, averageTotalResults[counterTwo+2]);
    setResult(thirdTitleArray[a]+"_"+"Closest Same", 9, averageTotalResults[counterTwo+3]);
    setResult(thirdTitleArray[a]+"_"+"Dend. Diameter @ Synapse", 9,
    averageTotalResults[counterTwo+4]);
    setResult(thirdTitleArray[a]+"_"+"Closest Other", 9, averageTotalResults[counterTwo+5]);
    setResult(thirdTitleArray[a]+"_"+"Total Cross", 9, averageTotalResults[counterTwo+6]);
    setResult(thirdTitleArray[a]+"_"+"Total Non Cross", 9, averageTotalResults[counterTwo+7]);
    setResult(thirdTitleArray[a]+"_"+"Total", 9,
    averageTotalResults[counterTwo+6]+averageTotalResults[counterTwo+7]);
    setResult(thirdTitleArray[a]+"_"+"Volume", 9, totalNeuronVolume);
    setResult(thirdTitleArray[a]+"_"+"Length", 9, totalNeuronLength);
    setResult(thirdTitleArray[a]+"_"+"Dendrite Diameter", 9, totalAverageDiameter);
    setResult(thirdTitleArray[a]+"_"+"Crossings", 9, totalCrossings);
    setResult(thirdTitleArray[a]+"_"+"Cross Syn./Length", 9,
    averageTotalResults[counterTwo+6]/totalNeuronLength);
```

```
    setResult(thirdTitleArray[a]+"_"+"Cross Syn./Volume", 9,
    averageTotalResults[counterTwo+6]/totalNeuronVolume);
    setResult(thirdTitleArray[a]+"_"+"Non Cross Syn./Length", 9,
    averageTotalResults[counterTwo+7]/totalNeuronLength);
    setResult(thirdTitleArray[a]+"_"+"Non Cross Syn./Volume", 9,
    averageTotalResults[counterTwo+7]/totalNeuronVolume);
    setResult(thirdTitleArray[a]+"_"+"Total Syn./Length", 9,
    (averageTotalResults[counterTwo+6]+averageTotalResults[counterTwo+7])/totalNeuronLength);
    setResult(thirdTitleArray[a]+"_"+"Total Syn./Volume", 9,
    (averageTotalResults[counterTwo+6]+averageTotalResults[counterTwo+7])/totalNeuronVolume);
    counterTwo=counterTwo+8;
    updateResults();
    selectWindow("Results");
    saveAs("Results", argument+"Binned_"+thirdTitleArray[a]+".xls");
    selectWindow("Results");
    run("Close");
}
selectImage("Total_Results");
saveAs("TIFF", argument+"_Total.tif");
selectImage("Binned_Results");
saveAs("TIFF", argument+"_Binned.tif");
```
