# Supplementary material for: Computer Simulations Support a Morphological Contribution to BDNF Enhancement of Action Potential Generation
Source: Front Cell Neurosci. 2016 Sep 14;10:209. doi: 10.3389/fncel.2016.00209 (PMC5021759; doi:10.3389/fncel.2016.00209)
Supplement: DATA SHEET 2 — The NEURON code used to generate the computer simulations. [file Data_Sheet_2.PDF]

```
/*Modeling the effect of the geometrical distribution of excitatory and inhibitory synapses
derived from morphological
 *data on neuronal spiking.
 *
 *This is a method for analyzing how an observed distribution of VGlut1 and VGAT synaptic
 puncta influence spiking.
 * This takes VGlut1 and VGAT puncta information (distance from soma and intensity) for all
 puncta across a defined
 * region of the dendritic arbor and converts the information into relative frequency
 distributions for a given
 * sholl radius. Synapses are modeled according to the following rules.
 *  Double exponential Exp2Delays
 *          e = 0 for excitatory; -70 for inhibitory
 *          tau1= 0.2 for excitatory; 0.3 for inhibitory
 *          tau2= 4 for excitatory; 6 for inhibitory
 *          onset= stochastic within a defined integration window (typically 200 ms)
 *          conductance= pulled from a distribution of puncta intensities (median is 1 nS)
 *
 *
 * The synapses are then mapped onto simplified arbors using the following rules (Build
 Morphology and Specify Geometry hoc) :
 *
 * Dendrites= sholl crossings for a given sholl radius
 * Volume of Individual Dendrite Cylinder= total GFP volume for a given sholl radius/Dendrites
 * Diameter of Individual Dendrite Cylinder= Vol=(2pir^2)*2 and solve for r
 *
 * nsegs for each section= dlambda with 0.1
 *
 * The submodels:
 * Distance-X% of total synapses in each sholl radius are successively stimulated (to identify
 which regions of arbor contribute most)
 *
 * Balanced-X% of total synapses in each sholl radius are stimulated simultaneously  (to
 identify the neuronal response to balanced
 * stimulation across the whole neuron)
 *
 * Weighted-X% of total synapses on neuron are distributed according to their probabilistic
 distance from the soma (to identify the neuronal
 * response that most closely mimics the observed distributions. A neuron with a greater
 percentage of its synapses proximal will receive a
 * greater percentage of its stimulation in the proximal region
 *
 * Excitatory Model- There is no inhibition.
 * Inhibitory Model- Synapses have inhibitory characteristics depending on the EI ratio for a
 given sholl radius.
 *
 * For each model the output is spikes (axon cross 0 mV) and the max delta somatic membrane
 potential.
 *
 * The file "Robustness Shell.hoc" runs the program in the NEURON simulation environment.
 However, subfiles are needed for this program to run. The subfiles are listed sequentially in
 this document and they must be saved in the same folder as "Robustness_Shell.hoc".
 * The NEURON subfiles are: "Build Arbor Topologies Apical.hoc", "Specify Geometry Apical.hoc",
 "Weighted ExcitInhib Apical.hoc", "Run_Apical.hoc", "Run_ExDelay.hoc", "Run_InDelay.hoc",
 "Run_IntWindow.hoc", "Run_PassDend.hoc"
 * The text subfiles are "BDNF Morph MM.txt", "BDNF VGAT Ints.txt", "BDNF VGlut1 Ints.txt",
 "BDNF Weight Matrix.txt", "Vehicle Morph MM.txt", "Vehicle VGAT Ints.txt", "Vehicle VGlut1
 Ints.txt", "Veh Weighted Matrix.txt"
 */

/*
Robustness Shell.hoc

This file is set to run 20 times using 20 different random numbers for the stochastic
activation of different synapses
*/

for rando=1, rando=20{
    randSeed=rando
```

```
    xopen("Run_Apical.hoc")
    xopen("Run_ExDelay.hoc")
    xopen("Run_InDelay.hoc")
    xopen("Run_IntWindow.hoc")
    xopen("Run_PassDend.hoc")
}

/*
Run_Apical.hoc

This file modulates the size of the apical dendrite
*/


/**************************
*Global Setup         *
**************************/
getcwd()
chdir("/cygdrive/c/Users/Nick/Desktop/Neuron Model/11")
trials=1                     //1 trial equals 1 run through all cells specified
cellStart=1                  //This can be used to analyze a subset of cells
cellEnd=63                   //Example, to analyze cells 1-10: cellStart=1, cellEnd=10
cells =(cellEnd-cellStart)+1             //This generates the total number of cells that
will be analyzed                 //This is the length of time where synapses can be activated
objref randNum                   //This generates the random number for the model
randNum = new Random(randSeed)


naCond=0.03*1
kCond=0.0009*1
apicalVal=250*1
exDelay=4*1
inDelay=4*1
burstdur=100*1

strdef trailing, model
trailing = "_1_1_1_1_1"
model = "PassDend"




naCond=0.03*1
kCond=0.0009*1
apicalVal=250*0.25
exDelay=4*1
inDelay=4*1
burstdur=100*1


model= "Apical"
trailing = "_1_-25_1_1_1"

/**************************
*Vehicle Setup         *
**************************/
strdef weight, dist, rawdatafile, extension, weightexcit, filename
strdef vglut1intensities, vgatintensities, morphologydata
dist = "Veh Dist Matrix.txt"
weight = "Veh Weight Matrix.txt"
weightexcit = "Veh Weight Matrix Excit.txt"
vglut1intensities= "Vehicle VGlut1 Ints.txt"
vgatintensities= "Vehicle VGAT Ints.txt"
morphologydata="Vehicle Morph MM.txt"
rawdatafile = "_Vehicle"
extension = ".dat"

objref Raw_Data, Raw_Data_File                  //Create a destination file for raw data
generated during run
```

```
objref VGlut1_Int_Matrix, VGlut1File                //Create source data for VGlut1 intensities
VGlut1_Int_Matrix= new Matrix()
VGlut1File= new File()
VGlut1File.ropen(vglut1intensities)
VGlut1_Int_Matrix.scanf(VGlut1File, 100, 9)

objref  VGAT_Int_Matrix, VGATFile               //Create source data for VGAT intensities
VGAT_Int_Matrix= new Matrix()
VGATFile= new File()
VGATFile.ropen(vgatintensities)
VGAT_Int_Matrix.scanf(VGATFile, 100, 9)

objref morphMatrix, morphFile                   //Create source data for arbor morphology
morphMatrix = new Matrix()
morphFile = new File()
morphFile.ropen(morphologydata)
morphMatrix.scanf(morphFile,63,37)

/**************************
*Run through each model
*for each cell trials no.
*of times         *
***************************/

for tr=0, tr=trials-1 {
    cellCounter=0
    sprint(filename, "%d%s%s%s%s", randSeed, rawdatafile, model, trailing, extension)
    print filename
    Raw_Data = new Matrix(cells+2, 5)
    Raw_Data_File = new File(filename)
    Raw_Data_File.wopen(filename)
    for i=cellStart-1, i=cellEnd-1 {
        xopen("Build Arbor Topologies Apical.hoc")
        xopen("Specify Geometry Apical.hoc")
        finitialize(-70)
        xopen("Weighted ExcitInhib Apical.hoc")
        cellCounter+=1
    }
    Raw_Data.fprint(Raw_Data_File)
    Raw_Data_File.close()
}


/**************************
*BDNF Setup       *
***************************/
strdef weight, dist, rawdatafile, extension, weightexcit, filename
strdef vglut1intensities, vgatintensities, morphologydata   //String defs to allow the same
model to be
                                //run with different source data (ie Veh or BDNF)
dist = "BDNF Dist Matrix.txt"
weight = "BDNF Weight Matrix.txt"
weightexcit = "BDNF Weight Matrix Excit.txt"
vglut1intensities= "BDNF VGlut1 Ints.txt"           //The name of the matrix for the VGlut1
intensities
vgatintensities= "BDNF VGAT Ints.txt"               //The name of the matrix for the VGAT
intensities
morphologydata="BDNF Morph MM.txt"              //The name of the matrix for the morphology
parameters (ie, no. dendrites and diameters)
rawdatafile = "_BDNF"               //The name of the raw output file
extension = ".dat"

objref Raw_Data, Raw_Data_File                  //Create a destination file for raw data
generated during run

objref VGlut1_Int_Matrix, VGlut1File                //Create source data for VGlut1 intensities
VGlut1_Int_Matrix= new Matrix()
```

```
VGlut1File= new File()
VGlut1File.ropen(vglut1intensities)
VGlut1_Int_Matrix.scanf(VGlut1File, 100, 9)

objref  VGAT_Int_Matrix, VGATFile               //Create source data for VGAT intensities
VGAT_Int_Matrix= new Matrix()
VGATFile= new File()
VGATFile.ropen(vgatintensities)
VGAT_Int_Matrix.scanf(VGATFile, 100, 9)

objref morphMatrix, morphFile                   //Create source data for arbor morphology
morphMatrix = new Matrix()
morphFile = new File()
morphFile.ropen(morphologydata)
morphMatrix.scanf(morphFile,63,37)

/***************************
*Run through each model
*for each cell trials no.
*of times          *
***************************/
for tr=0, tr=trials-1 {
    cellCounter=0
    sprint(filename, "%d%s%s%s%s", randSeed, rawdatafile, model, trailing, extension)
    print filename
    Raw_Data = new Matrix(cells+2, 5)
    Raw_Data_File = new File(filename)
    Raw_Data_File.wopen(filename)
    for i=cellStart-1, i=cellEnd-1 {
        xopen("Build Arbor Topologies Apical.hoc")
        xopen("Specify Geometry Apical.hoc")
        finitialize(-70)
        xopen("Weighted ExcitInhib Apical.hoc")
        cellCounter+=1
    }
    Raw_Data.fprint(Raw_Data_File)
    Raw_Data_File.close()
}

naCond=0.03*1
kCond=0.0009*1
apicalVal=250*0.5
exDelay=4*1
inDelay=4*1
burstdur=100*1


model= "Apical"
trailing = "_1_-5_1_1_1"

/***************************
*Vehicle Setup          *
***************************/
strdef weight, dist, rawdatafile, extension, weightexcit, filename
strdef vglut1intensities, vgatintensities, morphologydata
dist = "Veh Dist Matrix.txt"
weight = "Veh Weight Matrix.txt"
weightexcit = "Veh Weight Matrix Excit.txt"
vglut1intensities= "Vehicle VGlut1 Ints.txt"
vgatintensities= "Vehicle VGAT Ints.txt"
morphologydata="Vehicle Morph MM.txt"
rawdatafile = "_Vehicle"
extension = ".dat"

objref Raw_Data, Raw_Data_File                  //Create a destination file for raw data
generated during run


objref VGlut1_Int_Matrix, VGlut1File                //Create source data for VGlut1 intensities
```

```
VGlut1_Int_Matrix= new Matrix()
VGlut1File= new File()
VGlut1File.ropen(vglut1intensities)
VGlut1_Int_Matrix.scanf(VGlut1File, 100, 9)

objref  VGAT_Int_Matrix, VGATFile               //Create source data for VGAT intensities
VGAT_Int_Matrix= new Matrix()
VGATFile= new File()
VGATFile.ropen(vgatintensities)
VGAT_Int_Matrix.scanf(VGATFile, 100, 9)

objref morphMatrix, morphFile                   //Create source data for arbor morphology
morphMatrix = new Matrix()
morphFile = new File()
morphFile.ropen(morphologydata)
morphMatrix.scanf(morphFile,63,37)

/**************************
*Run through each model
*for each cell trials no.
*of times          *
***************************/

for tr=0, tr=trials-1 {
    cellCounter=0
    sprint(filename, "%d%s%s%s%s", randSeed, rawdatafile, model, trailing, extension)
    print filename
    Raw_Data = new Matrix(cells+2, 5)
    Raw_Data_File = new File(filename)
    Raw_Data_File.wopen(filename)
    for i=cellStart-1, i=cellEnd-1 {
        xopen("Build Arbor Topologies Apical.hoc")
        xopen("Specify Geometry Apical.hoc")
        finitialize(-70)
        xopen("Weighted ExcitInhib Apical.hoc")
        cellCounter+=1
    }
    Raw_Data.fprint(Raw_Data_File)
    Raw_Data_File.close()
}


/**************************
*BDNF Setup        *
***************************/
strdef weight, dist, rawdatafile, extension, weightexcit, filename
strdef vglut1intensities, vgatintensities, morphologydata   //String defs to allow the same
model to be
                                //run with different source data (ie Veh or BDNF)
dist = "BDNF Dist Matrix.txt"
weight = "BDNF Weight Matrix.txt"
weightexcit = "BDNF Weight Matrix Excit.txt"
vglut1intensities= "BDNF VGlut1 Ints.txt"           //The name of the matrix for the VGlut1
intensities
vgatintensities= "BDNF VGAT Ints.txt"               //The name of the matrix for the VGAT
intensities
morphologydata="BDNF Morph MM.txt"              //The name of the matrix for the morphology
parameters (ie, no. dendrites and diameters)
rawdatafile = "_BDNF"               //The name of the raw output file
extension = ".dat"

objref Raw_Data, Raw_Data_File                  //Create a destination file for raw data
generated during run

objref VGlut1_Int_Matrix, VGlut1File                //Create source data for VGlut1 intensities
VGlut1_Int_Matrix= new Matrix()
VGlut1File= new File()
VGlut1File.ropen(vglut1intensities)
VGlut1_Int_Matrix.scanf(VGlut1File, 100, 9)
```

```
objref  VGAT_Int_Matrix, VGATFile                //Create source data for VGAT intensities
VGAT_Int_Matrix= new Matrix()
VGATFile= new File()
VGATFile.ropen(vgatintensities)
VGAT_Int_Matrix.scanf(VGATFile, 100, 9)

objref morphMatrix, morphFile                    //Create source data for arbor morphology
morphMatrix = new Matrix()
morphFile = new File()
morphFile.ropen(morphologydata)
morphMatrix.scanf(morphFile,63,37)

/**************************
*Run through each model
*for each cell trials no.
*of times         *
***************************/
for tr=0, tr=trials-1 {
    cellCounter=0
    sprint(filename, "%d%s%s%s%s", randSeed, rawdatafile, model, trailing, extension)
    print filename
    Raw_Data = new Matrix(cells+2, 5)
    Raw_Data_File = new File(filename)
    Raw_Data_File.wopen(filename)
    for i=cellStart-1, i=cellEnd-1 {
        xopen("Build Arbor Topologies Apical.hoc")
        xopen("Specify Geometry Apical.hoc")
        finitialize(-70)
        xopen("Weighted ExcitInhib Apical.hoc")
        cellCounter+=1
    }
    Raw_Data.fprint(Raw_Data_File)
    Raw_Data_File.close()
}

naCond=0.03*1
kCond=0.0009*1
apicalVal=250*2
exDelay=4*1
inDelay=4*1
burstdur=100*1


model= "Apical"
trailing = "_1_2_1_1_1"

/**************************
*Vehicle Setup        *
***************************/
strdef weight, dist, rawdatafile, extension, weightexcit, filename
strdef vglut1intensities, vgatintensities, morphologydata
dist = "Veh Dist Matrix.txt"
weight = "Veh Weight Matrix.txt"
weightexcit = "Veh Weight Matrix Excit.txt"
vglut1intensities= "Vehicle VGlut1 Ints.txt"
vgatintensities= "Vehicle VGAT Ints.txt"
morphologydata="Vehicle Morph MM.txt"
rawdatafile = "_Vehicle"
extension = ".dat"

objref Raw_Data, Raw_Data_File                   //Create a destination file for raw data
generated during run


objref VGlut1_Int_Matrix, VGlut1File                //Create source data for VGlut1 intensities
VGlut1_Int_Matrix= new Matrix()
VGlut1File= new File()
VGlut1File.ropen(vglut1intensities)
```

```
VGlut1_Int_Matrix.scanf(VGlut1File, 100, 9)

objref  VGAT_Int_Matrix, VGATFile               //Create source data for VGAT intensities
VGAT_Int_Matrix= new Matrix()
VGATFile= new File()
VGATFile.ropen(vgatintensities)
VGAT_Int_Matrix.scanf(VGATFile, 100, 9)

objref morphMatrix, morphFile                   //Create source data for arbor morphology
morphMatrix = new Matrix()
morphFile = new File()
morphFile.ropen(morphologydata)
morphMatrix.scanf(morphFile,63,37)

/***************************
*Run through each model
*for each cell trials no.
*of times          *
***************************/

for tr=0, tr=trials-1 {
    cellCounter=0
    sprint(filename, "%d%s%s%s%s", randSeed, rawdatafile, model, trailing, extension)
    print filename
    Raw_Data = new Matrix(cells+2, 5)
    Raw_Data_File = new File(filename)
    Raw_Data_File.wopen(filename)
    for i=cellStart-1, i=cellEnd-1 {
        xopen("Build Arbor Topologies Apical.hoc")
        xopen("Specify Geometry Apical.hoc")
        finitialize(-70)
        xopen("Weighted ExcitInhib Apical.hoc")
        cellCounter+=1
    }
    Raw_Data.fprint(Raw_Data_File)
    Raw_Data_File.close()
}


/***************************
*BDNF Setup        *
***************************/
strdef weight, dist, rawdatafile, extension, weightexcit, filename
strdef vglut1intensities, vgatintensities, morphologydata   //String defs to allow the same
model to be
                                //run with different source data (ie Veh or BDNF)
dist = "BDNF Dist Matrix.txt"
weight = "BDNF Weight Matrix.txt"
weightexcit = "BDNF Weight Matrix Excit.txt"
vglut1intensities= "BDNF VGlut1 Ints.txt"           //The name of the matrix for the VGlut1
intensities
vgatintensities= "BDNF VGAT Ints.txt"               //The name of the matrix for the VGAT
intensities
morphologydata="BDNF Morph MM.txt"              //The name of the matrix for the morphology
parameters (ie, no. dendrites and diameters)
rawdatafile = "_BDNF"               //The name of the raw output file
extension = ".dat"

objref Raw_Data, Raw_Data_File                  //Create a destination file for raw data
generated during run

objref VGlut1_Int_Matrix, VGlut1File                //Create source data for VGlut1 intensities
VGlut1_Int_Matrix= new Matrix()
VGlut1File= new File()
VGlut1File.ropen(vglut1intensities)
VGlut1_Int_Matrix.scanf(VGlut1File, 100, 9)

objref  VGAT_Int_Matrix, VGATFile               //Create source data for VGAT intensities
VGAT_Int_Matrix= new Matrix()
```

```
VGATFile= new File()
VGATFile.ropen(vgatintensities)
VGAT_Int_Matrix.scanf(VGATFile, 100, 9)

objref morphMatrix, morphFile                   //Create source data for arbor morphology
morphMatrix = new Matrix()
morphFile = new File()
morphFile.ropen(morphologydata)
morphMatrix.scanf(morphFile,63,37)

/**************************
*Run through each model
*for each cell trials no.
*of times         *
***************************/
for tr=0, tr=trials-1 {
    cellCounter=0
    sprint(filename, "%d%s%s%s%s", randSeed, rawdatafile, model, trailing, extension)
    print filename
    Raw_Data = new Matrix(cells+2, 5)
    Raw_Data_File = new File(filename)
    Raw_Data_File.wopen(filename)
    for i=cellStart-1, i=cellEnd-1 {
        xopen("Build Arbor Topologies Apical.hoc")
        xopen("Specify Geometry Apical.hoc")
        finitialize(-70)
        xopen("Weighted ExcitInhib Apical.hoc")
        cellCounter+=1
    }
    Raw_Data.fprint(Raw_Data_File)
    Raw_Data_File.close()
}

naCond=0.03*1
kCond=0.0009*1
apicalVal=250*4
exDelay=4*1
inDelay=4*1
burstdur=100*1

model= "Apical"
trailing = "_1_4_1_1_1"

/**************************
*Vehicle Setup        *
***************************/
strdef weight, dist, rawdatafile, extension, weightexcit, filename
strdef vglut1intensities, vgatintensities, morphologydata
dist = "Veh Dist Matrix.txt"
weight = "Veh Weight Matrix.txt"
weightexcit = "Veh Weight Matrix Excit.txt"
vglut1intensities= "Vehicle VGlut1 Ints.txt"
vgatintensities= "Vehicle VGAT Ints.txt"
morphologydata="Vehicle Morph MM.txt"
rawdatafile = "_Vehicle"
extension = ".dat"

objref Raw_Data, Raw_Data_File                  //Create a destination file for raw data
generated during run


objref VGlut1_Int_Matrix, VGlut1File                //Create source data for VGlut1 intensities
VGlut1_Int_Matrix= new Matrix()
VGlut1File= new File()
VGlut1File.ropen(vglut1intensities)
VGlut1_Int_Matrix.scanf(VGlut1File, 100, 9)

objref  VGAT_Int_Matrix, VGATFile               //Create source data for VGAT intensities
VGAT_Int_Matrix= new Matrix()
```

```
VGATFile= new File()
VGATFile.ropen(vgatintensities)
VGAT_Int_Matrix.scanf(VGATFile, 100, 9)

objref morphMatrix, morphFile                    //Create source data for arbor morphology
morphMatrix = new Matrix()
morphFile = new File()
morphFile.ropen(morphologydata)
morphMatrix.scanf(morphFile,63,37)

/**************************
*Run through each model
*for each cell trials no.
*of times         *
***************************/

for tr=0, tr=trials-1 {
    cellCounter=0
    sprint(filename, "%d%s%s%s%s", randSeed, rawdatafile, model, trailing, extension)
    print filename
    Raw_Data = new Matrix(cells+2, 5)
    Raw_Data_File = new File(filename)
    Raw_Data_File.wopen(filename)
    for i=cellStart-1, i=cellEnd-1 {
        xopen("Build Arbor Topologies Apical.hoc")
        xopen("Specify Geometry Apical.hoc")
        finitialize(-70)
        xopen("Weighted ExcitInhib Apical.hoc")
        cellCounter+=1
    }
    Raw_Data.fprint(Raw_Data_File)
    Raw_Data_File.close()
}


/**************************
*BDNF Setup       *
***************************/
strdef weight, dist, rawdatafile, extension, weightexcit, filename
strdef vglut1intensities, vgatintensities, morphologydata   //String defs to allow the same
model to be
                                //run with different source data (ie Veh or BDNF)
dist = "BDNF Dist Matrix.txt"
weight = "BDNF Weight Matrix.txt"
weightexcit = "BDNF Weight Matrix Excit.txt"
vglut1intensities= "BDNF VGlut1 Ints.txt"           //The name of the matrix for the VGlut1
intensities
vgatintensities= "BDNF VGAT Ints.txt"               //The name of the matrix for the VGAT
intensities
morphologydata="BDNF Morph MM.txt"              //The name of the matrix for the morphology
parameters (ie, no. dendrites and diameters)
rawdatafile = "_BDNF"                //The name of the raw output file
extension = ".dat"

objref Raw_Data, Raw_Data_File                   //Create a destination file for raw data
generated during run

objref VGlut1_Int_Matrix, VGlut1File                //Create source data for VGlut1 intensities
VGlut1_Int_Matrix= new Matrix()
VGlut1File= new File()
VGlut1File.ropen(vglut1intensities)
VGlut1_Int_Matrix.scanf(VGlut1File, 100, 9)

objref  VGAT_Int_Matrix, VGATFile                //Create source data for VGAT intensities
VGAT_Int_Matrix= new Matrix()
VGATFile= new File()
VGATFile.ropen(vgatintensities)
VGAT_Int_Matrix.scanf(VGATFile, 100, 9)
```

```
objref morphMatrix, morphFile                    //Create source data for arbor morphology
morphMatrix = new Matrix()
morphFile = new File()
morphFile.ropen(morphologydata)
morphMatrix.scanf(morphFile,63,37)

/**************************
*Run through each model
*for each cell trials no.
*of times         *
***************************/
for tr=0, tr=trials-1 {
    cellCounter=0
    sprint(filename, "%d%s%s%s%s", randSeed, rawdatafile, model, trailing, extension)
    print filename
    Raw_Data = new Matrix(cells+2, 5)
    Raw_Data_File = new File(filename)
    Raw_Data_File.wopen(filename)
    for i=cellStart-1, i=cellEnd-1 {
        xopen("Build Arbor Topologies Apical.hoc")
        xopen("Specify Geometry Apical.hoc")
        finitialize(-70)
        xopen("Weighted ExcitInhib Apical.hoc")
        cellCounter+=1
    }
    Raw_Data.fprint(Raw_Data_File)
    Raw_Data_File.close()
}


/*
Run_ExDelay.hoc

This file modulates the decay time of the excitatory synapses
*/
/**************************
*Global Setup          *
***************************/
getcwd()
chdir("/cygdrive/c/Users/Nick/Desktop/Neuron Model/11")
trials=1                          //1 trial equals 1 run through all cells specified
cellStart=1                       //This can be used to analyze a subset of cells
cellEnd=63                        //Example, to analyze cells 1-10: cellStart=1, cellEnd=10
cells =(cellEnd-cellStart)+1                  //This generates the total number of cells that
will be analyzed                      //This is the length of time where synapses can be activated
objref randNum                        //This generates the random number for the model
randNum = new Random(randSeed)


naCond=0.03*1
kCond=0.0009*1
apicalVal=250*1
exDelay=4*1
inDelay=4*1
burstdur=100*1

strdef trailing, model
trailing = "_1_1_1_1_1"
model = "PassDend"




naCond=0.03*1
kCond=0.0009*1
apicalVal=250*1
exDelay=4*0.25
inDelay=4*1
burstdur=100*1
```

```
model= "ExDelay"
trailing = "_1_1_-25_1_1"

/**************************
*Vehicle Setup        *
***************************/
strdef weight, dist, rawdatafile, extension, weightexcit, filename
strdef vglut1intensities, vgatintensities, morphologydata
dist = "Veh Dist Matrix.txt"
weight = "Veh Weight Matrix.txt"
weightexcit = "Veh Weight Matrix Excit.txt"
vglut1intensities= "Vehicle VGlut1 Ints.txt"
vgatintensities= "Vehicle VGAT Ints.txt"
morphologydata="Vehicle Morph MM.txt"
rawdatafile = "_Vehicle"
extension = ".dat"

objref Raw_Data, Raw_Data_File                    //Create a destination file for raw data
generated during run


objref VGlut1_Int_Matrix, VGlut1File                //Create source data for VGlut1 intensities
VGlut1_Int_Matrix= new Matrix()
VGlut1File= new File()
VGlut1File.ropen(vglut1intensities)
VGlut1_Int_Matrix.scanf(VGlut1File, 100, 9)

objref  VGAT_Int_Matrix, VGATFile                 //Create source data for VGAT intensities
VGAT_Int_Matrix= new Matrix()
VGATFile= new File()
VGATFile.ropen(vgatintensities)
VGAT_Int_Matrix.scanf(VGATFile, 100, 9)

objref morphMatrix, morphFile                     //Create source data for arbor morphology
morphMatrix = new Matrix()
morphFile = new File()
morphFile.ropen(morphologydata)
morphMatrix.scanf(morphFile,63,37)

/**************************
*Run through each model
*for each cell trials no.
*of times         *
***************************/

for tr=0, tr=trials-1 {
    cellCounter=0
    sprint(filename, "%d%s%s%s%s", randSeed, rawdatafile, model, trailing, extension)
    print filename
    Raw_Data = new Matrix(cells+2, 5)
    Raw_Data_File = new File(filename)
    Raw_Data_File.wopen(filename)
    for i=cellStart-1, i=cellEnd-1 {
        xopen("Build Arbor Topologies Apical.hoc")
        xopen("Specify Geometry Apical.hoc")
        finitialize(-70)
        xopen("Weighted ExcitInhib Apical.hoc")
        cellCounter+=1
    }
    Raw_Data.fprint(Raw_Data_File)
    Raw_Data_File.close()
}


/**************************
*BDNF Setup       *
***************************/
```

```
strdef weight, dist, rawdatafile, extension, weightexcit, filename
strdef vglut1intensities, vgatintensities, morphologydata   //String defs to allow the same
model to be
                                //run with different source data (ie Veh or BDNF)
dist = "BDNF Dist Matrix.txt"
weight = "BDNF Weight Matrix.txt"
weightexcit = "BDNF Weight Matrix Excit.txt"
vglut1intensities= "BDNF VGlut1 Ints.txt"           //The name of the matrix for the VGlut1
intensities
vgatintensities= "BDNF VGAT Ints.txt"               //The name of the matrix for the VGAT
intensities
morphologydata="BDNF Morph MM.txt"              //The name of the matrix for the morphology
parameters (ie, no. dendrites and diameters)
rawdatafile = "_BDNF"               //The name of the raw output file
extension = ".dat"

objref Raw_Data, Raw_Data_File                  //Create a destination file for raw data
generated during run

objref VGlut1_Int_Matrix, VGlut1File                //Create source data for VGlut1 intensities
VGlut1_Int_Matrix= new Matrix()
VGlut1File= new File()
VGlut1File.ropen(vglut1intensities)
VGlut1_Int_Matrix.scanf(VGlut1File, 100, 9)

objref  VGAT_Int_Matrix, VGATFile               //Create source data for VGAT intensities
VGAT_Int_Matrix= new Matrix()
VGATFile= new File()
VGATFile.ropen(vgatintensities)
VGAT_Int_Matrix.scanf(VGATFile, 100, 9)

objref morphMatrix, morphFile                   //Create source data for arbor morphology
morphMatrix = new Matrix()
morphFile = new File()
morphFile.ropen(morphologydata)
morphMatrix.scanf(morphFile,63,37)

/***************************
*Run through each model
*for each cell trials no.
*of times          *
***************************/
for tr=0, tr=trials-1 {
    cellCounter=0
    sprint(filename, "%d%s%s%s%s", randSeed, rawdatafile, model, trailing, extension)
    print filename
    Raw_Data = new Matrix(cells+2, 5)
    Raw_Data_File = new File(filename)
    Raw_Data_File.wopen(filename)
    for i=cellStart-1, i=cellEnd-1 {
        xopen("Build Arbor Topologies Apical.hoc")
        xopen("Specify Geometry Apical.hoc")
        finitialize(-70)
        xopen("Weighted ExcitInhib Apical.hoc")
        cellCounter+=1
    }
    Raw_Data.fprint(Raw_Data_File)
    Raw_Data_File.close()
}

naCond=0.03*1
kCond=0.0009*1
apicalVal=250*1
exDelay=4*0.5
inDelay=4*1
burstdur=100*1


model= "ExDelay"
```

```
trailing = "_1_1_-5_1_1"

/*************************
*Vehicle Setup         *
**************************/
strdef weight, dist, rawdatafile, extension, weightexcit, filename
strdef vglut1intensities, vgatintensities, morphologydata
dist = "Veh Dist Matrix.txt"
weight = "Veh Weight Matrix.txt"
weightexcit = "Veh Weight Matrix Excit.txt"
vglut1intensities= "Vehicle VGlut1 Ints.txt"
vgatintensities= "Vehicle VGAT Ints.txt"
morphologydata="Vehicle Morph MM.txt"
rawdatafile = "_Vehicle"
extension = ".dat"

objref Raw_Data, Raw_Data_File                  //Create a destination file for raw data
generated during run


objref VGlut1_Int_Matrix, VGlut1File                //Create source data for VGlut1 intensities
VGlut1_Int_Matrix= new Matrix()
VGlut1File= new File()
VGlut1File.ropen(vglut1intensities)
VGlut1_Int_Matrix.scanf(VGlut1File, 100, 9)

objref  VGAT_Int_Matrix, VGATFile               //Create source data for VGAT intensities
VGAT_Int_Matrix= new Matrix()
VGATFile= new File()
VGATFile.ropen(vgatintensities)
VGAT_Int_Matrix.scanf(VGATFile, 100, 9)

objref morphMatrix, morphFile                   //Create source data for arbor morphology
morphMatrix = new Matrix()
morphFile = new File()
morphFile.ropen(morphologydata)
morphMatrix.scanf(morphFile,63,37)

/*************************
*Run through each model
*for each cell trials no.
*of times          *
**************************/

for tr=0, tr=trials-1 {
    cellCounter=0
    sprint(filename, "%d%s%s%s%s", randSeed, rawdatafile, model, trailing, extension)
    print filename
    Raw_Data = new Matrix(cells+2, 5)
    Raw_Data_File = new File(filename)
    Raw_Data_File.wopen(filename)
    for i=cellStart-1, i=cellEnd-1 {
        xopen("Build Arbor Topologies Apical.hoc")
        xopen("Specify Geometry Apical.hoc")
        finitialize(-70)
        xopen("Weighted ExcitInhib Apical.hoc")
        cellCounter+=1
    }
    Raw_Data.fprint(Raw_Data_File)
    Raw_Data_File.close()
}


/*************************
*BDNF Setup         *
**************************/
strdef weight, dist, rawdatafile, extension, weightexcit, filename
strdef vglut1intensities, vgatintensities, morphologydata   //String defs to allow the same
model to be
```

```
                                //run with different source data (ie Veh or BDNF)
dist = "BDNF Dist Matrix.txt"
weight = "BDNF Weight Matrix.txt"
weightexcit = "BDNF Weight Matrix Excit.txt"
vglut1intensities= "BDNF VGlut1 Ints.txt"           //The name of the matrix for the VGlut1
intensities
vgatintensities= "BDNF VGAT Ints.txt"               //The name of the matrix for the VGAT
intensities
morphologydata="BDNF Morph MM.txt"              //The name of the matrix for the morphology
parameters (ie, no. dendrites and diameters)
rawdatafile = "_BDNF"               //The name of the raw output file
extension = ".dat"

objref Raw_Data, Raw_Data_File                  //Create a destination file for raw data
generated during run

objref VGlut1_Int_Matrix, VGlut1File                //Create source data for VGlut1 intensities
VGlut1_Int_Matrix= new Matrix()
VGlut1File= new File()
VGlut1File.ropen(vglut1intensities)
VGlut1_Int_Matrix.scanf(VGlut1File, 100, 9)

objref  VGAT_Int_Matrix, VGATFile               //Create source data for VGAT intensities
VGAT_Int_Matrix= new Matrix()
VGATFile= new File()
VGATFile.ropen(vgatintensities)
VGAT_Int_Matrix.scanf(VGATFile, 100, 9)

objref morphMatrix, morphFile                   //Create source data for arbor morphology
morphMatrix = new Matrix()
morphFile = new File()
morphFile.ropen(morphologydata)
morphMatrix.scanf(morphFile,63,37)

/***************************
*Run through each model
*for each cell trials no.
*of times         *
***************************/
for tr=0, tr=trials-1 {
    cellCounter=0
    sprint(filename, "%d%s%s%s%s", randSeed, rawdatafile, model, trailing, extension)
    print filename
    Raw_Data = new Matrix(cells+2, 5)
    Raw_Data_File = new File(filename)
    Raw_Data_File.wopen(filename)
    for i=cellStart-1, i=cellEnd-1 {
        xopen("Build Arbor Topologies Apical.hoc")
        xopen("Specify Geometry Apical.hoc")
        finitialize(-70)
        xopen("Weighted ExcitInhib Apical.hoc")
        cellCounter+=1
    }
    Raw_Data.fprint(Raw_Data_File)
    Raw_Data_File.close()
}

naCond=0.03*1
kCond=0.0009*1
apicalVal=250*1
exDelay=4*2
inDelay=4*1
burstdur=100*1


model= "ExDelay"
trailing = "_1_1_2_1_1"

/***************************
```

```
*Vehicle Setup        *
**************************/
strdef weight, dist, rawdatafile, extension, weightexcit, filename
strdef vglut1intensities, vgatintensities, morphologydata
dist = "Veh Dist Matrix.txt"
weight = "Veh Weight Matrix.txt"
weightexcit = "Veh Weight Matrix Excit.txt"
vglut1intensities= "Vehicle VGlut1 Ints.txt"
vgatintensities= "Vehicle VGAT Ints.txt"
morphologydata="Vehicle Morph MM.txt"
rawdatafile = "_Vehicle"
extension = ".dat"

objref Raw_Data, Raw_Data_File                  //Create a destination file for raw data
generated during run


objref VGlut1_Int_Matrix, VGlut1File                //Create source data for VGlut1 intensities
VGlut1_Int_Matrix= new Matrix()
VGlut1File= new File()
VGlut1File.ropen(vglut1intensities)
VGlut1_Int_Matrix.scanf(VGlut1File, 100, 9)

objref  VGAT_Int_Matrix, VGATFile               //Create source data for VGAT intensities
VGAT_Int_Matrix= new Matrix()
VGATFile= new File()
VGATFile.ropen(vgatintensities)
VGAT_Int_Matrix.scanf(VGATFile, 100, 9)

objref morphMatrix, morphFile                   //Create source data for arbor morphology
morphMatrix = new Matrix()
morphFile = new File()
morphFile.ropen(morphologydata)
morphMatrix.scanf(morphFile,63,37)

/*************************
*Run through each model
*for each cell trials no.
*of times         *
**************************/

for tr=0, tr=trials-1 {
    cellCounter=0
    sprint(filename, "%d%s%s%s%s", randSeed, rawdatafile, model, trailing, extension)
    print filename
    Raw_Data = new Matrix(cells+2, 5)
    Raw_Data_File = new File(filename)
    Raw_Data_File.wopen(filename)
    for i=cellStart-1, i=cellEnd-1 {
        xopen("Build Arbor Topologies Apical.hoc")
        xopen("Specify Geometry Apical.hoc")
        finitialize(-70)
        xopen("Weighted ExcitInhib Apical.hoc")
        cellCounter+=1
    }
    Raw_Data.fprint(Raw_Data_File)
    Raw_Data_File.close()
}


/*************************
*BDNF Setup       *
**************************/
strdef weight, dist, rawdatafile, extension, weightexcit, filename
strdef vglut1intensities, vgatintensities, morphologydata   //String defs to allow the same
model to be
                                //run with different source data (ie Veh or BDNF)
dist = "BDNF Dist Matrix.txt"
weight = "BDNF Weight Matrix.txt"
```

```
weightexcit = "BDNF Weight Matrix Excit.txt"
vglut1intensities= "BDNF VGlut1 Ints.txt"           //The name of the matrix for the VGlut1
intensities
vgatintensities= "BDNF VGAT Ints.txt"               //The name of the matrix for the VGAT
intensities
morphologydata="BDNF Morph MM.txt"              //The name of the matrix for the morphology
parameters (ie, no. dendrites and diameters)
rawdatafile = "_BDNF"               //The name of the raw output file
extension = ".dat"

objref Raw_Data, Raw_Data_File                  //Create a destination file for raw data
generated during run

objref VGlut1_Int_Matrix, VGlut1File                //Create source data for VGlut1 intensities
VGlut1_Int_Matrix= new Matrix()
VGlut1File= new File()
VGlut1File.ropen(vglut1intensities)
VGlut1_Int_Matrix.scanf(VGlut1File, 100, 9)

objref  VGAT_Int_Matrix, VGATFile               //Create source data for VGAT intensities
VGAT_Int_Matrix= new Matrix()
VGATFile= new File()
VGATFile.ropen(vgatintensities)
VGAT_Int_Matrix.scanf(VGATFile, 100, 9)

objref morphMatrix, morphFile                   //Create source data for arbor morphology
morphMatrix = new Matrix()
morphFile = new File()
morphFile.ropen(morphologydata)
morphMatrix.scanf(morphFile,63,37)

/**************************
*Run through each model
*for each cell trials no.
*of times         *
**************************/
for tr=0, tr=trials-1 {
    cellCounter=0
    sprint(filename, "%d%s%s%s%s", randSeed, rawdatafile, model, trailing, extension)
    print filename
    Raw_Data = new Matrix(cells+2, 5)
    Raw_Data_File = new File(filename)
    Raw_Data_File.wopen(filename)
    for i=cellStart-1, i=cellEnd-1 {
        xopen("Build Arbor Topologies Apical.hoc")
        xopen("Specify Geometry Apical.hoc")
        finitialize(-70)
        xopen("Weighted ExcitInhib Apical.hoc")
        cellCounter+=1
    }
    Raw_Data.fprint(Raw_Data_File)
    Raw_Data_File.close()
}

naCond=0.03*1
kCond=0.0009*1
apicalVal=250*1
exDelay=4*4
inDelay=4*1
burstdur=100*1

model= "ExDelay"
trailing = "_1_1_4_1_1"

/**************************
*Vehicle Setup        *
**************************/
strdef weight, dist, rawdatafile, extension, weightexcit, filename
strdef vglut1intensities, vgatintensities, morphologydata
```

```
dist = "Veh Dist Matrix.txt"
weight = "Veh Weight Matrix.txt"
weightexcit = "Veh Weight Matrix Excit.txt"
vglut1intensities= "Vehicle VGlut1 Ints.txt"
vgatintensities= "Vehicle VGAT Ints.txt"
morphologydata="Vehicle Morph MM.txt"
rawdatafile = "_Vehicle"
extension = ".dat"

objref Raw_Data, Raw_Data_File                    //Create a destination file for raw data
generated during run


objref VGlut1_Int_Matrix, VGlut1File                //Create source data for VGlut1 intensities
VGlut1_Int_Matrix= new Matrix()
VGlut1File= new File()
VGlut1File.ropen(vglut1intensities)
VGlut1_Int_Matrix.scanf(VGlut1File, 100, 9)

objref  VGAT_Int_Matrix, VGATFile                 //Create source data for VGAT intensities
VGAT_Int_Matrix= new Matrix()
VGATFile= new File()
VGATFile.ropen(vgatintensities)
VGAT_Int_Matrix.scanf(VGATFile, 100, 9)

objref morphMatrix, morphFile                     //Create source data for arbor morphology
morphMatrix = new Matrix()
morphFile = new File()
morphFile.ropen(morphologydata)
morphMatrix.scanf(morphFile,63,37)

/**************************
*Run through each model
*for each cell trials no.
*of times          *
***************************/

for tr=0, tr=trials-1 {
    cellCounter=0
    sprint(filename, "%d%s%s%s%s", randSeed, rawdatafile, model, trailing, extension)
    print filename
    Raw_Data = new Matrix(cells+2, 5)
    Raw_Data_File = new File(filename)
    Raw_Data_File.wopen(filename)
    for i=cellStart-1, i=cellEnd-1 {
        xopen("Build Arbor Topologies Apical.hoc")
        xopen("Specify Geometry Apical.hoc")
        finitialize(-70)
        xopen("Weighted ExcitInhib Apical.hoc")
        cellCounter+=1
    }
    Raw_Data.fprint(Raw_Data_File)
    Raw_Data_File.close()
}


/**************************
*BDNF Setup        *
***************************/
strdef weight, dist, rawdatafile, extension, weightexcit, filename
strdef vglut1intensities, vgatintensities, morphologydata   //String defs to allow the same
model to be
                                //run with different source data (ie Veh or BDNF)
dist = "BDNF Dist Matrix.txt"
weight = "BDNF Weight Matrix.txt"
weightexcit = "BDNF Weight Matrix Excit.txt"
vglut1intensities= "BDNF VGlut1 Ints.txt"           //The name of the matrix for the VGlut1
intensities
vgatintensities= "BDNF VGAT Ints.txt"               //The name of the matrix for the VGAT
```

```
intensities
morphologydata="BDNF Morph MM.txt"              //The name of the matrix for the morphology
parameters (ie, no. dendrites and diameters)
rawdatafile = "_BDNF"                //The name of the raw output file
extension = ".dat"

objref Raw_Data, Raw_Data_File                  //Create a destination file for raw data
generated during run

objref VGlut1_Int_Matrix, VGlut1File                //Create source data for VGlut1 intensities
VGlut1_Int_Matrix= new Matrix()
VGlut1File= new File()
VGlut1File.ropen(vglut1intensities)
VGlut1_Int_Matrix.scanf(VGlut1File, 100, 9)

objref  VGAT_Int_Matrix, VGATFile               //Create source data for VGAT intensities
VGAT_Int_Matrix= new Matrix()
VGATFile= new File()
VGATFile.ropen(vgatintensities)
VGAT_Int_Matrix.scanf(VGATFile, 100, 9)

objref morphMatrix, morphFile                   //Create source data for arbor morphology
morphMatrix = new Matrix()
morphFile = new File()
morphFile.ropen(morphologydata)
morphMatrix.scanf(morphFile,63,37)

/**************************
*Run through each model
*for each cell trials no.
*of times         *
***************************/
for tr=0, tr=trials-1 {
    cellCounter=0
    sprint(filename, "%d%s%s%s%s", randSeed, rawdatafile, model, trailing, extension)
    print filename
    Raw_Data = new Matrix(cells+2, 5)
    Raw_Data_File = new File(filename)
    Raw_Data_File.wopen(filename)
    for i=cellStart-1, i=cellEnd-1 {
        xopen("Build Arbor Topologies Apical.hoc")
        xopen("Specify Geometry Apical.hoc")
        finitialize(-70)
        xopen("Weighted ExcitInhib Apical.hoc")
        cellCounter+=1
    }
    Raw_Data.fprint(Raw_Data_File)
    Raw_Data_File.close()
}


/*
Run_InDelay.hoc

This file modulates the decay time of the inhibitory synapses
*/

/**************************
*Global Setup         *
***************************/
getcwd()
chdir("/cygdrive/c/Users/Nick/Desktop/Neuron Model/11")
trials=1                        //1 trial equals 1 run through all cells specified
cellStart=1                     //This can be used to analyze a subset of cells
cellEnd=63                      //Example, to analyze cells 1-10: cellStart=1, cellEnd=10
cells =(cellEnd-cellStart)+1                //This generates the total number of cells that
will be analyzed                    //This is the length of time where synapses can be activated
objref randNum                      //This generates the random number for the model
randNum = new Random(randSeed)
```

```
naCond=0.03*1
kCond=0.0009*1
apicalVal=250*1
exDelay=4*1
inDelay=4*1
burstdur=100*1

strdef trailing, model
trailing = "_1_1_1_1_1"
model = "PassDend"




naCond=0.03*1
kCond=0.0009*1
apicalVal=250*1
exDelay=4*1
inDelay=4*0.25
burstdur=100*1


model= "InDelay"
trailing = "_1_1_1_-25_1"

/**************************
*Vehicle Setup        *
**************************/
strdef weight, dist, rawdatafile, extension, weightexcit, filename
strdef vglut1intensities, vgatintensities, morphologydata
dist = "Veh Dist Matrix.txt"
weight = "Veh Weight Matrix.txt"
weightexcit = "Veh Weight Matrix Excit.txt"
vglut1intensities= "Vehicle VGlut1 Ints.txt"
vgatintensities= "Vehicle VGAT Ints.txt"
morphologydata="Vehicle Morph MM.txt"
rawdatafile = "_Vehicle"
extension = ".dat"

objref Raw_Data, Raw_Data_File                  //Create a destination file for raw data
generated during run


objref VGlut1_Int_Matrix, VGlut1File                //Create source data for VGlut1 intensities
VGlut1_Int_Matrix= new Matrix()
VGlut1File= new File()
VGlut1File.ropen(vglut1intensities)
VGlut1_Int_Matrix.scanf(VGlut1File, 100, 9)

objref  VGAT_Int_Matrix, VGATFile               //Create source data for VGAT intensities
VGAT_Int_Matrix= new Matrix()
VGATFile= new File()
VGATFile.ropen(vgatintensities)
VGAT_Int_Matrix.scanf(VGATFile, 100, 9)

objref morphMatrix, morphFile                   //Create source data for arbor morphology
morphMatrix = new Matrix()
morphFile = new File()
morphFile.ropen(morphologydata)
morphMatrix.scanf(morphFile,63,37)

/**************************
*Run through each model
*for each cell trials no.
*of times         *
**************************/
```

```
for tr=0, tr=trials-1 {
    cellCounter=0
    sprint(filename, "%d%s%s%s%s", randSeed, rawdatafile, model, trailing, extension)
    print filename
    Raw_Data = new Matrix(cells+2, 5)
    Raw_Data_File = new File(filename)
    Raw_Data_File.wopen(filename)
    for i=cellStart-1, i=cellEnd-1 {
        xopen("Build Arbor Topologies Apical.hoc")
        xopen("Specify Geometry Apical.hoc")
        finitialize(-70)
        xopen("Weighted ExcitInhib Apical.hoc")
        cellCounter+=1
    }
    Raw_Data.fprint(Raw_Data_File)
    Raw_Data_File.close()
}


/**************************
*BDNF Setup       *
***************************/
strdef weight, dist, rawdatafile, extension, weightexcit, filename
strdef vglut1intensities, vgatintensities, morphologydata   //String defs to allow the same
model to be
                                //run with different source data (ie Veh or BDNF)
dist = "BDNF Dist Matrix.txt"
weight = "BDNF Weight Matrix.txt"
weightexcit = "BDNF Weight Matrix Excit.txt"
vglut1intensities= "BDNF VGlut1 Ints.txt"           //The name of the matrix for the VGlut1
intensities
vgatintensities= "BDNF VGAT Ints.txt"               //The name of the matrix for the VGAT
intensities
morphologydata="BDNF Morph MM.txt"               //The name of the matrix for the morphology
parameters (ie, no. dendrites and diameters)
rawdatafile = "_BDNF"               //The name of the raw output file
extension = ".dat"

objref Raw_Data, Raw_Data_File                  //Create a destination file for raw data
generated during run

objref VGlut1_Int_Matrix, VGlut1File                //Create source data for VGlut1 intensities
VGlut1_Int_Matrix= new Matrix()
VGlut1File= new File()
VGlut1File.ropen(vglut1intensities)
VGlut1_Int_Matrix.scanf(VGlut1File, 100, 9)

objref  VGAT_Int_Matrix, VGATFile               //Create source data for VGAT intensities
VGAT_Int_Matrix= new Matrix()
VGATFile= new File()
VGATFile.ropen(vgatintensities)
VGAT_Int_Matrix.scanf(VGATFile, 100, 9)

objref morphMatrix, morphFile                   //Create source data for arbor morphology
morphMatrix = new Matrix()
morphFile = new File()
morphFile.ropen(morphologydata)
morphMatrix.scanf(morphFile,63,37)

/**************************
*Run through each model
*for each cell trials no.
*of times          *
***************************/
for tr=0, tr=trials-1 {
    cellCounter=0
    sprint(filename, "%d%s%s%s%s", randSeed, rawdatafile, model, trailing, extension)
    print filename
    Raw_Data = new Matrix(cells+2, 5)
```

```
    Raw_Data_File = new File(filename)
    Raw_Data_File.wopen(filename)
    for i=cellStart-1, i=cellEnd-1 {
        xopen("Build Arbor Topologies Apical.hoc")
        xopen("Specify Geometry Apical.hoc")
        finitialize(-70)
        xopen("Weighted ExcitInhib Apical.hoc")
        cellCounter+=1
    }
    Raw_Data.fprint(Raw_Data_File)
    Raw_Data_File.close()
}

naCond=0.03*1
kCond=0.0009*1
apicalVal=250*1
exDelay=4*1
inDelay=4*0.5
burstdur=100*1


model= "InDelay"
trailing = "_1_1_1_-5_1"

/**************************
*Vehicle Setup        *
***************************/
strdef weight, dist, rawdatafile, extension, weightexcit, filename
strdef vglut1intensities, vgatintensities, morphologydata
dist = "Veh Dist Matrix.txt"
weight = "Veh Weight Matrix.txt"
weightexcit = "Veh Weight Matrix Excit.txt"
vglut1intensities= "Vehicle VGlut1 Ints.txt"
vgatintensities= "Vehicle VGAT Ints.txt"
morphologydata="Vehicle Morph MM.txt"
rawdatafile = "_Vehicle"
extension = ".dat"

objref Raw_Data, Raw_Data_File                    //Create a destination file for raw data
generated during run


objref VGlut1_Int_Matrix, VGlut1File                //Create source data for VGlut1 intensities
VGlut1_Int_Matrix= new Matrix()
VGlut1File= new File()
VGlut1File.ropen(vglut1intensities)
VGlut1_Int_Matrix.scanf(VGlut1File, 100, 9)

objref  VGAT_Int_Matrix, VGATFile                 //Create source data for VGAT intensities
VGAT_Int_Matrix= new Matrix()
VGATFile= new File()
VGATFile.ropen(vgatintensities)
VGAT_Int_Matrix.scanf(VGATFile, 100, 9)

objref morphMatrix, morphFile                     //Create source data for arbor morphology
morphMatrix = new Matrix()
morphFile = new File()
morphFile.ropen(morphologydata)
morphMatrix.scanf(morphFile,63,37)

/**************************
*Run through each model
*for each cell trials no.
*of times          *
***************************/

for tr=0, tr=trials-1 {
    cellCounter=0
    sprint(filename, "%d%s%s%s%s", randSeed, rawdatafile, model, trailing, extension)
```

```
    print filename
    Raw_Data = new Matrix(cells+2, 5)
    Raw_Data_File = new File(filename)
    Raw_Data_File.wopen(filename)
    for i=cellStart-1, i=cellEnd-1 {
        xopen("Build Arbor Topologies Apical.hoc")
        xopen("Specify Geometry Apical.hoc")
        finitialize(-70)
        xopen("Weighted ExcitInhib Apical.hoc")
        cellCounter+=1
    }
    Raw_Data.fprint(Raw_Data_File)
    Raw_Data_File.close()
}


/**************************
*BDNF Setup       *
***************************/
strdef weight, dist, rawdatafile, extension, weightexcit, filename
strdef vglut1intensities, vgatintensities, morphologydata   //String defs to allow the same
model to be
                                //run with different source data (ie Veh or BDNF)
dist = "BDNF Dist Matrix.txt"
weight = "BDNF Weight Matrix.txt"
weightexcit = "BDNF Weight Matrix Excit.txt"
vglut1intensities= "BDNF VGlut1 Ints.txt"           //The name of the matrix for the VGlut1
intensities
vgatintensities= "BDNF VGAT Ints.txt"               //The name of the matrix for the VGAT
intensities
morphologydata="BDNF Morph MM.txt"              //The name of the matrix for the morphology
parameters (ie, no. dendrites and diameters)
rawdatafile = "_BDNF"               //The name of the raw output file
extension = ".dat"

objref Raw_Data, Raw_Data_File                  //Create a destination file for raw data
generated during run

objref VGlut1_Int_Matrix, VGlut1File                //Create source data for VGlut1 intensities
VGlut1_Int_Matrix= new Matrix()
VGlut1File= new File()
VGlut1File.ropen(vglut1intensities)
VGlut1_Int_Matrix.scanf(VGlut1File, 100, 9)

objref  VGAT_Int_Matrix, VGATFile               //Create source data for VGAT intensities
VGAT_Int_Matrix= new Matrix()
VGATFile= new File()
VGATFile.ropen(vgatintensities)
VGAT_Int_Matrix.scanf(VGATFile, 100, 9)

objref morphMatrix, morphFile                   //Create source data for arbor morphology
morphMatrix = new Matrix()
morphFile = new File()
morphFile.ropen(morphologydata)
morphMatrix.scanf(morphFile,63,37)

/**************************
*Run through each model
*for each cell trials no.
*of times         *
***************************/
for tr=0, tr=trials-1 {
    cellCounter=0
    sprint(filename, "%d%s%s%s%s", randSeed, rawdatafile, model, trailing, extension)
    print filename
    Raw_Data = new Matrix(cells+2, 5)
    Raw_Data_File = new File(filename)
    Raw_Data_File.wopen(filename)
    for i=cellStart-1, i=cellEnd-1 {
```

```
        xopen("Build Arbor Topologies Apical.hoc")
        xopen("Specify Geometry Apical.hoc")
        finitialize(-70)
        xopen("Weighted ExcitInhib Apical.hoc")
        cellCounter+=1
    }
    Raw_Data.fprint(Raw_Data_File)
    Raw_Data_File.close()
}

naCond=0.03*1
kCond=0.0009*1
apicalVal=250*1
exDelay=4*1
inDelay=4*2
burstdur=100*1


model= "InDelay"
trailing = "_1_1_1_2_1"

/**************************
*Vehicle Setup        *
***************************/
strdef weight, dist, rawdatafile, extension, weightexcit, filename
strdef vglut1intensities, vgatintensities, morphologydata
dist = "Veh Dist Matrix.txt"
weight = "Veh Weight Matrix.txt"
weightexcit = "Veh Weight Matrix Excit.txt"
vglut1intensities= "Vehicle VGlut1 Ints.txt"
vgatintensities= "Vehicle VGAT Ints.txt"
morphologydata="Vehicle Morph MM.txt"
rawdatafile = "_Vehicle"
extension = ".dat"

objref Raw_Data, Raw_Data_File                  //Create a destination file for raw data
generated during run


objref VGlut1_Int_Matrix, VGlut1File                //Create source data for VGlut1 intensities
VGlut1_Int_Matrix= new Matrix()
VGlut1File= new File()
VGlut1File.ropen(vglut1intensities)
VGlut1_Int_Matrix.scanf(VGlut1File, 100, 9)

objref  VGAT_Int_Matrix, VGATFile               //Create source data for VGAT intensities
VGAT_Int_Matrix= new Matrix()
VGATFile= new File()
VGATFile.ropen(vgatintensities)
VGAT_Int_Matrix.scanf(VGATFile, 100, 9)

objref morphMatrix, morphFile                   //Create source data for arbor morphology
morphMatrix = new Matrix()
morphFile = new File()
morphFile.ropen(morphologydata)
morphMatrix.scanf(morphFile,63,37)

/**************************
*Run through each model
*for each cell trials no.
*of times         *
***************************/

for tr=0, tr=trials-1 {
    cellCounter=0
    sprint(filename, "%d%s%s%s%s", randSeed, rawdatafile, model, trailing, extension)
    print filename
    Raw_Data = new Matrix(cells+2, 5)
    Raw_Data_File = new File(filename)
```

```
    Raw_Data_File.wopen(filename)
    for i=cellStart-1, i=cellEnd-1 {
        xopen("Build Arbor Topologies Apical.hoc")
        xopen("Specify Geometry Apical.hoc")
        finitialize(-70)
        xopen("Weighted ExcitInhib Apical.hoc")
        cellCounter+=1
    }
    Raw_Data.fprint(Raw_Data_File)
    Raw_Data_File.close()
}


/***************************
*BDNF Setup        *
***************************/
strdef weight, dist, rawdatafile, extension, weightexcit, filename
strdef vglut1intensities, vgatintensities, morphologydata   //String defs to allow the same
model to be
                                //run with different source data (ie Veh or BDNF)
dist = "BDNF Dist Matrix.txt"
weight = "BDNF Weight Matrix.txt"
weightexcit = "BDNF Weight Matrix Excit.txt"
vglut1intensities= "BDNF VGlut1 Ints.txt"           //The name of the matrix for the VGlut1
intensities
vgatintensities= "BDNF VGAT Ints.txt"               //The name of the matrix for the VGAT
intensities
morphologydata="BDNF Morph MM.txt"              //The name of the matrix for the morphology
parameters (ie, no. dendrites and diameters)
rawdatafile = "_BDNF"               //The name of the raw output file
extension = ".dat"

objref Raw_Data, Raw_Data_File                  //Create a destination file for raw data
generated during run

objref VGlut1_Int_Matrix, VGlut1File                //Create source data for VGlut1 intensities
VGlut1_Int_Matrix= new Matrix()
VGlut1File= new File()
VGlut1File.ropen(vglut1intensities)
VGlut1_Int_Matrix.scanf(VGlut1File, 100, 9)

objref  VGAT_Int_Matrix, VGATFile               //Create source data for VGAT intensities
VGAT_Int_Matrix= new Matrix()
VGATFile= new File()
VGATFile.ropen(vgatintensities)
VGAT_Int_Matrix.scanf(VGATFile, 100, 9)

objref morphMatrix, morphFile                   //Create source data for arbor morphology
morphMatrix = new Matrix()
morphFile = new File()
morphFile.ropen(morphologydata)
morphMatrix.scanf(morphFile,63,37)

/***************************
*Run through each model
*for each cell trials no.
*of times          *
***************************/
for tr=0, tr=trials-1 {
    cellCounter=0
    sprint(filename, "%d%s%s%s%s", randSeed, rawdatafile, model, trailing, extension)
    print filename
    Raw_Data = new Matrix(cells+2, 5)
    Raw_Data_File = new File(filename)
    Raw_Data_File.wopen(filename)
    for i=cellStart-1, i=cellEnd-1 {
        xopen("Build Arbor Topologies Apical.hoc")
        xopen("Specify Geometry Apical.hoc")
        finitialize(-70)
```

```
        xopen("Weighted ExcitInhib Apical.hoc")
        cellCounter+=1
    }
    Raw_Data.fprint(Raw_Data_File)
    Raw_Data_File.close()
}

naCond=0.03*1
kCond=0.0009*1
apicalVal=250*1
exDelay=4*1
inDelay=4*4
burstdur=100*1

model= "InDelay"
trailing = "_1_1_1_4_1"

/**************************
*Vehicle Setup         *
***************************/
strdef weight, dist, rawdatafile, extension, weightexcit, filename
strdef vglut1intensities, vgatintensities, morphologydata
dist = "Veh Dist Matrix.txt"
weight = "Veh Weight Matrix.txt"
weightexcit = "Veh Weight Matrix Excit.txt"
vglut1intensities= "Vehicle VGlut1 Ints.txt"
vgatintensities= "Vehicle VGAT Ints.txt"
morphologydata="Vehicle Morph MM.txt"
rawdatafile = "_Vehicle"
extension = ".dat"

objref Raw_Data, Raw_Data_File                  //Create a destination file for raw data
generated during run


objref VGlut1_Int_Matrix, VGlut1File                //Create source data for VGlut1 intensities
VGlut1_Int_Matrix= new Matrix()
VGlut1File= new File()
VGlut1File.ropen(vglut1intensities)
VGlut1_Int_Matrix.scanf(VGlut1File, 100, 9)

objref  VGAT_Int_Matrix, VGATFile               //Create source data for VGAT intensities
VGAT_Int_Matrix= new Matrix()
VGATFile= new File()
VGATFile.ropen(vgatintensities)
VGAT_Int_Matrix.scanf(VGATFile, 100, 9)

objref morphMatrix, morphFile                   //Create source data for arbor morphology
morphMatrix = new Matrix()
morphFile = new File()
morphFile.ropen(morphologydata)
morphMatrix.scanf(morphFile,63,37)

/**************************
*Run through each model
*for each cell trials no.
*of times         *
***************************/

for tr=0, tr=trials-1 {
    cellCounter=0
    sprint(filename, "%d%s%s%s%s", randSeed, rawdatafile, model, trailing, extension)
    print filename
    Raw_Data = new Matrix(cells+2, 5)
    Raw_Data_File = new File(filename)
    Raw_Data_File.wopen(filename)
    for i=cellStart-1, i=cellEnd-1 {
        xopen("Build Arbor Topologies Apical.hoc")
        xopen("Specify Geometry Apical.hoc")
```

```
        finitialize(-70)
        xopen("Weighted ExcitInhib Apical.hoc")
        cellCounter+=1
    }
    Raw_Data.fprint(Raw_Data_File)
    Raw_Data_File.close()
}


/**************************
*BDNF Setup       *
**************************/
strdef weight, dist, rawdatafile, extension, weightexcit, filename
strdef vglut1intensities, vgatintensities, morphologydata   //String defs to allow the same
model to be
                                //run with different source data (ie Veh or BDNF)
dist = "BDNF Dist Matrix.txt"
weight = "BDNF Weight Matrix.txt"
weightexcit = "BDNF Weight Matrix Excit.txt"
vglut1intensities= "BDNF VGlut1 Ints.txt"           //The name of the matrix for the VGlut1
intensities
vgatintensities= "BDNF VGAT Ints.txt"               //The name of the matrix for the VGAT
intensities
morphologydata="BDNF Morph MM.txt"              //The name of the matrix for the morphology
parameters (ie, no. dendrites and diameters)
rawdatafile = "_BDNF"               //The name of the raw output file
extension = ".dat"

objref Raw_Data, Raw_Data_File                  //Create a destination file for raw data
generated during run

objref VGlut1_Int_Matrix, VGlut1File                //Create source data for VGlut1 intensities
VGlut1_Int_Matrix= new Matrix()
VGlut1File= new File()
VGlut1File.ropen(vglut1intensities)
VGlut1_Int_Matrix.scanf(VGlut1File, 100, 9)

objref  VGAT_Int_Matrix, VGATFile               //Create source data for VGAT intensities
VGAT_Int_Matrix= new Matrix()
VGATFile= new File()
VGATFile.ropen(vgatintensities)
VGAT_Int_Matrix.scanf(VGATFile, 100, 9)

objref morphMatrix, morphFile                   //Create source data for arbor morphology
morphMatrix = new Matrix()
morphFile = new File()
morphFile.ropen(morphologydata)
morphMatrix.scanf(morphFile,63,37)

/**************************
*Run through each model
*for each cell trials no.
*of times          *
**************************/
for tr=0, tr=trials-1 {
    cellCounter=0
    sprint(filename, "%d%s%s%s%s", randSeed, rawdatafile, model, trailing, extension)
    print filename
    Raw_Data = new Matrix(cells+2, 5)
    Raw_Data_File = new File(filename)
    Raw_Data_File.wopen(filename)
    for i=cellStart-1, i=cellEnd-1 {
        xopen("Build Arbor Topologies Apical.hoc")
        xopen("Specify Geometry Apical.hoc")
        finitialize(-70)
        xopen("Weighted ExcitInhib Apical.hoc")
        cellCounter+=1
    }
    Raw_Data.fprint(Raw_Data_File)
```

```
    Raw_Data_File.close()
}



/*
Run_IntWindow.hoc

This file modulates the length of the integration window
*/

/**************************
*Global Setup          *
***************************/
getcwd()
chdir("/cygdrive/c/Users/Nick/Desktop/Neuron Model/11")
trials=1                        //1 trial equals 1 run through all cells specified
cellStart=1                     //This can be used to analyze a subset of cells
cellEnd=63                      //Example, to analyze cells 1-10: cellStart=1, cellEnd=10
cells =(cellEnd-cellStart)+1                //This generates the total number of cells that
will be analyzed                    //This is the length of time where synapses can be activated
objref randNum                      //This generates the random number for the model
randNum = new Random(randSeed)


naCond=0.03*1
kCond=0.0009*1
apicalVal=250*1
exDelay=4*1
inDelay=4*1
burstdur=100*1

strdef trailing, model
trailing = "_1_1_1_1_1"
model = "PassDend"




naCond=0.03*1
kCond=0.0009*1
apicalVal=250*1
exDelay=4*1
inDelay=4*1
burstdur=100*0.25


model= "IntWindow"
trailing = "_1_1_1_1_-25"

/**************************
*Vehicle Setup         *
***************************/
strdef weight, dist, rawdatafile, extension, weightexcit, filename
strdef vglut1intensities, vgatintensities, morphologydata
dist = "Veh Dist Matrix.txt"
weight = "Veh Weight Matrix.txt"
weightexcit = "Veh Weight Matrix Excit.txt"
vglut1intensities= "Vehicle VGlut1 Ints.txt"
vgatintensities= "Vehicle VGAT Ints.txt"
morphologydata="Vehicle Morph MM.txt"
rawdatafile = "_Vehicle"
extension = ".dat"

objref Raw_Data, Raw_Data_File                  //Create a destination file for raw data
generated during run


objref VGlut1_Int_Matrix, VGlut1File                //Create source data for VGlut1 intensities
```

```
VGlut1_Int_Matrix= new Matrix()
VGlut1File= new File()
VGlut1File.ropen(vglut1intensities)
VGlut1_Int_Matrix.scanf(VGlut1File, 100, 9)

objref  VGAT_Int_Matrix, VGATFile                //Create source data for VGAT intensities
VGAT_Int_Matrix= new Matrix()
VGATFile= new File()
VGATFile.ropen(vgatintensities)
VGAT_Int_Matrix.scanf(VGATFile, 100, 9)

objref morphMatrix, morphFile                    //Create source data for arbor morphology
morphMatrix = new Matrix()
morphFile = new File()
morphFile.ropen(morphologydata)
morphMatrix.scanf(morphFile,63,37)

/**************************
*Run through each model
*for each cell trials no.
*of times          *
***************************/

for tr=0, tr=trials-1 {
    cellCounter=0
    sprint(filename, "%d%s%s%s%s", randSeed, rawdatafile, model, trailing, extension)
    print filename
    Raw_Data = new Matrix(cells+2, 5)
    Raw_Data_File = new File(filename)
    Raw_Data_File.wopen(filename)
    for i=cellStart-1, i=cellEnd-1 {
        xopen("Build Arbor Topologies Apical.hoc")
        xopen("Specify Geometry Apical.hoc")
        finitialize(-70)
        xopen("Weighted ExcitInhib Apical.hoc")
        cellCounter+=1
    }
    Raw_Data.fprint(Raw_Data_File)
    Raw_Data_File.close()
}


/**************************
*BDNF Setup       *
***************************/
strdef weight, dist, rawdatafile, extension, weightexcit, filename
strdef vglut1intensities, vgatintensities, morphologydata   //String defs to allow the same
model to be
                                //run with different source data (ie Veh or BDNF)
dist = "BDNF Dist Matrix.txt"
weight = "BDNF Weight Matrix.txt"
weightexcit = "BDNF Weight Matrix Excit.txt"
vglut1intensities= "BDNF VGlut1 Ints.txt"          //The name of the matrix for the VGlut1
intensities
vgatintensities= "BDNF VGAT Ints.txt"              //The name of the matrix for the VGAT
intensities
morphologydata="BDNF Morph MM.txt"              //The name of the matrix for the morphology
parameters (ie, no. dendrites and diameters)
rawdatafile = "_BDNF"               //The name of the raw output file
extension = ".dat"

objref Raw_Data, Raw_Data_File                  //Create a destination file for raw data
generated during run

objref VGlut1_Int_Matrix, VGlut1File                //Create source data for VGlut1 intensities
VGlut1_Int_Matrix= new Matrix()
VGlut1File= new File()
VGlut1File.ropen(vglut1intensities)
VGlut1_Int_Matrix.scanf(VGlut1File, 100, 9)
```

```
objref  VGAT_Int_Matrix, VGATFile                //Create source data for VGAT intensities
VGAT_Int_Matrix= new Matrix()
VGATFile= new File()
VGATFile.ropen(vgatintensities)
VGAT_Int_Matrix.scanf(VGATFile, 100, 9)

objref morphMatrix, morphFile                    //Create source data for arbor morphology
morphMatrix = new Matrix()
morphFile = new File()
morphFile.ropen(morphologydata)
morphMatrix.scanf(morphFile,63,37)

/**************************
*Run through each model
*for each cell trials no.
*of times         *
***************************/
for tr=0, tr=trials-1 {
    cellCounter=0
    sprint(filename, "%d%s%s%s%s", randSeed, rawdatafile, model, trailing, extension)
    print filename
    Raw_Data = new Matrix(cells+2, 5)
    Raw_Data_File = new File(filename)
    Raw_Data_File.wopen(filename)
    for i=cellStart-1, i=cellEnd-1 {
        xopen("Build Arbor Topologies Apical.hoc")
        xopen("Specify Geometry Apical.hoc")
        finitialize(-70)
        xopen("Weighted ExcitInhib Apical.hoc")
        cellCounter+=1
    }
    Raw_Data.fprint(Raw_Data_File)
    Raw_Data_File.close()
}

naCond=0.03*1
kCond=0.0009*1
apicalVal=250*1
exDelay=4*1
inDelay=4*1
burstdur=100*0.5


model= "IntWindow"
trailing = "_1_1_1_1_-5"

/**************************
*Vehicle Setup        *
***************************/
strdef weight, dist, rawdatafile, extension, weightexcit, filename
strdef vglut1intensities, vgatintensities, morphologydata
dist = "Veh Dist Matrix.txt"
weight = "Veh Weight Matrix.txt"
weightexcit = "Veh Weight Matrix Excit.txt"
vglut1intensities= "Vehicle VGlut1 Ints.txt"
vgatintensities= "Vehicle VGAT Ints.txt"
morphologydata="Vehicle Morph MM.txt"
rawdatafile = "_Vehicle"
extension = ".dat"

objref Raw_Data, Raw_Data_File                   //Create a destination file for raw data
generated during run


objref VGlut1_Int_Matrix, VGlut1File                 //Create source data for VGlut1 intensities
VGlut1_Int_Matrix= new Matrix()
VGlut1File= new File()
VGlut1File.ropen(vglut1intensities)
```

```
VGlut1_Int_Matrix.scanf(VGlut1File, 100, 9)

objref  VGAT_Int_Matrix, VGATFile                //Create source data for VGAT intensities
VGAT_Int_Matrix= new Matrix()
VGATFile= new File()
VGATFile.ropen(vgatintensities)
VGAT_Int_Matrix.scanf(VGATFile, 100, 9)

objref morphMatrix, morphFile                    //Create source data for arbor morphology
morphMatrix = new Matrix()
morphFile = new File()
morphFile.ropen(morphologydata)
morphMatrix.scanf(morphFile,63,37)

/***************************
*Run through each model
*for each cell trials no.
*of times          *
****************************/

for tr=0, tr=trials-1 {
    cellCounter=0
    sprint(filename, "%d%s%s%s%s", randSeed, rawdatafile, model, trailing, extension)
    print filename
    Raw_Data = new Matrix(cells+2, 5)
    Raw_Data_File = new File(filename)
    Raw_Data_File.wopen(filename)
    for i=cellStart-1, i=cellEnd-1 {
        xopen("Build Arbor Topologies Apical.hoc")
        xopen("Specify Geometry Apical.hoc")
        finitialize(-70)
        xopen("Weighted ExcitInhib Apical.hoc")
        cellCounter+=1
    }
    Raw_Data.fprint(Raw_Data_File)
    Raw_Data_File.close()
}


/***************************
*BDNF Setup        *
****************************/
strdef weight, dist, rawdatafile, extension, weightexcit, filename
strdef vglut1intensities, vgatintensities, morphologydata   //String defs to allow the same
model to be
                                //run with different source data (ie Veh or BDNF)
dist = "BDNF Dist Matrix.txt"
weight = "BDNF Weight Matrix.txt"
weightexcit = "BDNF Weight Matrix Excit.txt"
vglut1intensities= "BDNF VGlut1 Ints.txt"           //The name of the matrix for the VGlut1
intensities
vgatintensities= "BDNF VGAT Ints.txt"               //The name of the matrix for the VGAT
intensities
morphologydata="BDNF Morph MM.txt"              //The name of the matrix for the morphology
parameters (ie, no. dendrites and diameters)
rawdatafile = "_BDNF"                //The name of the raw output file
extension = ".dat"

objref Raw_Data, Raw_Data_File                   //Create a destination file for raw data
generated during run

objref VGlut1_Int_Matrix, VGlut1File                 //Create source data for VGlut1 intensities
VGlut1_Int_Matrix= new Matrix()
VGlut1File= new File()
VGlut1File.ropen(vglut1intensities)
VGlut1_Int_Matrix.scanf(VGlut1File, 100, 9)

objref  VGAT_Int_Matrix, VGATFile                //Create source data for VGAT intensities
VGAT_Int_Matrix= new Matrix()
```

```
VGATFile= new File()
VGATFile.ropen(vgatintensities)
VGAT_Int_Matrix.scanf(VGATFile, 100, 9)

objref morphMatrix, morphFile                    //Create source data for arbor morphology
morphMatrix = new Matrix()
morphFile = new File()
morphFile.ropen(morphologydata)
morphMatrix.scanf(morphFile,63,37)

/**************************
*Run through each model
*for each cell trials no.
*of times          *
***************************/
for tr=0, tr=trials-1 {
    cellCounter=0
    sprint(filename, "%d%s%s%s%s", randSeed, rawdatafile, model, trailing, extension)
    print filename
    Raw_Data = new Matrix(cells+2, 5)
    Raw_Data_File = new File(filename)
    Raw_Data_File.wopen(filename)
    for i=cellStart-1, i=cellEnd-1 {
        xopen("Build Arbor Topologies Apical.hoc")
        xopen("Specify Geometry Apical.hoc")
        finitialize(-70)
        xopen("Weighted ExcitInhib Apical.hoc")
        cellCounter+=1
    }
    Raw_Data.fprint(Raw_Data_File)
    Raw_Data_File.close()
}

naCond=0.03*1
kCond=0.0009*1
apicalVal=250*1
exDelay=4*1
inDelay=4*1
burstdur=100*2


model= "IntWindow"
trailing = "_1_1_1_1_2"

/**************************
*Vehicle Setup        *
***************************/
strdef weight, dist, rawdatafile, extension, weightexcit, filename
strdef vglut1intensities, vgatintensities, morphologydata
dist = "Veh Dist Matrix.txt"
weight = "Veh Weight Matrix.txt"
weightexcit = "Veh Weight Matrix Excit.txt"
vglut1intensities= "Vehicle VGlut1 Ints.txt"
vgatintensities= "Vehicle VGAT Ints.txt"
morphologydata="Vehicle Morph MM.txt"
rawdatafile = "_Vehicle"
extension = ".dat"

objref Raw_Data, Raw_Data_File                   //Create a destination file for raw data
generated during run


objref VGlut1_Int_Matrix, VGlut1File                 //Create source data for VGlut1 intensities
VGlut1_Int_Matrix= new Matrix()
VGlut1File= new File()
VGlut1File.ropen(vglut1intensities)
VGlut1_Int_Matrix.scanf(VGlut1File, 100, 9)

objref  VGAT_Int_Matrix, VGATFile                //Create source data for VGAT intensities
```

```
VGAT_Int_Matrix= new Matrix()
VGATFile= new File()
VGATFile.ropen(vgatintensities)
VGAT_Int_Matrix.scanf(VGATFile, 100, 9)

objref morphMatrix, morphFile                    //Create source data for arbor morphology
morphMatrix = new Matrix()
morphFile = new File()
morphFile.ropen(morphologydata)
morphMatrix.scanf(morphFile,63,37)

/**************************
*Run through each model
*for each cell trials no.
*of times         *
***************************/

for tr=0, tr=trials-1 {
    cellCounter=0
    sprint(filename, "%d%s%s%s%s", randSeed, rawdatafile, model, trailing, extension)
    print filename
    Raw_Data = new Matrix(cells+2, 5)
    Raw_Data_File = new File(filename)
    Raw_Data_File.wopen(filename)
    for i=cellStart-1, i=cellEnd-1 {
        xopen("Build Arbor Topologies Apical.hoc")
        xopen("Specify Geometry Apical.hoc")
        finitialize(-70)
        xopen("Weighted ExcitInhib Apical.hoc")
        cellCounter+=1
    }
    Raw_Data.fprint(Raw_Data_File)
    Raw_Data_File.close()
}


/**************************
*BDNF Setup       *
***************************/
strdef weight, dist, rawdatafile, extension, weightexcit, filename
strdef vglut1intensities, vgatintensities, morphologydata   //String defs to allow the same
model to be
                                //run with different source data (ie Veh or BDNF)
dist = "BDNF Dist Matrix.txt"
weight = "BDNF Weight Matrix.txt"
weightexcit = "BDNF Weight Matrix Excit.txt"
vglut1intensities= "BDNF VGlut1 Ints.txt"           //The name of the matrix for the VGlut1
intensities
vgatintensities= "BDNF VGAT Ints.txt"               //The name of the matrix for the VGAT
intensities
morphologydata="BDNF Morph MM.txt"              //The name of the matrix for the morphology
parameters (ie, no. dendrites and diameters)
rawdatafile = "_BDNF"               //The name of the raw output file
extension = ".dat"

objref Raw_Data, Raw_Data_File                   //Create a destination file for raw data
generated during run

objref VGlut1_Int_Matrix, VGlut1File                //Create source data for VGlut1 intensities
VGlut1_Int_Matrix= new Matrix()
VGlut1File= new File()
VGlut1File.ropen(vglut1intensities)
VGlut1_Int_Matrix.scanf(VGlut1File, 100, 9)

objref  VGAT_Int_Matrix, VGATFile                //Create source data for VGAT intensities
VGAT_Int_Matrix= new Matrix()
VGATFile= new File()
VGATFile.ropen(vgatintensities)
VGAT_Int_Matrix.scanf(VGATFile, 100, 9)
```

```
objref morphMatrix, morphFile                    //Create source data for arbor morphology
morphMatrix = new Matrix()
morphFile = new File()
morphFile.ropen(morphologydata)
morphMatrix.scanf(morphFile,63,37)

/**************************
*Run through each model
*for each cell trials no.
*of times         *
***************************/
for tr=0, tr=trials-1 {
    cellCounter=0
    sprint(filename, "%d%s%s%s%s", randSeed, rawdatafile, model, trailing, extension)
    print filename
    Raw_Data = new Matrix(cells+2, 5)
    Raw_Data_File = new File(filename)
    Raw_Data_File.wopen(filename)
    for i=cellStart-1, i=cellEnd-1 {
        xopen("Build Arbor Topologies Apical.hoc")
        xopen("Specify Geometry Apical.hoc")
        finitialize(-70)
        xopen("Weighted ExcitInhib Apical.hoc")
        cellCounter+=1
    }
    Raw_Data.fprint(Raw_Data_File)
    Raw_Data_File.close()
}

naCond=0.03*1
kCond=0.0009*1
apicalVal=250*1
exDelay=4*1
inDelay=4*1
burstdur=100*4

model= "IntWindow"
trailing = "_1_1_1_1_4"

/**************************
*Vehicle Setup        *
***************************/
strdef weight, dist, rawdatafile, extension, weightexcit, filename
strdef vglut1intensities, vgatintensities, morphologydata
dist = "Veh Dist Matrix.txt"
weight = "Veh Weight Matrix.txt"
weightexcit = "Veh Weight Matrix Excit.txt"
vglut1intensities= "Vehicle VGlut1 Ints.txt"
vgatintensities= "Vehicle VGAT Ints.txt"
morphologydata="Vehicle Morph MM.txt"
rawdatafile = "_Vehicle"
extension = ".dat"

objref Raw_Data, Raw_Data_File                  //Create a destination file for raw data
generated during run


objref VGlut1_Int_Matrix, VGlut1File                //Create source data for VGlut1 intensities
VGlut1_Int_Matrix= new Matrix()
VGlut1File= new File()
VGlut1File.ropen(vglut1intensities)
VGlut1_Int_Matrix.scanf(VGlut1File, 100, 9)

objref  VGAT_Int_Matrix, VGATFile               //Create source data for VGAT intensities
VGAT_Int_Matrix= new Matrix()
VGATFile= new File()
VGATFile.ropen(vgatintensities)
VGAT_Int_Matrix.scanf(VGATFile, 100, 9)
```

```
objref morphMatrix, morphFile                    //Create source data for arbor morphology
morphMatrix = new Matrix()
morphFile = new File()
morphFile.ropen(morphologydata)
morphMatrix.scanf(morphFile,63,37)

/**************************
*Run through each model
*for each cell trials no.
*of times          *
***************************/

for tr=0, tr=trials-1 {
    cellCounter=0
    sprint(filename, "%d%s%s%s%s", randSeed, rawdatafile, model, trailing, extension)
    print filename
    Raw_Data = new Matrix(cells+2, 5)
    Raw_Data_File = new File(filename)
    Raw_Data_File.wopen(filename)
    for i=cellStart-1, i=cellEnd-1 {
        xopen("Build Arbor Topologies Apical.hoc")
        xopen("Specify Geometry Apical.hoc")
        finitialize(-70)
        xopen("Weighted ExcitInhib Apical.hoc")
        cellCounter+=1
    }
    Raw_Data.fprint(Raw_Data_File)
    Raw_Data_File.close()
}


/**************************
*BDNF Setup        *
***************************/
strdef weight, dist, rawdatafile, extension, weightexcit, filename
strdef vglut1intensities, vgatintensities, morphologydata   //String defs to allow the same
model to be
                                 //run with different source data (ie Veh or BDNF)
dist = "BDNF Dist Matrix.txt"
weight = "BDNF Weight Matrix.txt"
weightexcit = "BDNF Weight Matrix Excit.txt"
vglut1intensities= "BDNF VGlut1 Ints.txt"           //The name of the matrix for the VGlut1
intensities
vgatintensities= "BDNF VGAT Ints.txt"               //The name of the matrix for the VGAT
intensities
morphologydata="BDNF Morph MM.txt"              //The name of the matrix for the morphology
parameters (ie, no. dendrites and diameters)
rawdatafile = "_BDNF"               //The name of the raw output file
extension = ".dat"

objref Raw_Data, Raw_Data_File                   //Create a destination file for raw data
generated during run

objref VGlut1_Int_Matrix, VGlut1File                //Create source data for VGlut1 intensities
VGlut1_Int_Matrix= new Matrix()
VGlut1File= new File()
VGlut1File.ropen(vglut1intensities)
VGlut1_Int_Matrix.scanf(VGlut1File, 100, 9)

objref  VGAT_Int_Matrix, VGATFile                //Create source data for VGAT intensities
VGAT_Int_Matrix= new Matrix()
VGATFile= new File()
VGATFile.ropen(vgatintensities)
VGAT_Int_Matrix.scanf(VGATFile, 100, 9)

objref morphMatrix, morphFile                    //Create source data for arbor morphology
morphMatrix = new Matrix()
morphFile = new File()
```

```
morphFile.ropen(morphologydata)
morphMatrix.scanf(morphFile,63,37)

/**************************
*Run through each model
*for each cell trials no.
*of times          *
***************************/
for tr=0, tr=trials-1 {
    cellCounter=0
    sprint(filename, "%d%s%s%s%s", randSeed, rawdatafile, model, trailing, extension)
    print filename
    Raw_Data = new Matrix(cells+2, 5)
    Raw_Data_File = new File(filename)
    Raw_Data_File.wopen(filename)
    for i=cellStart-1, i=cellEnd-1 {
        xopen("Build Arbor Topologies Apical.hoc")
        xopen("Specify Geometry Apical.hoc")
        finitialize(-70)
        xopen("Weighted ExcitInhib Apical.hoc")
        cellCounter+=1
    }
    Raw_Data.fprint(Raw_Data_File)
    Raw_Data_File.close()
}


/*
Run_PassDend.hoc

This file modulates the excitability of the dendritic membrane
*/

/**************************
*Global Setup          *
***************************/
getcwd()
chdir("/cygdrive/c/Users/Nick/Desktop/Neuron Model/11")
trials=1                        //1 trial equals 1 run through all cells specified
cellStart=1                     //This can be used to analyze a subset of cells
cellEnd=63                      //Example, to analyze cells 1-10: cellStart=1, cellEnd=10
cells =(cellEnd-cellStart)+1                //This generates the total number of cells that
will be analyzed                    //This is the length of time where synapses can be activated
objref randNum                      //This generates the random number for the model
randNum = new Random(randSeed)


naCond=0.03*1
kCond=0.0009*1
apicalVal=250*1
exDelay=4*1
inDelay=4*1
burstdur=100*1

strdef trailing, model
trailing = "_1_1_1_1_1"
model = "PassDend"


/**************************
*Vehicle Setup         *
***************************/
strdef weight, dist, rawdatafile, extension, weightexcit, filename
strdef vglut1intensities, vgatintensities, morphologydata
dist = "Veh Dist Matrix.txt"
weight = "Veh Weight Matrix.txt"
weightexcit = "Veh Weight Matrix Excit.txt"
vglut1intensities= "Vehicle VGlut1 Ints.txt"
vgatintensities= "Vehicle VGAT Ints.txt"
```

```
morphologydata="Vehicle Morph MM.txt"
rawdatafile = "_Vehicle"
extension = ".dat"

objref Raw_Data, Raw_Data_File                    //Create a destination file for raw data
generated during run


objref VGlut1_Int_Matrix, VGlut1File                //Create source data for VGlut1 intensities
VGlut1_Int_Matrix= new Matrix()
VGlut1File= new File()
VGlut1File.ropen(vglut1intensities)
VGlut1_Int_Matrix.scanf(VGlut1File, 100, 9)

objref  VGAT_Int_Matrix, VGATFile                 //Create source data for VGAT intensities
VGAT_Int_Matrix= new Matrix()
VGATFile= new File()
VGATFile.ropen(vgatintensities)
VGAT_Int_Matrix.scanf(VGATFile, 100, 9)

objref morphMatrix, morphFile                     //Create source data for arbor morphology
morphMatrix = new Matrix()
morphFile = new File()
morphFile.ropen(morphologydata)
morphMatrix.scanf(morphFile,63,37)

/**************************
*Run through each model
*for each cell trials no.
*of times          *
***************************/

for tr=0, tr=trials-1 {
    cellCounter=0
    sprint(filename, "%d%s%s%s%s", randSeed, rawdatafile, model, trailing, extension)
    print filename
    Raw_Data = new Matrix(cells+2, 5)
    Raw_Data_File = new File(filename)
    Raw_Data_File.wopen(filename)
    for i=cellStart-1, i=cellEnd-1 {
        xopen("Build Arbor Topologies Apical.hoc")
        xopen("Specify Geometry Apical.hoc")
        finitialize(-70)
        xopen("Weighted ExcitInhib Apical.hoc")
        cellCounter+=1
    }
    Raw_Data.fprint(Raw_Data_File)
    Raw_Data_File.close()
}


/**************************
*BDNF Setup       *
***************************/
strdef weight, dist, rawdatafile, extension, weightexcit, filename
strdef vglut1intensities, vgatintensities, morphologydata   //String defs to allow the same
model to be
                                //run with different source data (ie Veh or BDNF)
dist = "BDNF Dist Matrix.txt"
weight = "BDNF Weight Matrix.txt"
weightexcit = "BDNF Weight Matrix Excit.txt"
vglut1intensities= "BDNF VGlut1 Ints.txt"           //The name of the matrix for the VGlut1
intensities
vgatintensities= "BDNF VGAT Ints.txt"               //The name of the matrix for the VGAT
intensities
morphologydata="BDNF Morph MM.txt"                //The name of the matrix for the morphology
parameters (ie, no. dendrites and diameters)
rawdatafile = "_BDNF"                  //The name of the raw output file
extension = ".dat"
```

```
objref Raw_Data, Raw_Data_File                    //Create a destination file for raw data
generated during run

objref VGlut1_Int_Matrix, VGlut1File                //Create source data for VGlut1 intensities
VGlut1_Int_Matrix= new Matrix()
VGlut1File= new File()
VGlut1File.ropen(vglut1intensities)
VGlut1_Int_Matrix.scanf(VGlut1File, 100, 9)

objref  VGAT_Int_Matrix, VGATFile                 //Create source data for VGAT intensities
VGAT_Int_Matrix= new Matrix()
VGATFile= new File()
VGATFile.ropen(vgatintensities)
VGAT_Int_Matrix.scanf(VGATFile, 100, 9)

objref morphMatrix, morphFile                     //Create source data for arbor morphology
morphMatrix = new Matrix()
morphFile = new File()
morphFile.ropen(morphologydata)
morphMatrix.scanf(morphFile,63,37)

/**************************
*Run through each model
*for each cell trials no.
*of times         *
***************************/
for tr=0, tr=trials-1 {
    cellCounter=0
    sprint(filename, "%d%s%s%s%s", randSeed, rawdatafile, model, trailing, extension)
    print filename
    Raw_Data = new Matrix(cells+2, 5)
    Raw_Data_File = new File(filename)
    Raw_Data_File.wopen(filename)
    for i=cellStart-1, i=cellEnd-1 {
        xopen("Build Arbor Topologies Apical.hoc")
        xopen("Specify Geometry Apical.hoc")
        finitialize(-70)
        xopen("Weighted ExcitInhib Apical.hoc")
        cellCounter+=1
    }
    Raw_Data.fprint(Raw_Data_File)
    Raw_Data_File.close()
}

naCond=0.03*0.25
kCond=0.0009*0.25
apicalVal=250*1
exDelay=4*1
inDelay=4*1
burstdur=100*1


model= "PassDend"
trailing = "_-25_1_1_1_1"

/**************************
*Vehicle Setup        *
***************************/
strdef weight, dist, rawdatafile, extension, weightexcit, filename
strdef vglut1intensities, vgatintensities, morphologydata
dist = "Veh Dist Matrix.txt"
weight = "Veh Weight Matrix.txt"
weightexcit = "Veh Weight Matrix Excit.txt"
vglut1intensities= "Vehicle VGlut1 Ints.txt"
vgatintensities= "Vehicle VGAT Ints.txt"
morphologydata="Vehicle Morph MM.txt"
rawdatafile = "_Vehicle"
extension = ".dat"
```

```
objref Raw_Data, Raw_Data_File                  //Create a destination file for raw data
generated during run


objref VGlut1_Int_Matrix, VGlut1File                //Create source data for VGlut1 intensities
VGlut1_Int_Matrix= new Matrix()
VGlut1File= new File()
VGlut1File.ropen(vglut1intensities)
VGlut1_Int_Matrix.scanf(VGlut1File, 100, 9)

objref  VGAT_Int_Matrix, VGATFile               //Create source data for VGAT intensities
VGAT_Int_Matrix= new Matrix()
VGATFile= new File()
VGATFile.ropen(vgatintensities)
VGAT_Int_Matrix.scanf(VGATFile, 100, 9)

objref morphMatrix, morphFile                   //Create source data for arbor morphology
morphMatrix = new Matrix()
morphFile = new File()
morphFile.ropen(morphologydata)
morphMatrix.scanf(morphFile,63,37)

/**************************
*Run through each model
*for each cell trials no.
*of times          *
***************************/

for tr=0, tr=trials-1 {
    cellCounter=0
    sprint(filename, "%d%s%s%s%s", randSeed, rawdatafile, model, trailing, extension)
    print filename
    Raw_Data = new Matrix(cells+2, 5)
    Raw_Data_File = new File(filename)
    Raw_Data_File.wopen(filename)
    for i=cellStart-1, i=cellEnd-1 {
        xopen("Build Arbor Topologies Apical.hoc")
        xopen("Specify Geometry Apical.hoc")
        finitialize(-70)
        xopen("Weighted ExcitInhib Apical.hoc")
        cellCounter+=1
    }
    Raw_Data.fprint(Raw_Data_File)
    Raw_Data_File.close()
}


/**************************
*BDNF Setup        *
***************************/
strdef weight, dist, rawdatafile, extension, weightexcit, filename
strdef vglut1intensities, vgatintensities, morphologydata   //String defs to allow the same
model to be
                                //run with different source data (ie Veh or BDNF)
dist = "BDNF Dist Matrix.txt"
weight = "BDNF Weight Matrix.txt"
weightexcit = "BDNF Weight Matrix Excit.txt"
vglut1intensities= "BDNF VGlut1 Ints.txt"           //The name of the matrix for the VGlut1
intensities
vgatintensities= "BDNF VGAT Ints.txt"               //The name of the matrix for the VGAT
intensities
morphologydata="BDNF Morph MM.txt"              //The name of the matrix for the morphology
parameters (ie, no. dendrites and diameters)
rawdatafile = "_BDNF"               //The name of the raw output file
extension = ".dat"

objref Raw_Data, Raw_Data_File                  //Create a destination file for raw data
generated during run
```

```
objref VGlut1_Int_Matrix, VGlut1File                //Create source data for VGlut1 intensities
VGlut1_Int_Matrix= new Matrix()
VGlut1File= new File()
VGlut1File.ropen(vglut1intensities)
VGlut1_Int_Matrix.scanf(VGlut1File, 100, 9)

objref  VGAT_Int_Matrix, VGATFile               //Create source data for VGAT intensities
VGAT_Int_Matrix= new Matrix()
VGATFile= new File()
VGATFile.ropen(vgatintensities)
VGAT_Int_Matrix.scanf(VGATFile, 100, 9)

objref morphMatrix, morphFile                   //Create source data for arbor morphology
morphMatrix = new Matrix()
morphFile = new File()
morphFile.ropen(morphologydata)
morphMatrix.scanf(morphFile,63,37)

/**************************
*Run through each model
*for each cell trials no.
*of times          *
***************************/
for tr=0, tr=trials-1 {
    cellCounter=0
    sprint(filename, "%d%s%s%s%s", randSeed, rawdatafile, model, trailing, extension)
    print filename
    Raw_Data = new Matrix(cells+2, 5)
    Raw_Data_File = new File(filename)
    Raw_Data_File.wopen(filename)
    for i=cellStart-1, i=cellEnd-1 {
        xopen("Build Arbor Topologies Apical.hoc")
        xopen("Specify Geometry Apical.hoc")
        finitialize(-70)
        xopen("Weighted ExcitInhib Apical.hoc")
        cellCounter+=1
    }
    Raw_Data.fprint(Raw_Data_File)
    Raw_Data_File.close()
}

naCond=0.03*0.5
kCond=0.0009*0.5
apicalVal=250*1
exDelay=4*1
inDelay=4*1
burstdur=100*1


model= "PassDend"
trailing = "_-5_1_1_1_1"

/**************************
*Vehicle Setup        *
***************************/
strdef weight, dist, rawdatafile, extension, weightexcit, filename
strdef vglut1intensities, vgatintensities, morphologydata
dist = "Veh Dist Matrix.txt"
weight = "Veh Weight Matrix.txt"
weightexcit = "Veh Weight Matrix Excit.txt"
vglut1intensities= "Vehicle VGlut1 Ints.txt"
vgatintensities= "Vehicle VGAT Ints.txt"
morphologydata="Vehicle Morph MM.txt"
rawdatafile = "_Vehicle"
extension = ".dat"

objref Raw_Data, Raw_Data_File                  //Create a destination file for raw data
generated during run
```

```
objref VGlut1_Int_Matrix, VGlut1File                //Create source data for VGlut1 intensities
VGlut1_Int_Matrix= new Matrix()
VGlut1File= new File()
VGlut1File.ropen(vglut1intensities)
VGlut1_Int_Matrix.scanf(VGlut1File, 100, 9)

objref  VGAT_Int_Matrix, VGATFile               //Create source data for VGAT intensities
VGAT_Int_Matrix= new Matrix()
VGATFile= new File()
VGATFile.ropen(vgatintensities)
VGAT_Int_Matrix.scanf(VGATFile, 100, 9)

objref morphMatrix, morphFile                   //Create source data for arbor morphology
morphMatrix = new Matrix()
morphFile = new File()
morphFile.ropen(morphologydata)
morphMatrix.scanf(morphFile,63,37)

/**************************
*Run through each model
*for each cell trials no.
*of times         *
***************************/

for tr=0, tr=trials-1 {
    cellCounter=0
    sprint(filename, "%d%s%s%s%s", randSeed, rawdatafile, model, trailing, extension)
    print filename
    Raw_Data = new Matrix(cells+2, 5)
    Raw_Data_File = new File(filename)
    Raw_Data_File.wopen(filename)
    for i=cellStart-1, i=cellEnd-1 {
        xopen("Build Arbor Topologies Apical.hoc")
        xopen("Specify Geometry Apical.hoc")
        finitialize(-70)
        xopen("Weighted ExcitInhib Apical.hoc")
        cellCounter+=1
    }
    Raw_Data.fprint(Raw_Data_File)
    Raw_Data_File.close()
}


/**************************
*BDNF Setup       *
***************************/
strdef weight, dist, rawdatafile, extension, weightexcit, filename
strdef vglut1intensities, vgatintensities, morphologydata   //String defs to allow the same
model to be
                                //run with different source data (ie Veh or BDNF)
dist = "BDNF Dist Matrix.txt"
weight = "BDNF Weight Matrix.txt"
weightexcit = "BDNF Weight Matrix Excit.txt"
vglut1intensities= "BDNF VGlut1 Ints.txt"           //The name of the matrix for the VGlut1
intensities
vgatintensities= "BDNF VGAT Ints.txt"               //The name of the matrix for the VGAT
intensities
morphologydata="BDNF Morph MM.txt"              //The name of the matrix for the morphology
parameters (ie, no. dendrites and diameters)
rawdatafile = "_BDNF"               //The name of the raw output file
extension = ".dat"

objref Raw_Data, Raw_Data_File                  //Create a destination file for raw data
generated during run

objref VGlut1_Int_Matrix, VGlut1File                //Create source data for VGlut1 intensities
VGlut1_Int_Matrix= new Matrix()
```

```
VGlut1File= new File()
VGlut1File.ropen(vglut1intensities)
VGlut1_Int_Matrix.scanf(VGlut1File, 100, 9)

objref  VGAT_Int_Matrix, VGATFile               //Create source data for VGAT intensities
VGAT_Int_Matrix= new Matrix()
VGATFile= new File()
VGATFile.ropen(vgatintensities)
VGAT_Int_Matrix.scanf(VGATFile, 100, 9)

objref morphMatrix, morphFile                   //Create source data for arbor morphology
morphMatrix = new Matrix()
morphFile = new File()
morphFile.ropen(morphologydata)
morphMatrix.scanf(morphFile,63,37)

/***************************
*Run through each model
*for each cell trials no.
*of times          *
***************************/
for tr=0, tr=trials-1 {
    cellCounter=0
    sprint(filename, "%d%s%s%s%s", randSeed, rawdatafile, model, trailing, extension)
    print filename
    Raw_Data = new Matrix(cells+2, 5)
    Raw_Data_File = new File(filename)
    Raw_Data_File.wopen(filename)
    for i=cellStart-1, i=cellEnd-1 {
        xopen("Build Arbor Topologies Apical.hoc")
        xopen("Specify Geometry Apical.hoc")
        finitialize(-70)
        xopen("Weighted ExcitInhib Apical.hoc")
        cellCounter+=1
    }
    Raw_Data.fprint(Raw_Data_File)
    Raw_Data_File.close()
}

naCond=0.03*2
kCond=0.0009*2
apicalVal=250*1
exDelay=4*1
inDelay=4*1
burstdur=100*1


model= "PassDend"
trailing = "_2_1_1_1_1"

/***************************
*Vehicle Setup        *
***************************/
strdef weight, dist, rawdatafile, extension, weightexcit, filename
strdef vglut1intensities, vgatintensities, morphologydata
dist = "Veh Dist Matrix.txt"
weight = "Veh Weight Matrix.txt"
weightexcit = "Veh Weight Matrix Excit.txt"
vglut1intensities= "Vehicle VGlut1 Ints.txt"
vgatintensities= "Vehicle VGAT Ints.txt"
morphologydata="Vehicle Morph MM.txt"
rawdatafile = "_Vehicle"
extension = ".dat"

objref Raw_Data, Raw_Data_File                  //Create a destination file for raw data
generated during run


objref VGlut1_Int_Matrix, VGlut1File                //Create source data for VGlut1 intensities
```

```
VGlut1_Int_Matrix= new Matrix()
VGlut1File= new File()
VGlut1File.ropen(vglut1intensities)
VGlut1_Int_Matrix.scanf(VGlut1File, 100, 9)

objref  VGAT_Int_Matrix, VGATFile               //Create source data for VGAT intensities
VGAT_Int_Matrix= new Matrix()
VGATFile= new File()
VGATFile.ropen(vgatintensities)
VGAT_Int_Matrix.scanf(VGATFile, 100, 9)

objref morphMatrix, morphFile                   //Create source data for arbor morphology
morphMatrix = new Matrix()
morphFile = new File()
morphFile.ropen(morphologydata)
morphMatrix.scanf(morphFile,63,37)

/**************************
*Run through each model
*for each cell trials no.
*of times          *
***************************/

for tr=0, tr=trials-1 {
    cellCounter=0
    sprint(filename, "%d%s%s%s%s", randSeed, rawdatafile, model, trailing, extension)
    print filename
    Raw_Data = new Matrix(cells+2, 5)
    Raw_Data_File = new File(filename)
    Raw_Data_File.wopen(filename)
    for i=cellStart-1, i=cellEnd-1 {
        xopen("Build Arbor Topologies Apical.hoc")
        xopen("Specify Geometry Apical.hoc")
        finitialize(-70)
        xopen("Weighted ExcitInhib Apical.hoc")
        cellCounter+=1
    }
    Raw_Data.fprint(Raw_Data_File)
    Raw_Data_File.close()
}


/**************************
*BDNF Setup        *
***************************/
strdef weight, dist, rawdatafile, extension, weightexcit, filename
strdef vglut1intensities, vgatintensities, morphologydata   //String defs to allow the same
model to be
                                //run with different source data (ie Veh or BDNF)
dist = "BDNF Dist Matrix.txt"
weight = "BDNF Weight Matrix.txt"
weightexcit = "BDNF Weight Matrix Excit.txt"
vglut1intensities= "BDNF VGlut1 Ints.txt"           //The name of the matrix for the VGlut1
intensities
vgatintensities= "BDNF VGAT Ints.txt"               //The name of the matrix for the VGAT
intensities
morphologydata="BDNF Morph MM.txt"              //The name of the matrix for the morphology
parameters (ie, no. dendrites and diameters)
rawdatafile = "_BDNF"               //The name of the raw output file
extension = ".dat"

objref Raw_Data, Raw_Data_File                  //Create a destination file for raw data
generated during run

objref VGlut1_Int_Matrix, VGlut1File                //Create source data for VGlut1 intensities
VGlut1_Int_Matrix= new Matrix()
VGlut1File= new File()
VGlut1File.ropen(vglut1intensities)
VGlut1_Int_Matrix.scanf(VGlut1File, 100, 9)
```

```
objref  VGAT_Int_Matrix, VGATFile                //Create source data for VGAT intensities
VGAT_Int_Matrix= new Matrix()
VGATFile= new File()
VGATFile.ropen(vgatintensities)
VGAT_Int_Matrix.scanf(VGATFile, 100, 9)

objref morphMatrix, morphFile                    //Create source data for arbor morphology
morphMatrix = new Matrix()
morphFile = new File()
morphFile.ropen(morphologydata)
morphMatrix.scanf(morphFile,63,37)

/**************************
*Run through each model
*for each cell trials no.
*of times         *
**************************/
for tr=0, tr=trials-1 {
    cellCounter=0
    sprint(filename, "%d%s%s%s%s", randSeed, rawdatafile, model, trailing, extension)
    print filename
    Raw_Data = new Matrix(cells+2, 5)
    Raw_Data_File = new File(filename)
    Raw_Data_File.wopen(filename)
    for i=cellStart-1, i=cellEnd-1 {
        xopen("Build Arbor Topologies Apical.hoc")
        xopen("Specify Geometry Apical.hoc")
        finitialize(-70)
        xopen("Weighted ExcitInhib Apical.hoc")
        cellCounter+=1
    }
    Raw_Data.fprint(Raw_Data_File)
    Raw_Data_File.close()
}

naCond=0.03*4
kCond=0.0009*4
apicalVal=250*1
exDelay=4*1
inDelay=4*1
burstdur=100*1

model= "PassDend"
trailing = "_4_1_1_1_1"

/**************************
*Vehicle Setup        *
**************************/
strdef weight, dist, rawdatafile, extension, weightexcit, filename
strdef vglut1intensities, vgatintensities, morphologydata
dist = "Veh Dist Matrix.txt"
weight = "Veh Weight Matrix.txt"
weightexcit = "Veh Weight Matrix Excit.txt"
vglut1intensities= "Vehicle VGlut1 Ints.txt"
vgatintensities= "Vehicle VGAT Ints.txt"
morphologydata="Vehicle Morph MM.txt"
rawdatafile = "_Vehicle"
extension = ".dat"

objref Raw_Data, Raw_Data_File                   //Create a destination file for raw data
generated during run


objref VGlut1_Int_Matrix, VGlut1File                 //Create source data for VGlut1 intensities
VGlut1_Int_Matrix= new Matrix()
VGlut1File= new File()
VGlut1File.ropen(vglut1intensities)
VGlut1_Int_Matrix.scanf(VGlut1File, 100, 9)
```

```
objref  VGAT_Int_Matrix, VGATFile               //Create source data for VGAT intensities
VGAT_Int_Matrix= new Matrix()
VGATFile= new File()
VGATFile.ropen(vgatintensities)
VGAT_Int_Matrix.scanf(VGATFile, 100, 9)

objref morphMatrix, morphFile                   //Create source data for arbor morphology
morphMatrix = new Matrix()
morphFile = new File()
morphFile.ropen(morphologydata)
morphMatrix.scanf(morphFile,63,37)

/**************************
*Run through each model
*for each cell trials no.
*of times          *
***************************/

for tr=0, tr=trials-1 {
    cellCounter=0
    sprint(filename, "%d%s%s%s%s", randSeed, rawdatafile, model, trailing, extension)
    print filename
    Raw_Data = new Matrix(cells+2, 5)
    Raw_Data_File = new File(filename)
    Raw_Data_File.wopen(filename)
    for i=cellStart-1, i=cellEnd-1 {
        xopen("Build Arbor Topologies Apical.hoc")
        xopen("Specify Geometry Apical.hoc")
        finitialize(-70)
        xopen("Weighted ExcitInhib Apical.hoc")
        cellCounter+=1
    }
    Raw_Data.fprint(Raw_Data_File)
    Raw_Data_File.close()
}


/**************************
*BDNF Setup        *
***************************/
strdef weight, dist, rawdatafile, extension, weightexcit, filename
strdef vglut1intensities, vgatintensities, morphologydata   //String defs to allow the same
model to be
                                //run with different source data (ie Veh or BDNF)
dist = "BDNF Dist Matrix.txt"
weight = "BDNF Weight Matrix.txt"
weightexcit = "BDNF Weight Matrix Excit.txt"
vglut1intensities= "BDNF VGlut1 Ints.txt"           //The name of the matrix for the VGlut1
intensities
vgatintensities= "BDNF VGAT Ints.txt"               //The name of the matrix for the VGAT
intensities
morphologydata="BDNF Morph MM.txt"              //The name of the matrix for the morphology
parameters (ie, no. dendrites and diameters)
rawdatafile = "_BDNF"               //The name of the raw output file
extension = ".dat"

objref Raw_Data, Raw_Data_File                  //Create a destination file for raw data
generated during run

objref VGlut1_Int_Matrix, VGlut1File                //Create source data for VGlut1 intensities
VGlut1_Int_Matrix= new Matrix()
VGlut1File= new File()
VGlut1File.ropen(vglut1intensities)
VGlut1_Int_Matrix.scanf(VGlut1File, 100, 9)

objref  VGAT_Int_Matrix, VGATFile               //Create source data for VGAT intensities
VGAT_Int_Matrix= new Matrix()
VGATFile= new File()
```

```
VGATFile.ropen(vgatintensities)
VGAT_Int_Matrix.scanf(VGATFile, 100, 9)

objref morphMatrix, morphFile                    //Create source data for arbor morphology
morphMatrix = new Matrix()
morphFile = new File()
morphFile.ropen(morphologydata)
morphMatrix.scanf(morphFile,63,37)

/***************************
*Run through each model
*for each cell trials no.
*of times           *
***************************/
for tr=0, tr=trials-1 {
    cellCounter=0
    sprint(filename, "%d%s%s%s%s", randSeed, rawdatafile, model, trailing, extension)
    print filename
    Raw_Data = new Matrix(cells+2, 5)
    Raw_Data_File = new File(filename)
    Raw_Data_File.wopen(filename)
    for i=cellStart-1, i=cellEnd-1 {
        xopen("Build Arbor Topologies Apical.hoc")
        xopen("Specify Geometry Apical.hoc")
        finitialize(-70)
        xopen("Weighted ExcitInhib Apical.hoc")
        cellCounter+=1
    }
    Raw_Data.fprint(Raw_Data_File)
    Raw_Data_File.close()
}


/*
Build Arbor Topologies Apical.hoc

This file builds the model neuron
*/
//
objref shollVec,  cumShollVec
shollVec= new Vector(8)
cumShollVec= new Vector(8)

shollVec.x[0]=morphMatrix.x[i][0]
shollVec.x[1]=morphMatrix.x[i][1]
shollVec.x[2]=morphMatrix.x[i][2]
shollVec.x[3]=morphMatrix.x[i][3]
shollVec.x[4]=morphMatrix.x[i][4]
shollVec.x[5]=morphMatrix.x[i][5]
shollVec.x[6]=morphMatrix.x[i][6]
shollVec.x[7]=morphMatrix.x[i][7]

cumShollVec.x[0]=shollVec.x[0]
cumShollVec.x[1]=cumShollVec.x[0]+shollVec.x[1]
cumShollVec.x[2]=cumShollVec.x[1]+shollVec.x[2]
cumShollVec.x[3]=cumShollVec.x[2]+shollVec.x[3]
cumShollVec.x[4]=cumShollVec.x[3]+shollVec.x[4]
cumShollVec.x[5]=cumShollVec.x[4]+shollVec.x[5]
cumShollVec.x[6]=cumShollVec.x[5]+shollVec.x[6]
cumShollVec.x[7]=cumShollVec.x[6]+shollVec.x[7]
//

//
dends=shollVec.sum()
create soma, dend[dends], axon, apical


connect axon(0), soma(0)
dendCounter=0
```

```
for ii=0, ii=shollVec.x[0]-1 {
    connect dend[dendCounter](0), soma(1)
    dendCounter += 1
}
counter = 0
for ii=0, ii=shollVec.x[1]-1 {
    connect dend[dendCounter](0), dend[counter](1)
    if (counter<cumShollVec.x[0]-1) {
        counter += 1
    } else {
        counter = 0
    }
    dendCounter += 1
}
counter = cumShollVec.x[0]
for ii=0, ii=shollVec.x[2]-1 {
    connect dend[dendCounter](0), dend[counter](1)
    if (counter<cumShollVec.x[1]-1) {
        counter += 1
    } else {
        counter = cumShollVec.x[0]
    }
    dendCounter += 1
}
counter = cumShollVec.x[1]
for ii=0, ii=shollVec.x[3]-1 {
    connect dend[dendCounter](0), dend[counter](1)
    if (counter<cumShollVec.x[2]-1) {
        counter += 1
    } else {
        counter = cumShollVec.x[1]
    }
    dendCounter += 1
}
counter = cumShollVec.x[2]
for ii=0, ii=shollVec.x[4]-1 {
    connect dend[dendCounter](0), dend[counter](1)
    if (counter<cumShollVec.x[3]-1) {
        counter += 1
    } else {
        counter = cumShollVec.x[2]
    }
    dendCounter += 1
}
counter = cumShollVec.x[3]
for ii=0, ii=shollVec.x[5]-1 {
    connect dend[dendCounter](0), dend[counter](1)
    if (counter<cumShollVec.x[4]-1) {
        counter += 1
    } else {
        counter = cumShollVec.x[3]
    }
    dendCounter += 1
}
counter = cumShollVec.x[4]
for ii=0, ii=shollVec.x[6]-1 {
    connect dend[dendCounter](0), dend[counter](1)
    if (counter<cumShollVec.x[5]-1) {
        counter += 1
    } else {
        counter = cumShollVec.x[4]
    }
    dendCounter += 1
}
counter = cumShollVec.x[5]
for ii=0, ii=shollVec.x[7]-1 {
    connect dend[dendCounter](0), dend[counter](1)
    if (counter<cumShollVec.x[6]-1) {
        counter += 1
```

```
    } else {
        counter = cumShollVec.x[5]
    }
    dendCounter += 1
}

//
objref sholl[9]
for ii=0, ii=8 {
    sholl[ii]= new SectionList()
}
soma sholl[0].append()
for ii=0,                 ii=cumShollVec.x[0]-1     dend[ii] sholl[1].append()
for ii=cumShollVec.x[0], ii=cumShollVec.x[1]-1     dend[ii] sholl[2].append()
for ii=cumShollVec.x[1], ii=cumShollVec.x[2]-1     dend[ii] sholl[3].append()
for ii=cumShollVec.x[2], ii=cumShollVec.x[3]-1     dend[ii] sholl[4].append()
for ii=cumShollVec.x[3], ii=cumShollVec.x[4]-1     dend[ii] sholl[5].append()
for ii=cumShollVec.x[4], ii=cumShollVec.x[5]-1     dend[ii] sholl[6].append()
for ii=cumShollVec.x[5], ii=cumShollVec.x[6]-1     dend[ii] sholl[7].append()
for ii=cumShollVec.x[6], ii=cumShollVec.x[7]-1     dend[ii] sholl[8].append()

create apical[5]

connect apical[0](0),soma(1)
connect apical[1](0),apical[0](1)
connect apical[2](0),apical[0](1)
connect apical[3](0),apical[0](1)
connect apical[4](0),apical[0](1)

objref apicalList
apicalList=new SectionList()

for ii=0, ii=4 apical[ii] apicalList.append

//


/*
Specify Geometry Apical.hoc

This file builds the model neuron
*/
proc celldef() {
  geom()
  biophys()
  geom_nseg()
}

objref diamVec
diamVec= new Vector(9)

diamVec.x[0]=morphMatrix.x[i][8]
diamVec.x[1]=morphMatrix.x[i][9]
diamVec.x[2]=morphMatrix.x[i][10]
diamVec.x[3]=morphMatrix.x[i][11]
diamVec.x[4]=morphMatrix.x[i][12]
diamVec.x[5]=morphMatrix.x[i][13]
diamVec.x[6]=morphMatrix.x[i][14]
diamVec.x[7]=morphMatrix.x[i][15]
diamVec.x[8]=morphMatrix.x[i][16]

proc geom() {
  forsec sholl[1] {  L = 6.25  diam = diamVec.x[1]  }
  forsec sholl[2] {  L = 6.25  diam = diamVec.x[2]  }
  forsec sholl[3] {  L = 6.25  diam = diamVec.x[3]  }
  forsec sholl[4] {  L = 6.25  diam = diamVec.x[4]  }
  forsec sholl[5] {  L = 6.25  diam = diamVec.x[5]  }
  forsec sholl[6] {  L = 6.25  diam = diamVec.x[6]  }
  forsec sholl[7] {  L = 6.25  diam = diamVec.x[7]  }
```

```
  forsec sholl[8] {  L = 6.25  diam = diamVec.x[8]  }
  soma {  L = diamVec.x[0]  diam = diamVec.x[0] }
  axon {  L = 100 diam = 1   }
  apical[0] {  L = apicalVal diam = 3   }
  apical[1] {  L = apicalVal/5 diam = 1.5   }
  apical[2] {  L = apicalVal/5 diam = 1.5   }
  apical[3] {  L = apicalVal/5 diam = 1.5   }
  apical[4] {  L = apicalVal/5 diam = 1.5   }

}
proc geom_nseg() {
   forsec sholl[1] { nseg = int((L/(0.1*lambda_f(100))+.999)/2)*2 + 1}
   forsec sholl[2] { nseg = int((L/(0.1*lambda_f(100))+.999)/2)*2 + 1}
   forsec sholl[3] { nseg = int((L/(0.1*lambda_f(100))+.999)/2)*2 + 1}
   forsec sholl[4] { nseg = int((L/(0.1*lambda_f(100))+.999)/2)*2 + 1}
   forsec sholl[5] { nseg = int((L/(0.1*lambda_f(100))+.999)/2)*2 + 1}
   forsec sholl[6] { nseg = int((L/(0.1*lambda_f(100))+.999)/2)*2 + 1}
   forsec sholl[7] { nseg = int((L/(0.1*lambda_f(100))+.999)/2)*2 + 1}
   forsec sholl[8] { nseg = int((L/(0.1*lambda_f(100))+.999)/2)*2 + 1}
   soma { nseg = int((L/(0.1*lambda_f(100))+.999)/2)*2 + 1}
   axon { nseg = int((L/(0.1*lambda_f(100))+.999)/2)*2 + 1}
   forsec apicalList   { nseg = int((L/(0.1*lambda_f(100))+.999)/2)*2 + 1}

}
proc biophys() {
  forsec sholl[1] {
    insert hh
      gnabar_hh = naCond
      gkbar_hh = kCond
      gl_hh = 0.0003
      el_hh = -70
      ena = 50
      ek = -87
    Ra = 105
    cm = 1
    insert pas
      g_pas = 0.001
      e_pas = -70
  }
  forsec sholl[2] {
    insert hh
      gnabar_hh = naCond
      gkbar_hh = kCond
      gl_hh = 0.0003
      el_hh = -70
      ena = 50
      ek = -87
    Ra = 105
    cm = 1
    insert pas
      g_pas = 0.001
      e_pas = -70
  }
  forsec sholl[3] {
    insert hh
      gnabar_hh = naCond
      gkbar_hh = kCond
      gl_hh = 0.0003
      el_hh = -70
      ena = 50
      ek = -87
    Ra = 105
    cm = 1
    insert pas
      g_pas = 0.001
      e_pas = -70
  }
  forsec sholl[4] {
    insert hh
```

```
    gnabar_hh = naCond
    gkbar_hh = kCond
    gl_hh = 0.0003
    el_hh = -70
    ena = 50
    ek = -87
  Ra = 105
  cm = 1
  insert pas
    g_pas = 0.001
    e_pas = -70
}
forsec sholl[5] {
  insert hh
    gnabar_hh = naCond
    gkbar_hh = kCond
    gl_hh = 0.0003
    el_hh = -70
    ena = 50
    ek = -87
  Ra = 105
  cm = 1
  insert pas
    g_pas = 0.001
    e_pas = -70
}
forsec sholl[6] {
  insert hh
    gnabar_hh = naCond
    gkbar_hh = kCond
    gl_hh = 0.0003
    el_hh = -70
    ena = 50
    ek = -87
  Ra = 105
  cm = 1
  insert pas
    g_pas = 0.001
    e_pas = -70
}
forsec sholl[7] {
  insert hh
    gnabar_hh = naCond
    gkbar_hh = kCond
    gl_hh = 0.0003
    el_hh = -70
    ena = 50
    ek = -87
  Ra = 105
  cm = 1
  insert pas
    g_pas = 0.001
    e_pas = -70
}
forsec sholl[8] {
  insert hh
    gnabar_hh = naCond
    gkbar_hh = kCond
    gl_hh = 0.0003
    el_hh = -70
    ena = 50
    ek = -87
  Ra = 105
  cm = 1
  insert pas
    g_pas = 0.001
    e_pas = -70
}
forsec apicalList {
```

```
    insert hh
      gnabar_hh = naCond
      gkbar_hh = kCond
      gl_hh = 0.0003
      el_hh = -70
      ena = 50
      ek = -87
    Ra = 105
    cm = 1
    insert pas
      g_pas = 0.001
      e_pas = -70
  }
  soma {
    insert hh
      gnabar_hh = 0.12
      gkbar_hh = 0.0036
      gl_hh = 0.0003
      el_hh = -70
      ena = 50
      ek = -87
    Ra = 105
    cm = 1
    insert pas
      g_pas = 0.001
      e_pas = -70
  }
  axon {
    insert hh
      gnabar_hh = 0.36
      gkbar_hh = 0.0108
      gl_hh = 0.0003
      el_hh = -70
      ena = 50
      ek = -87
    Ra = 105
    cm = 1
    insert pas
      g_pas = 0.001
      e_pas = -70
  }

}
access soma


celldef()






/*
Weighted ExcitInhib Apical.hoc

This file activates synapses on the model neuron
*/

//Create Distance Synapses
objref Matrix_1, Matrix_1_File
Matrix_1      = new Matrix()
Matrix_1_File = new File()
Matrix_1_File.ropen(weight)
Matrix_1.scanf(Matrix_1_File,63,19)

//Retrieve maximum value from the matrix containing total excitatory synapses
//Need to declare maximum synapse value since object references cannot be within brackets
objref Matrix_1_Vec
Matrix_1_Vec = new Vector(63)
```

```
for ib=0, ib=62 {
    Matrix_1_Vec.x[ib]=Matrix_1.x[i][9]
}
MaxSyns= Matrix_1_Vec.max
objref synapse[MaxSyns] //Declare the maximum number of synapse objects possible


objref apc, somaVoltVec, timeVec, Sholl_Vec
counter=0
for ib=1, ib=9 {// progress from 10% to 100% stimulation
    StimPercent = ib*0.1+0.1
    SynsToStimulate = int(StimPercent*Matrix_1.x[i][9])
    Sholl_Vec = new Vector(SynsToStimulate)
    for ib1=0, ib1=SynsToStimulate-1 {
        randNum.uniform(0,1)
        randSholl = randNum.repick()
        if (randSholl<=Matrix_1.x[i][0])                              {Sholl_Vec.x[ib1]=0}
        if (randSholl>Matrix_1.x[i][0] && randSholl<=Matrix_1.x[i][1]) {Sholl_Vec.x[ib1]=1}
        if (randSholl>Matrix_1.x[i][1] && randSholl<=Matrix_1.x[i][2]) {Sholl_Vec.x[ib1]=2}
        if (randSholl>Matrix_1.x[i][2] && randSholl<=Matrix_1.x[i][3]) {Sholl_Vec.x[ib1]=3}
        if (randSholl>Matrix_1.x[i][3] && randSholl<=Matrix_1.x[i][4]) {Sholl_Vec.x[ib1]=4}
        if (randSholl>Matrix_1.x[i][4] && randSholl<=Matrix_1.x[i][5]) {Sholl_Vec.x[ib1]=5}
        if (randSholl>Matrix_1.x[i][5] && randSholl<=Matrix_1.x[i][6]) {Sholl_Vec.x[ib1]=6}
        if (randSholl>Matrix_1.x[i][6] && randSholl<=Matrix_1.x[i][7]) {Sholl_Vec.x[ib1]=7}
        if (randSholl>Matrix_1.x[i][7] && randSholl<=Matrix_1.x[i][8]) {Sholl_Vec.x[ib1]=8}
    }
    SynCounter=0
    ESynCounter=0
    ISynCounter=0
    for id=0, id=SynsToStimulate-1 {// randomly pick x% synapses to stimulate
        Segs=0
        forsec sholl[Sholl_Vec.x[id]] for (x,0) Segs+=1
        randNum.discunif(1,Segs)
        randSeg               = randNum.repick()
        randNum.uniform(0,burstdur)
        randOnset             = randNum.repick()+20
        randNum.uniform(0,1)
        randConductance       = randNum.repick()
        randNum.uniform(0,1)
        randExcitProb         = randNum.repick()
        ExcitProb             = Matrix_1.x[i][Sholl_Vec.x[id]+10]
        if (randExcitProb<=ExcitProb){
            revPot=0
        } else {
            revPot=-70
        }
        Segs=0
        forsec sholl[Sholl_Vec.x[id]] for(x,0) {
            Segs += 1
            if (Segs==randSeg){
                synapse[SynCounter]       = new Exp2Delay(x)
                synapse[SynCounter].e     = revPot
                    if (revPot==0){
                        value=0
                        value2=0
                        while (value<randConductance){
                            value=VGlut1_Int_Matrix.x[value2][Sholl_Vec.x[id]]
                            value2+=1
                        }
                        conductance=(0.337+0.037*value2)/1000
                        synapse[SynCounter].gmax  = conductance
                        synapse[SynCounter].tau1  = 0.2
                        synapse[SynCounter].tau2  = exDelay
                        ESynCounter+=1


                    } else {
                        value=0
                        value2=0
```

```
                    while (value<randConductance){
                        value=VGAT_Int_Matrix.x[value2][Sholl_Vec.x[id]]
                        value2+=1
                    }
                    conductance=(0.266+0.0266*value2)/1000
                    synapse[SynCounter].gmax  = conductance
                    synapse[SynCounter].tau1  = 0.2
                    synapse[SynCounter].tau2  = inDelay
                    ISynCounter+=1
                }
            synapse[SynCounter].onset = randOnset
            SynCounter+=1
        }
    }
  }
  somaVoltVec = new Vector()
  timeVec = new Vector()
  somaVoltVec.record(&soma.v(0.5))
  timeVec.record(&t)
  tstop = burstdur+30
  axon apc = new APCount(1)
  apc.thresh= -35
  run()
  maxSomaVoltage = somaVoltVec.max(700, (burstdur+30)/.025)-(-70)
  Raw_Data.x[0][counter]=StimPercent
  Raw_Data.x[1][counter]=0
  Raw_Data.x[cellCounter+2][counter]=apc.n
  for ie=0, ie=SynCounter-1 {
      synapse[ie].gmax=0
  }
  print "Stim:", StimPercent, "ESyns:", ESynCounter, "ISyns:", ISynCounter, "Spikes:", apc.n
  ib+=1
  counter+=1
}
```
